# Hippocampal theta sweeps indicate goal direction during navigation

**DOI:** 10.1038/s41593-026-02365-2

**Published:** 2026-07-01

**Authors:** Changmin Yu, Zilong Ji, Jake Ormond, John O’Keefe, Neil Burgess

**Affiliations:** 1 Computational and Biological Learning Lab, Department of Engineering, University of Cambridge, Cambridge, UK; 2 Institute of Cognitive Neuroscience, University College London, London, UK; 3 Queen Square Institute of Neurology, University College London, London, UK; 4 Sainsbury Wellcome Centre for Neural Circuits and Behaviour, University College London, London, UK; 5 Department of Cell and Developmental Biology, University College London, London, UK; 6 Department of Bioengineering, Imperial College London, London, UK

**Keywords:** Hippocampus, Spatial memory, Network models

## Abstract

Successful spatial navigation requires rapid evaluation of potential future trajectories. Hippocampal ‘theta sweeps’, the sequential activation of place cells within individual theta cycles, exhibit predictive dynamics within the ideal timeframe for this role. However, whether these sequences reflect movement-related variables, perceptual targets or more cognitive goal-directed planning remains unresolved. Using data from the ‘Honeycomb’ maze, which dissociates head, movement and goal directions, we found that theta sweeps form vectors toward remembered goal locations independent of the rat’s movement or heading directions. Stronger goal modulation preceded correct navigational choices, establishing the relevance of theta sweeps for spatial planning. A hierarchical continuous attractor network with goal-oriented directional inputs reproduced these findings and made several nontrivial predictions, which we confirmed empirically. Sequential activity during immobility-related sharp-wave ripples was also goal directed and, therefore, more aligned with theta sweeps than with previously experienced trajectories. Our findings identify hippocampal theta sweeps as neural substrates for online goal-directed planning.

## Abstract (web summary)

Yu et al. show that hippocampal theta sweeps encode goal direction during memory-guided navigation, independent of movement or head direction and correlating with performance, providing a neural substrate for online spatial planning.

## Main

**Fig. 1:**
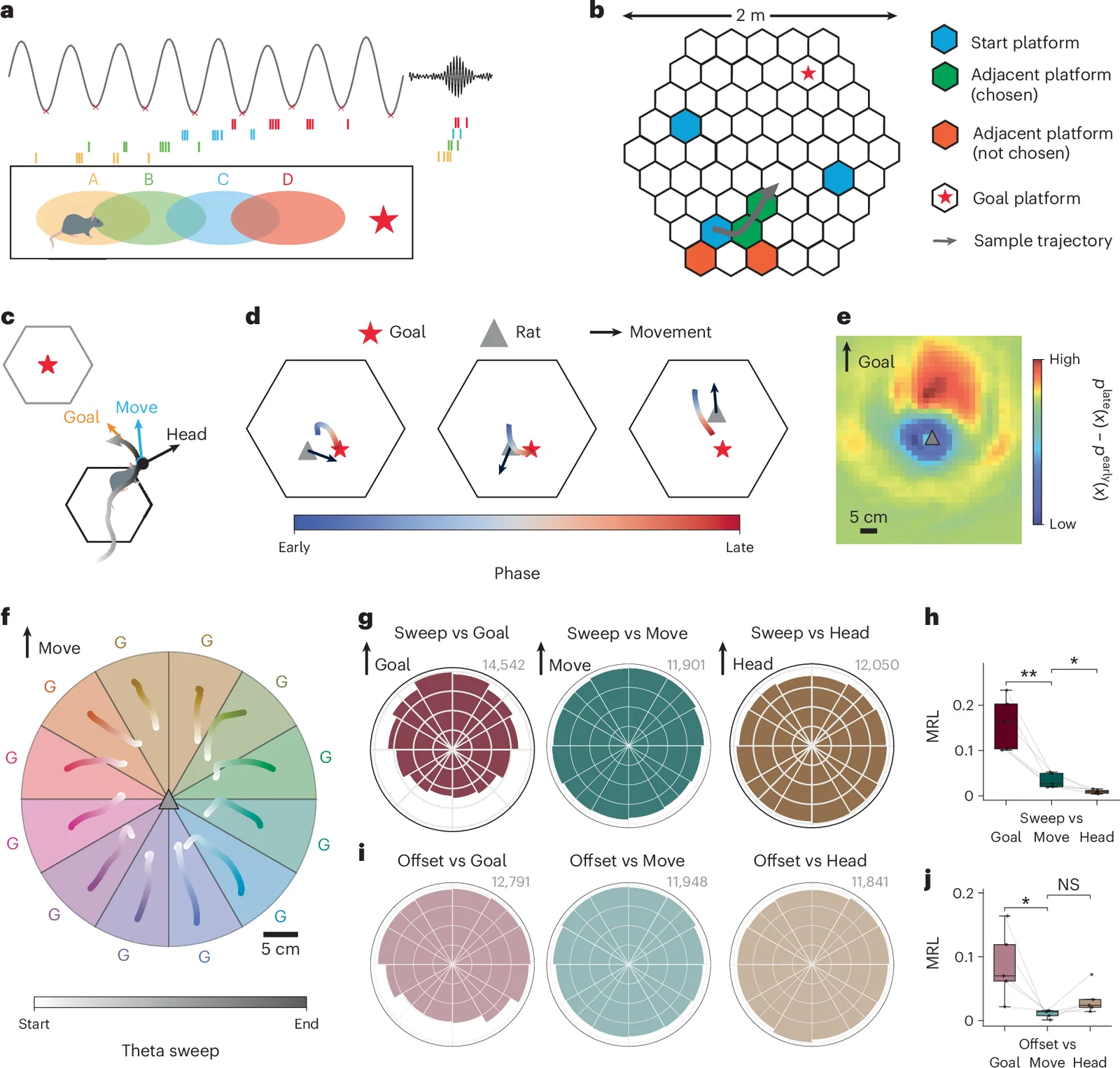
Dissociation of movement and goal direction reveals strong goal-oriented direction bias in hippocampal theta sweeps. **a**, Theta sweeps during movement along linear track (left) and replay sequence during immobility (right). Firing of place cells (A–D) indicated by color (yellow–red). Red star indicates the goal. **b**, The Honeycomb maze^19^. At the beginning of each trial, the rat is placed on a start platform (blue; Extended Data Fig. 1). During each ‘wait period’ of approximately 10 seconds, the animal is confined to its current platform (blue, then green) until two pseudo-randomly chosen adjacent platforms are raised, when it makes its choice by moving onto the chosen platform. The chosen platform becomes the new current platform until the trial ends when the rat reaches the goal platform. **c**, Dissociation of goal direction (Goal), movement direction (Move) and head direction (Head) on the Honeycomb maze. **d**, Example theta sweeps when the rat’s movement direction is aligned, sideways and opposite to the goal direction. **e**, Normalized difference between average decoded posteriors over the first and second halves of theta sweeps, standardized with the rat at the center (gray triangle) and goal direction upwards (black arrow). ***x*** indicates spatial location. **f**, Median decoded theta sweep locations relative to the animal (gray triangle at center) as a function of goal direction (colored ‘G’s) relative to the movement direction (upwards) for each theta cycle of the LFP. **g**, Distributions of relative sweep directions (Sweep) with respect to goal (left), movement (middle) and heading (right) directions. Histograms are constructed given all valid theta sweeps from a single recording session from each of the five recorded rats (one-sided shuffled test against chance level with *n* = 10,000 shuffles; Sweep versus Goal: *P* < 0.0001; Sweep versus Move: *P* = 0.0003; Sweep versus Head: *P* = 0.0038). **h**, Rat-specific mean Rayleigh vector length (MRL) of the distributions in **g** (two-sided paired *t*-tests. *n* = 5 animals; Goal versus Move: *P* = 0.0016, *t* = 4.6537; Move versus Head: *P* = 0.0120, *t* = 3.2334; d.f. = 4), **i**, Same as **g** but with respect to the direction of the initial offset of decoded theta sweeps relative to the animal’s location (Offset) (one-sided shuffled test with *n* = 10,000 shuffles; Offset versus Goal: *P* < 0.0001; Offset versus Move: *P* = 0.0012; Offset versus Head: *P* < 0.0001). **j**, Same as **h** but for distributions in **i** (two-sided paired *t*-tests: Goal versus Move: *P* = 0.0149; *t* = 3.0887; Move versus Head: *P* = 0.0720; *t* = −2.0717). See main text and Extended Data Fig. 4l for LMM results confirming the rat-level test results (**h** and **j**) at the single-sweep level, taking into account the hierarchical structure of the data (nested sweeps within rats). For box plots, center lines indicate medians, with box bounds indicating the interquartile range and whiskers indicating the minimum and maximum values, excluding outliers. d.f., degrees of freedom; NS, not significant. Source data

The ability to transcend immediate sensory inputs and motor outputs to form cognitive vectors toward desired locations is a crucial aspect of successful planning and navigation. Hippocampal place cells, in addition to representing the current location^1^, exhibit remarkable sequential dynamics within an appropriate timeframe for such online planning. During foraging, the population of active place cells sweep forward from those with firing fields at or behind the animal’s current location to those with firing fields ahead of the animal within each cycle of the (6–12 Hz) theta rhythm^2–5^ (‘theta sweeps’; Fig. 1a). Recent results have suggested a cognitive role for theta sweeps. They sample potential paths ahead of the animal, sometimes alternating to both sides of a choice point^6–8^ or of the movement direction in open fields^9,10^; are influenced by locations to avoid^11,12^ and perceptual targets^13^; and extend farther when running faster^8^ or toward more distant^14^ or better-learned^15^ reward locations. However, it remains unclear whether theta sweeps simply sample potential movement trajectories or whether they embody a true allocentric vector toward places of interest, independent of the animal’s current heading, available paths or perceptual targets, consistent with the operation of a cognitive map^1,16,17^. Dissociating these possibilities has been hampered by the restricted sampling of firing fields on narrow tracks and by the animal’s propensity to orient and move directly toward places of interest.

In this study, we investigated the cognitive role of theta sweeps by analyzing place cell firing from hippocampal region CA1 of rats during goal navigation on the ‘Honeycomb’ maze task, which allows dissociation of the animal’s movement and heading directions from the goal direction^18,19^ (Fig. 1b). Our analysis revealed robust goal-oriented directional bias in hippocampal theta sweeps independent of the other variables. Crucially, we found elevated goal modulation preceding correct navigational choices and increasing with experience of the task, suggesting the relevance of theta sweeps for goal-directed planning. We also found goal-directed theta sweeps in data from rats during homing trials on a completely different navigational task—the ‘Cheeseboard’ maze task—where there was a greater number of CA1 place cells recorded but weaker dissociation between movement and goal directions^20^.

The extension of an existing continuous attractor network (CAN) model of theta sweeps^21^, augmented with a top-down goal-directed input, reproduces these empirical findings. Our model exhibits goal-dependent theta phase precession^22^ and anticipatory coding, which we confirmed in the neural data. It also suggests a potential mechanism for the recently discovered goal-oriented egocentric directional tuning in place cells^19^. Goal-directed theta sweeps during movement were complemented by goal-oriented ‘replay’ sequences during immobility^20,23^, suggesting that these latter trajectories are shaped by plasticity during earlier theta sweeps^24,25^. Our findings establish theta sweep as a potential neural substrate for flexible goal-directed online planning; which also supports offline consolidation of goal-directed information.

## Results

### Hippocampal theta sweeps exhibit robust goal-oriented directionality

The ‘Honeycomb’ maze was designed to dissociate movement, head and goal directions^18,19^: rats navigate toward an unmarked goal through a succession of choices between pairs of indirect intermediate platforms (Fig. 1b), often exhibiting rotational scanning behaviors on each platform while waiting for the next choice, effectively dissociating movement, head and goal directions (Fig. 1c).

**Extended Data Fig. 1:**
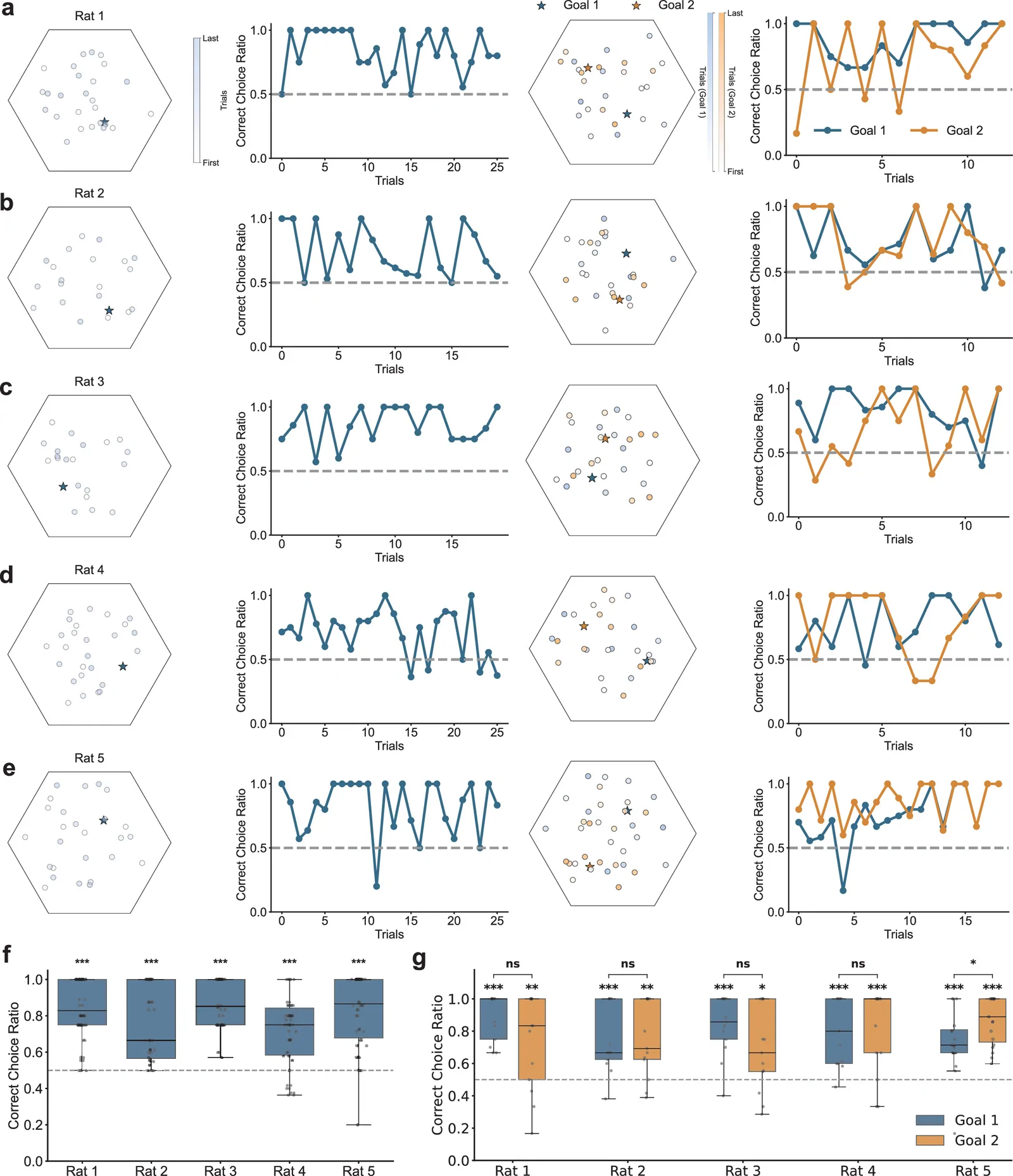
Rats learned to correctly navigate to the goal in the Honeycomb maze. **a**. For Rat 1, from left to right: goal location and trial-specific start locations in the single-goal recording sessions (after successful behavioural training); trial-specific ratio of correct choices; goal locations and corresponding trial-specific start locations for both goals in the goal-switching session; trial-specific ratio of correct choices before and after the goal switching. Note that trials are in block order and the goal-switching only happens once. **b-e**. Same as (a), but for Rats 2–5. Note that Rat 5 was trained over an extended number of training sessions, and here we present the behavioural data from the final session. **f**. Proportions of trial-specific correct choices for each rat in the single-goal session (one-sided one-sample t-test between the trial-specific correct choice ratios and chance level; Rat 1: p = $1.6600\times {10}^{-10}$, t = 9.9828, d.f.=25; Rat 2: p = $1.3010\times {10}^{-5}$, t = 5.5058, d.f.=19; Rat 3: p = $3.5644\times {10}^{-10}$, t = 11.2973, d.f.=19; Rat 4: p = $3.1467\times {10}^{-6}$, t = 5.6922, d.f.=25; Rat 5: p = $3.8588\times {10}^{-8}$, t = 7.4858, d.f.=25). **g**. Same as (f), but separately for the two goal locations in the goal-switching session (two-sided Welch’s two-sample t-test with pooled variances between the trial-specific correct choice ratios for the two set of trials corresponding to different goals; Rat 1: p = 0.1095, t = 1.6869, d.f.=24; Rat 2: p = 0.8706, t = -0.1647, d.f.=24; Rat 3: p = 0.1068, t = 1.6757, d.f.=24; Rat 4: p = 0.8911, t = -0.1384; Rat 5: p = 0.0241, t = -2.3646, d.f.=24). Note that Rat 5 is excluded from all analyses associated with goal-switching experiments due to significantly different navigational performance before and after goal-switching. For boxplots, centre lines indicate medians, with box bounds indicate the interquartile range, and whiskers indicate the minimum and maximum values. Source data

**Extended Data Fig. 2:**
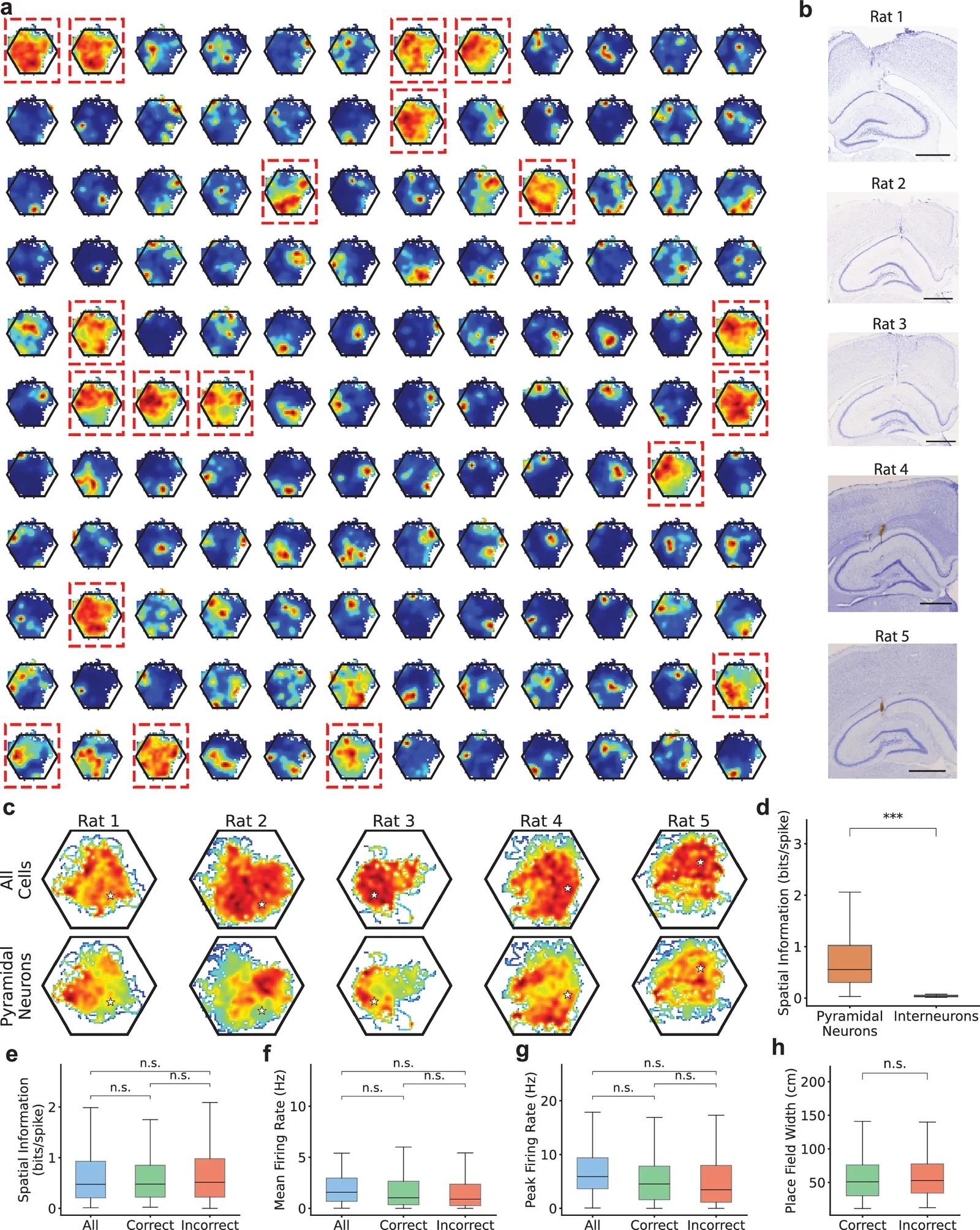
Properties of place cells firing in the Honeycomb maze. **a**. Spatial tuning for all recorded neurons in Rat 2, with Skaggs adaptive smoothing^56^. Cells enclosed by the red rectangular box are classified as interneurons (19 / 132, see Methods), and the rest as putative place cells. **b**. Rat-specific histology showing tetrode marking lesions in the CA1 cell layer in dorsal hippocampus in all 5 experimental subjects (adapted from Ormond and O’Keefe^19^). **c**. Rat-specific aggregated spatial tuning (summation of all cell-specific spatial tuning curves) for all recorded neurons (top), and all putative pyramidal neurons (bottom). White star indicates the goal location. **d**. Cell-specific spatial information (bits per spike^68^; see Methods) for pyramidal neurons and interneurons (one-sided Mann-Whitney U-test: p = $2.2509\times {10}^{-40}$, U = 37836.0, effective size: r = 0.9558, N_pyramidal_ = 526, N_interneuron_ = 77). There is little spatial information in inhibitory interneuron activity, consistent with the addition of interneurons to pyramidal cells resulting in minimal improvement in decoding accuracy (Extended Data Fig. 3b). **e**. Cell-specific spatial information over all valid periods, and ‘wait-periods’ preceding correct and incorrect choices (‘subtrials’), for all recorded neurons (two-sided paired t-test, n = 603 total recorded neurons over 5 animals; all vs correct choices: p = 0.1560, t = 1.4203; all vs incorrect choices: p = 0.1275, t = -1.5262; correct vs incorrect choices: p = 0.3884, t = −0.8632; d.f.= 602). **f-g**. Same comparison as **e**, but for cell-specific peak firing rates (**f**; two-sided paired t-test; all vs correct choices: p = 0.5165, t = 0.6490; all vs incorrect choices: p = 0.1995, t = 1.2843; correct vs incorrect choices: p = 0.2854, t = 1.0688), and mean firing rates over all binned spatial locations (**g**; two-sided paired t-test; all vs correct choices: p = 0.9585, t = 0.0520; all vs incorrect choices: p = 0.4370, t = 0.7777; correct vs incorrect choices: p = 0.2246, t = 1.2150; d.f.=602). **h**. Place field width (see Methods) for all pyramidal neurons over correct and incorrect choices (two-sided paired t-test; p = 0.1074, t = -1.6112, d.f.=525). For boxplots, centre lines indicate medians, with box bounds indicate the interquartile range, and whiskers indicate the minimum and maximum values. Source data

**Extended Data Fig. 3:**
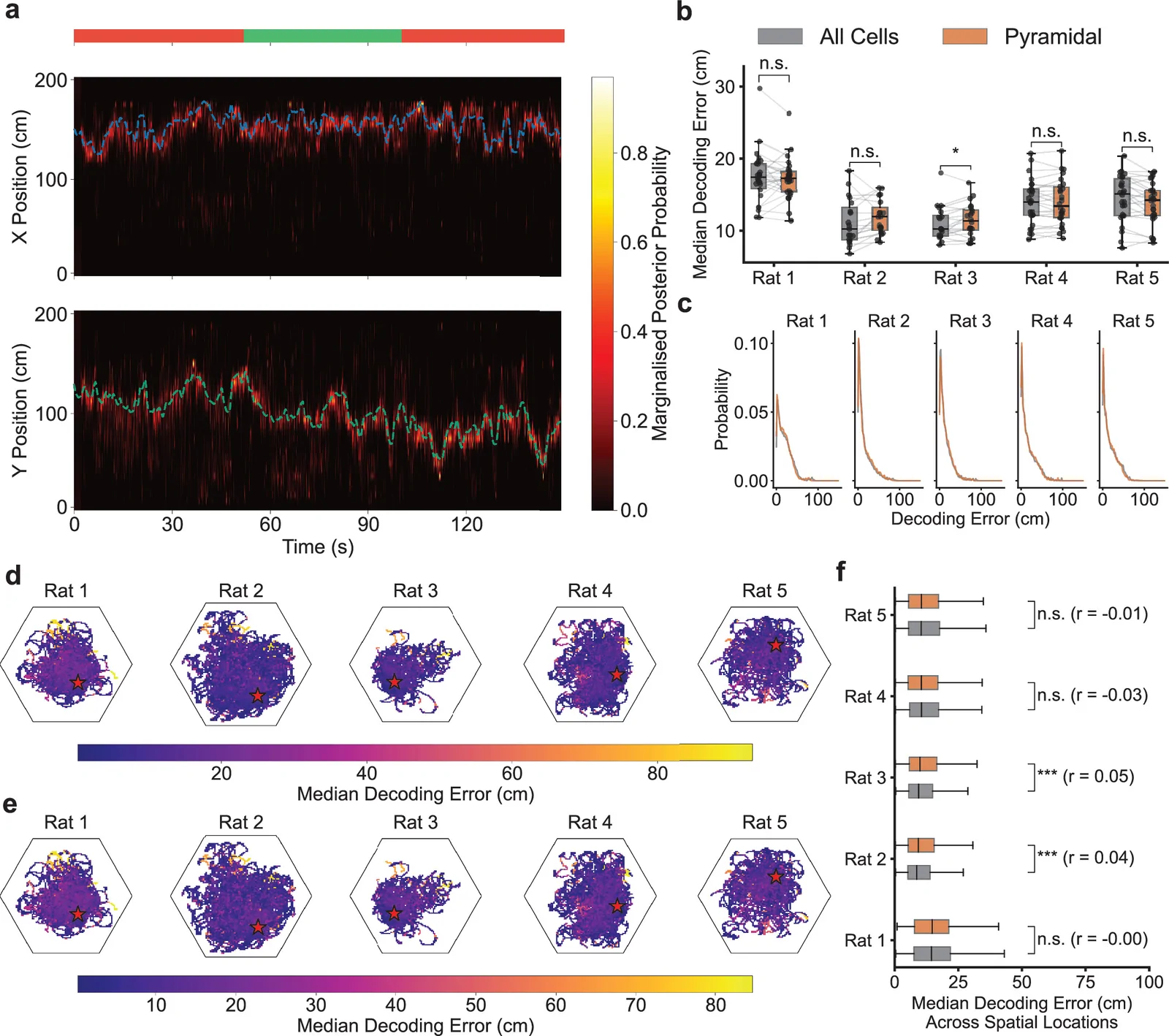
Bayesian decoding of spatial location in the Honeycomb maze. **a**. Example posterior probabilities over a held-out validation trajectory spanning 145 seconds given Bayesian decoding, plotted separately for the x (top) and y (bottom) positions (Rat 2). The probabilities are computed via marginalisation over the orthogonal dimension. Dashed lines represent the ground-truth locations of the rat (blue for x-position and green for y-position). The horizontal bar at the top indicates the choice-correctness of the ongoing ‘subtrial’ (green and red indicate correct and incorrect choices, respectively). Qualitative inspection shows that Bayesian decoding yields accurate inference of the rat’s spatial position (median decoding error: 13.1084 ± 0.5635 cm, 5 rats). **b**. Cross validated median decoding errors (using rate maps computed from all other trials) within each trial, using either all cells (grey) or only pyramidal cells (orange), for all 5 rats (two-sided paired t-test between the two groups of decoding errors; Rat 1: p = 0.9232, t = 0.0956, d.f.=25; Rat 2: p = 0.1319, t = -1.6000, d.f.=19; Rat 3: p = 0.0154, t = -2.6220, d.f.=19; Rat 4: p = 0.6799, t = 0.4175, d.f.=25; Rat 5: p = 0.9941, t = 0.0075, d.f.=25). Note that decoding errors are generally low for both approaches, with only rat 3 showing a small benefit of decoding with all neurons, likely due to its reduced coverage of the spatial environment (Extended Data Fig. 2). **c**. Distribution of decoding errors (aggregating over all trials) given Bayesian decoders with either all cells (grey) or only pyramidal cells (orange). **d**. Median decoding error over spatial locations in the Honeycomb maze, for all 5 rats, given a Bayesian decoder with all cells. Goal location is indicated by the red star. **e**. Same as (d), but using a Bayesian decoder with only pyramidal neurons. **f**. Comparison of median decoding error over spatial locations between the two Bayesian decoders (One-sided Mann-Whitney U test; Rat 1: p = 0.5946, t = $4.6250\times {10}^{6}$, N_1_ = N_2_ = 822; Rat 2: p = 0.0002, t = $8.2463\times {10}^{6}$, N_1_ = N_2_ = 1086; Rat 3: p = 0.0008, t = $2.3982\times {10}^{6}$, N_1_ = N_2_ = 653; Rat 4: p = 0.9722, t = $6.8485\times {10}^{6}$, N_1_ = N_2_ = 979; Rat 5: p = 0.6795, t = $5.5243\times {10}^{6}$, N_1_ = N_2_ = 955), along with corresponding effective size of the test (r). For boxplots, centre lines indicate medians, with box bounds indicate the interquartile range, and whiskers indicate the minimum and maximum values, excluding outliers. Source data

Five rats were trained to reach near-optimal performance prior to electrophysiological recording trials in the Honeycomb maze (Extended Data Fig. 1; see Ormond and O’Keefe^19^ for details). We used the population activity of all recorded CA1 place cells over valid theta cycles (running speed >2 cm s^−1^, theta power greater than 1 s.d. below the mean, 105.2 ± 7.5 place cells per rat; Extended Data Fig. 2) and performed Bayesian decoding of the rat’s spatial location^26^, where decoded location is given by the maximum a posteriori (MAP) estimate (Methods; median decoding error given 200-ms temporal binning: 13.11 ± 2.56 cm; Extended Data Fig. 3). For theta sweep decoding, we used phase-rolling temporal binning, with a 60° phase window (equivalent to 14–28 ms) and overlapping increments of 15° (equivalent to 3.5–7 ms)^27^. Owing to the cyclic nature of theta sweeps, we analyzed the most forward-extending subsequence of decoded spatial locations within each theta cycle extracted from the local field potential (LFP)^9,27^ (Methods). In total, 137,651 theta sweeps were identified from five animals (25,574, 27,696, 16,070, 39,993 and 28,813 for rats 1−5, respectively; one recording session per animal with constant session-specific reward location).

**Extended Data Fig. 4:**
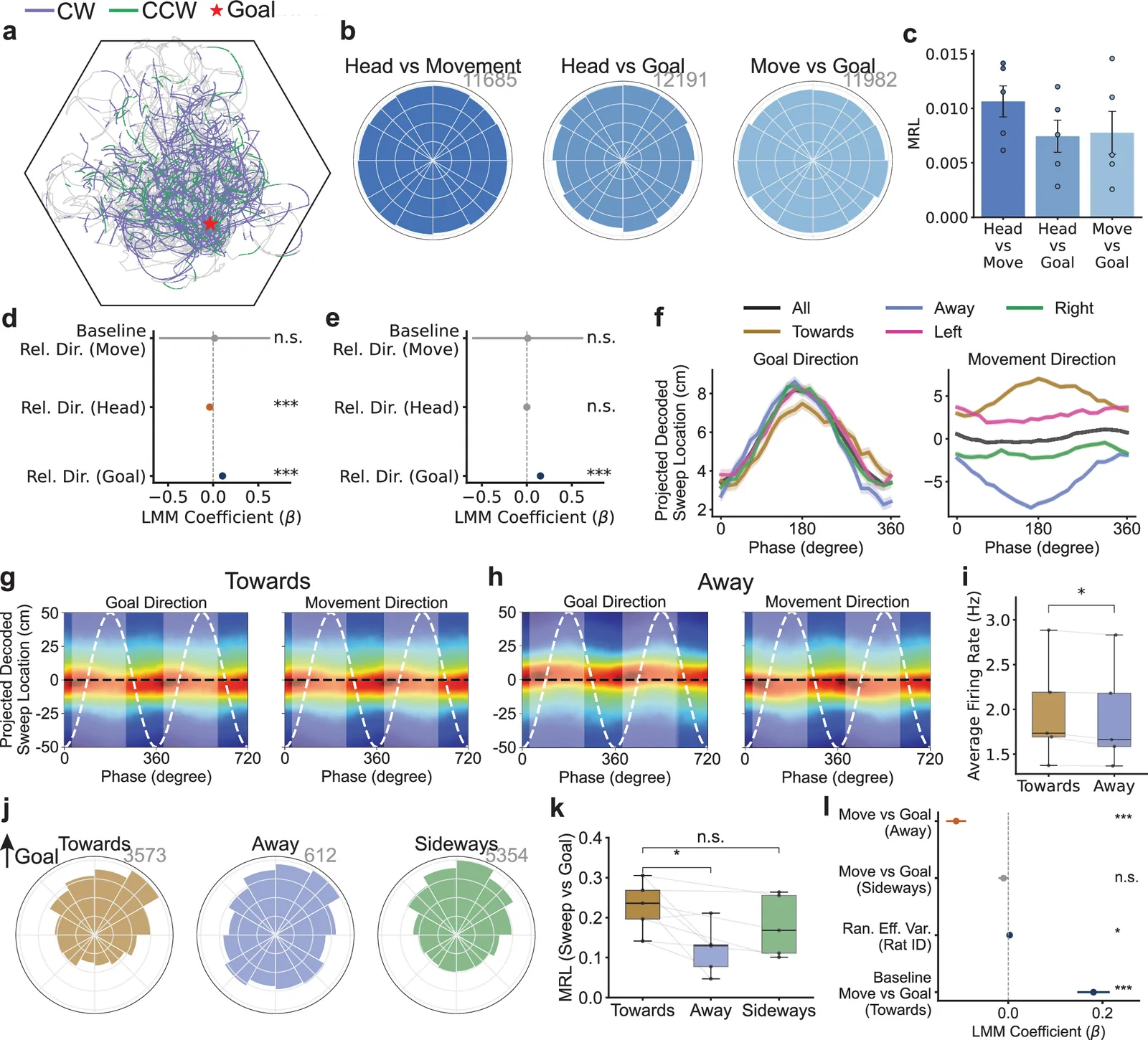
Further details on goal-oriented directional modulations in theta sweeps on the Honeycomb maze. **a**. Position trace over all periods (gray) and over actively scanning periods (magenta and green for CW- and CCW-rotations, respectively), over all trials within the single-goal session of Rat 1. **b**. Circular histograms of relative angles between head, movement, and goal directions in the Honeycomb maze, aggregated over all rats. The behavioural paradigm effectively reduces goalward movement and headings. **c**. Rat-level MRL of relative angles between head, movement, and goal direction (mean ± s.e., n = 5 animals, scatters represent corresponding values for each rat). **d**. Fitted Linear Mixed Model coefficients for predicting cosine similarities between sweep directions and different relative directions, given categorical predictors of relative direction identity (head, movement, goal). With movement direction set as the baseline reference relative direction (n = 137,651 valid theta sweeps; β = 0.0162, z = −0.0518, p = 0.9590), sweep directions were significantly more strongly aligned with goal directions (β = 0.1033, z = -38.5443, p < 10^−308^), but less aligned with head directions (β = −0.0382, z = −14.2363, p = 5.4544 × 10^−46^; cf. Figure 1g, h). **e**. Same as **d**, but for initial offset directions. We again found significantly stronger alignment with goal directions (β = 0.1507, z = −56.1725, p < 10^−308^; baseline reference relative movement direction: β = 0.0097, z = 0.0311, p = 0.9754), and similar level of alignment with head direction (β = 0.0004, z = 0.1671, p = 0.8673; cf. Figure 1i, j). **f**. Decoded theta sweeps, projected onto corresponding goal (left) and movement (right) directions when rats moved towards (330°-30°), away (150°–210^°^), and sideways (left: 240°–300°; right: 60°-120°) to the goal (mean ± 1 s.e.). **g**. Summed decoding posteriors, projected onto corresponding goal (left) and movement (right) directions, when rats were moving towards the goal. **h**. Same as **g**, but for periods when rats were moving away from the goal. **i**. Rat-specific average firing rates (over all cells) when rats were moving towards and away from the goal (one-sided paired t-test, n = 5 animals; p = 0.0238, t = 2.6566; d.f.=4). **j**. Histograms of relative angles between sweep and goal directions (from left to right) when rats were moving towards, away from, and sideways to the goal, respectively (aggregating over all rats; goal direction is upwards). **k**. Rat-specific MRL of the distributions in **j** (two-sided paired t-test, n = 5 animals; Towards vs Away: p = 0.0124, t = 2.7586; Towards vs Sideways: p = 0.1497, t = 1.1097; d.f.=4). **l**. Fitted LMM coefficients predicting the cosine similarity between sweep and goal directions of individual theta sweeps (with corresponding 95% confidence interval), given categorical predictors of animal’s relative movement direction to the goal (towards, away, and sideways). Moving opposite to the goal direction yielded significant decrease in the alignment between sweep and goal directions (baseline: β = 0.1809, z = 10.3086, p = $6.3902\times {10}^{-25}$; opposite: *β* = −0.1104, z = −10.3191, p = 5.8041 × 10^−25^; sideways: *β* = −0.0099, z = −1.8015, p = 0.0717). Directional bias in theta sweeps is jointly modulated by the movement and goal direction, with sweep directions significantly less concentrated around goal directions when animals moved away from the goal, both on the rat- and individual sweep-levels. LMM analysis are reported in forest plots showing fixed-effect coefficients (with 95% confidence interval indicated by the error bar), and corresponding p-values were obtained from two-sided tests of whether each coefficient differed from zero. For boxplots, centre lines indicate medians, with box bounds indicate the interquartile range, and whiskers indicate the minimum and maximum values. Source data

Visual examination of decoded theta sweeps identified a directional bias toward the goal location, regardless of the rat’s movement or head direction (Fig. 1d). We quantified this with several different measures. First, we looked at the difference between the distributions of decoded locations at early and late phases of theta sweeps and found a trough at the animal’s location at early phases and a peak offset toward the goal at late phases (Fig. 1e). A roulette plot, which aggregates theta cycles as a function of goal direction relative to movement direction, clearly showed that theta sweeps indicate goal direction rather than movement direction (Fig. 1f). Comparing the concentration of relative angle distributions (quantified through mean resultant vector length (MRL)) for each individual rat, we found that sweep directions exhibit a strong goal-directed modulation, a moderate movement direction bias and only a weak bias toward head direction (Fig. 1g,h and Extended Data Fig. 4j–l). Finally, after regressing out the effect of movement direction, we found that residual sweep directions were still significantly concentrated toward the goal direction (two-sided shuffled test on MRL of relative angles between sweep and goal directions with *n* = 1,000 shuffles, *P* < 0.001). The direction of the initial offsets of theta sweeps from the rat’s location was also significantly aligned with goal direction and was not modulated by movement or head direction at the level of the individual rat (Fig. 1i,j).

We additionally accounted for the effects of intra-animal variability and tested for these directional modulations at the individual sweep level, by fitting a linear mixed-effects model (LMM) that predicts the cosine similarity between each sweep direction and a set of reference directions (Methods). Similar to the findings from the rat-level analyses, we again found that both sweep and initial offset directions are strongly aligned with goal directions (Extended Data Fig. 4d,e).

To further demonstrate the goal-directed nature of theta sweeps, we projected decoded theta sweeps onto goal and movement directions, respectively. Decoded locations consistently progressed along the goal direction regardless of the movement direction, whereas projections onto movement direction exhibited ‘reversed’ theta sweeps when rats were moving away from the goal and little sweeping when moving orthogonal to the goal direction (Extended Data Fig. 4f). Examining the full decoding posteriors (instead of MAP estimates) yielded similar observations (Extended Data Fig. 4g,h).

Combining the goal directionality of the theta sweeps with the (weaker) movement directionality showed a significantly tighter concentration around the goal direction when animals were moving toward the goal compared to moving away from or at right angles to the goal (Extended Data Fig. 4j), at both the rat level (Extended Data Fig. 4k) and the sweep level (based on LMM analyses as above; Extended Data Fig. 4l).

Goal-oriented directional modulation in theta sweeps was consistent across all five recorded rats (Supplementary Fig. 1) and was robust to alternative decoding procedures such as using the full posterior distributions instead of the MAP estimates (Supplementary Fig. 2) and to alternative methods for extracting theta rhythmicity (multi-unit activity (MUA) instead of LFP; Supplementary Fig. 3).

**Extended Data Fig. 5:**
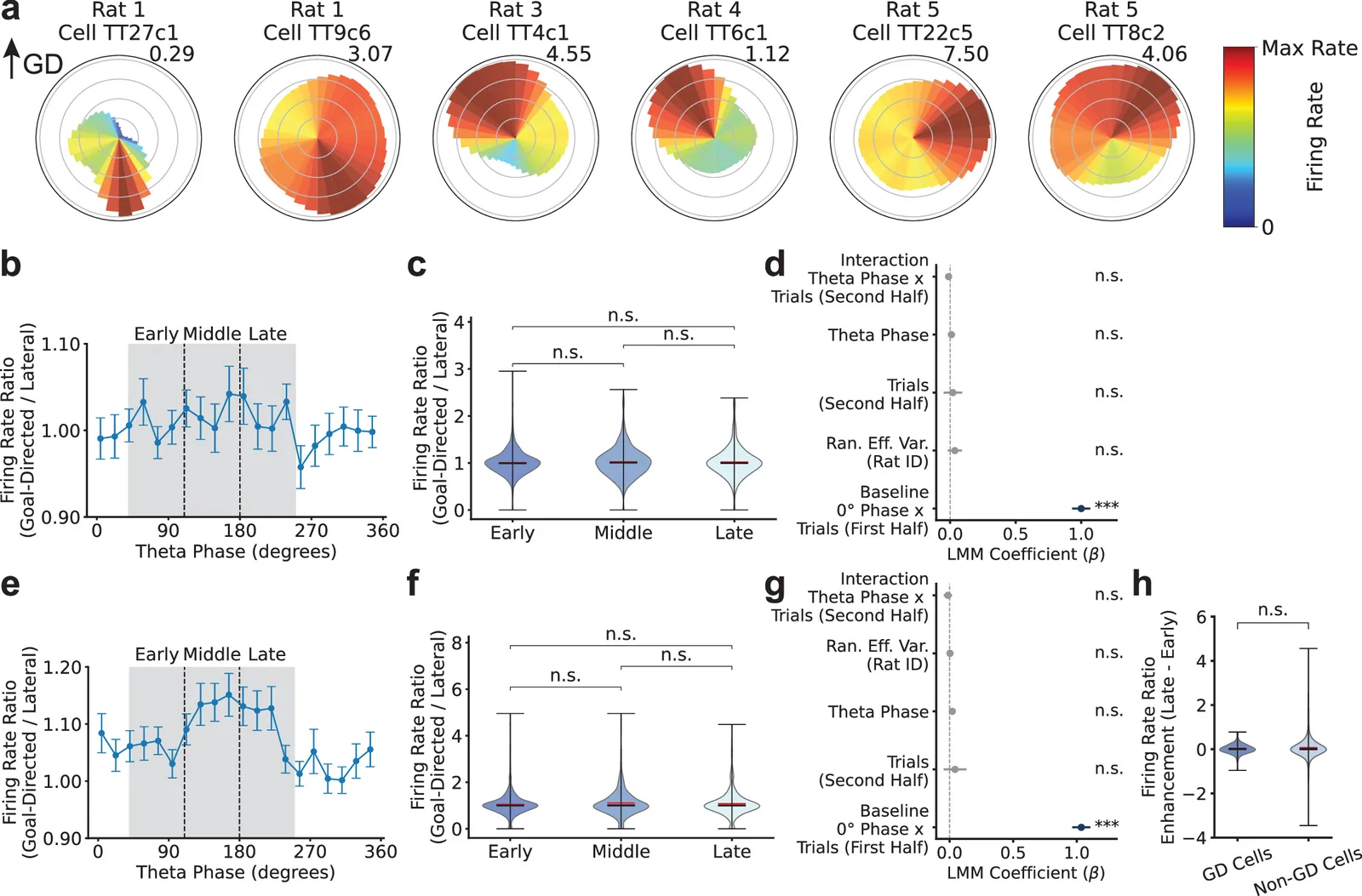
Firing rates of place cells with egocentric goal-directional tuning are not modulated by theta phase or the presence of goal-directed versus non-goal-directed sweeps. **a**. Firing rate maps as a function of relative head direction to goal direction (upwards) for example place cells with significant egocentric goal-directional tuning (total: 139; see Methods). **b**. Ratio of firing rates of all place cells with significant egocentric goal-directional tuning (n = 139), over theta cycles with goal-directed (relative angle between sweep and goal directions within [−45°, 45°] and non goal-directed (lateral) theta sweeps (mean ± 1 s.d.), as a function of theta phase. Shaded region indicates forward extending phases within theta cycles on expectation (50°–260°; cf. Extended Data Fig. 4g–i), and separated into equal-sized early, middle, and late phases. **c**. Distribution of firing rate ratios during early, middle, and late phases within each theta cycle (two-sided Wilcoxon signed-rank test; n = 139 neurons with significant egocentric goal-directional tuning; Early vs Middle: p = 0.3507, t = $7.3618\times {10}^{4}$; Early vs Late: p = 0.2038, t = $7.7039\times {10}^{4}$; Middle vs Late: p = 0.9776, t = $7.2342\times {10}^{4}$). **d**. Fitted LMM coefficients for predicting firing rate ratios, given two predictors: theta phase, and categorical indicator for which half of all trials (first or second) the firing rate ratio was computed from, as well as an interaction term of the two predictors (see Methods). We additionally included rat-level random intercepts to account for within-animal variability. None of the fixed effects showed significant modulation (n = 1,688 observations; Theta Phase: *β* = 0.0106, z = 1.0933, p = 0.2749; Trials (Second Half): *β* = 0.0218, z = 0.5783, p = 0.5632; Interaction: *β* = −0.0121, z = -0.9100, p = 0.3633). Only the baseline intercept was statistically significant (*β* = 1.0006, z = 28.3077, p = 0.0009), indicating that the firing rate ratio remains stable and consistently around 1 across theta phases and learning stages. **e-g**. Same as **b-d**, but for place cells without significant egocentric goal-directional tuning (**f**. two-sided Wilcoxon signed-rank test, n = 387 putative place cells with insignificant egocentric goal-directional tuning; Early vs Middle: p = 0.4998, t = $5.3162\times {10}^{4}$; Early vs Late: p = 0.1254, t = $5.5616\times {10}^{4}$; Middle vs Late: p = 0.1744, t = $5.6883\times {10}^{4}$; **g**. n = 4,644 observations; Theta Phase: *β* = 0.0224, z = 1.8310, p = 0.0672; Trials (Second Half): *β* = 0.0439, z = 0.9404, p = 0.3475; Interaction: *β* = −0.0130, z = -0.7727, p = 0.4408, Baseline Intercept: *β* = 1.0356, z = 28.781, p = $3.3232\times {10}^{-182}$). **h**. Distribution of firing rate ratio differences between late and early phases within theta cycles for place cells with and without significant egocentric goal-directional tuning (two-sided Mann-Whitney U test, n_GD_ = 139, n_non-gd_ = 387 putative place cells with and without significant egocentric goal-directional tuning, respectively; p = 0.7533, t = $2.6413\times {10}^{4}$). The LMM analysis is reported in forest plots showing fixed-effect coefficients (with 95% confidence interval indicated by the error bar), and corresponding p-values were obtained from two-sided tests of whether each coefficient differed from zero. Violin plots show kernel-density estimates of corresponding data distributions, with mean and median values indicated by red and black inner horizontal bars, and whiskers indicate maximum and minimum values. Source data

Could goal-directed theta sweeps be a consequence of goal-related biases in the firing rates of individual neurons? First, we demonstrated that aggregated population rate maps did not overrepresent goal locations (Extended Data Fig. 2c). Next, we analyzed the firing rates of cells with significant egocentric goal-directional tuning^19^ (23.18 ± 0.04% of all recorded neurons across five rats; identified through a linear−nonlinear Poisson model; Methods and Extended Data Fig. 5). If goal-directed sweeps arose from selective amplification of goal-tuned cells, these cells should exhibit elevated firing rates during goal-directed sweeps (that is, sweep direction within 45° of the goal direction) compared to non-goal-directed sweeps and particularly at late phases when sweeps are at their maximum extent. Contrary to this hypothesis, the firing rate ratio between goal-directed and non-goal-directed sweeps remained stable at approximately 1.0 across all theta phases for cells with egocentric goal direction tuning and for all remaining cells (Extended Data Fig. 5b–h). Collectively, these results demonstrate that goal-directed sweeps reflect coordinated population dynamics rather than goal-related firing rate modulations in individual neurons.

In summary, Bayesian decoding of place cell firing during navigation in the Honeycomb maze revealed that theta sweeps exhibit robust goal directionality, independent of the animal’s movement or heading directions. Notably, this goal-oriented modulation arises from coordinated population dynamics rather than phase-selective amplification of individual goal-encoding neurons.

### Additional factors modulate theta sweeps

**Fig. 2:**
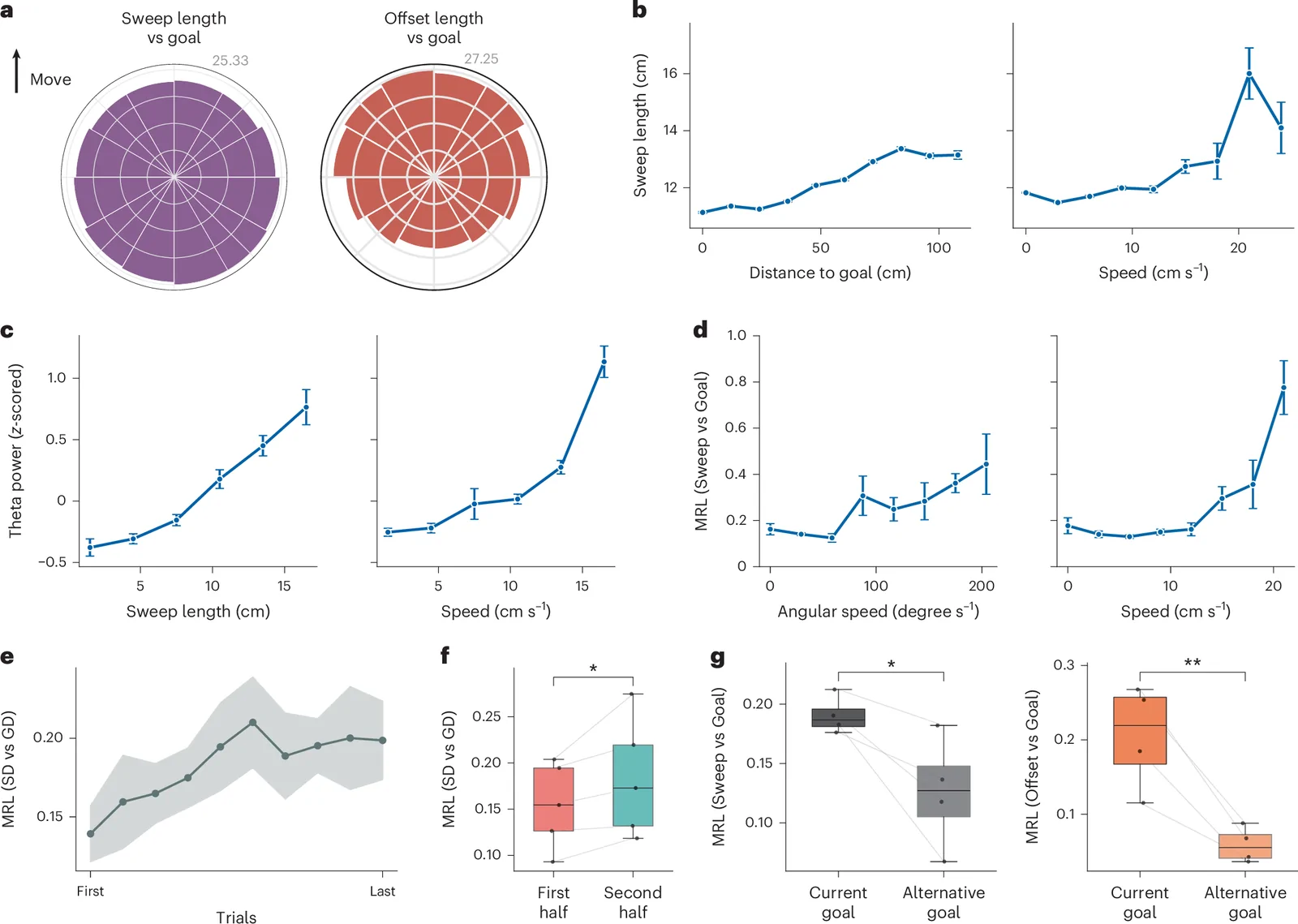
Additional factors modulate theta sweeps. **a**, Median (over all valid sweeps) sweep length (left) and offset length (distance between the start of sweeps and the animal’s location; right) as a function of goal direction relative to movement direction (upward). **b**, Sweep lengths (mean ± s.e. over all *n* = 137,651 valid sweeps) as a function of distance to the goal (left) and animal’s movement speed (right). **c**, Theta power (*z*-transformed; mean ± s.e.) as a function of increasing sweep lengths (left) and movement speed (right). **d**, MRL (mean ± s.e.) of rat-specific distributions of relative angles between sweep and goal directions as a function of increasing angular (head-rotational; left) and linear (right) movement speed. **e**, MRL of the circular distribution of relative angles between sweep and goal directions as a function of trial number (mean ± 1 s.d. over five rats). **f**, MRLs of rat-specific distributions of relative angles between sweep and goal directions, over the first and second halves of all trials (two-sided paired *t*-test, *n* = 5 animals: *P* = 0.0331, *t* = −3.1944; d.f. = 4). See also Extended Data Fig. 6b for LMM analysis that accounted for intra-animal variability. **g**, MRLs of rat-specific distributions of relative sweep (left) and initial offset (right) directions to the current and alternative goal locations after goal switching (one-sided paired *t*-test, *n* = 4 animals; Sweep versus Goal: *P* = 0.0220, *t* = 2.7485; Offset versus Goal: *P* = 0.0060, *t* = 4.5960; d.f. = 3). For box plots, center lines indicate medians, with box bounds indicating the interquartile range and whiskers indicating the minimum and maximum values. d.f., degrees of freedom. Source data

In addition to sweep directions, the lengths and starting locations of sweeps were also goal modulated. Sweeps were shorter when rats were moving toward the goal (reflecting movement during the theta cycle, because the sweep is measured relative to the concurrent location of the rat) and started farther from the rat (reflecting a combined modulation of movement and goal direction on the sweepsʼ starting location) (Fig. 2a). As reported previously^14^, theta sweep length increased with the distance to the goal (Fig. 2b), potentially signaling goal distance as well as direction. They also extended farther during fast movements^8^ (Fig. 2b) and scaled positively with LFP theta power (even after regressing out the effect of speed on theta power^1^; Fig. 2c; two-sided shuffled test with *n* = 1,000 shuffles, *P* < 0.001). The alignment between goal and sweep directions was correlated with both angular and linear movements, becoming significantly stronger during faster movements (Fig. 2d).

**Extended Data Fig. 6:**
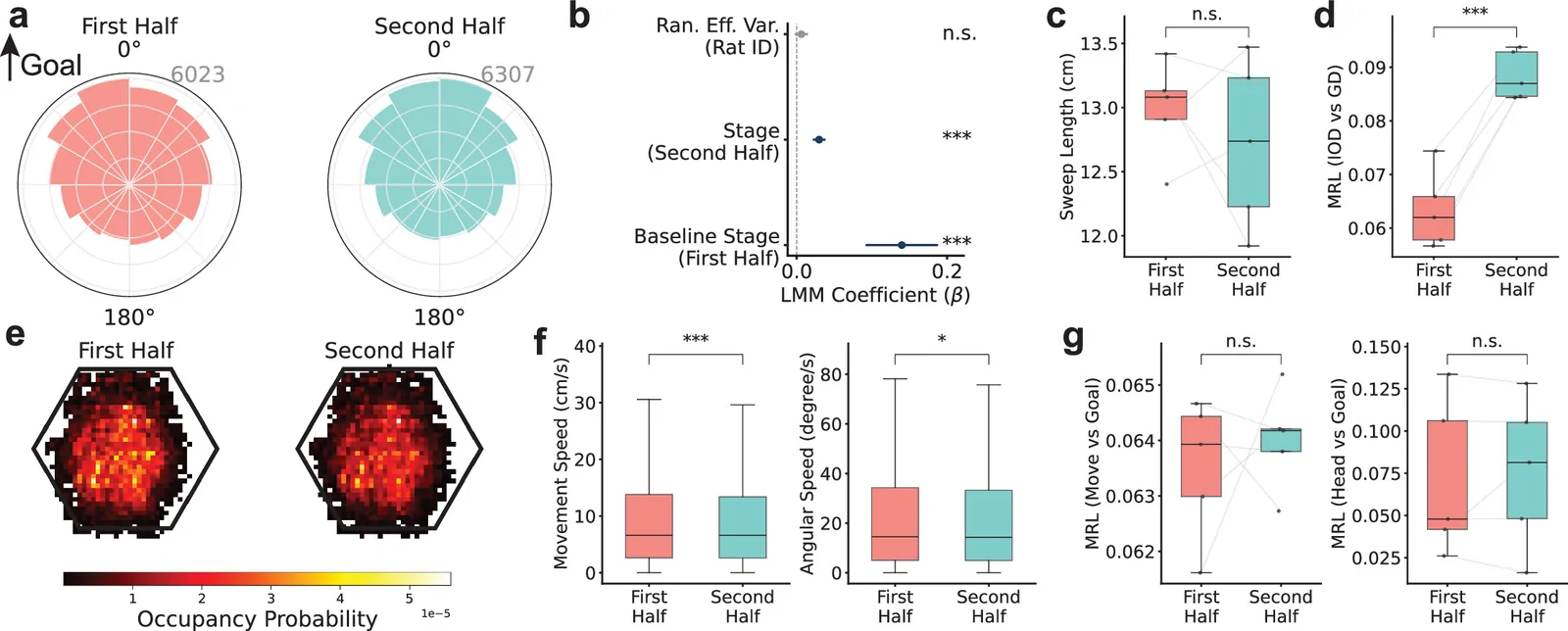
Goal-modulation in theta sweeps becomes stronger with experience. **a**. Circular histograms of relative angles between sweep and goal (upward) directions over the first (left) and second (right) half of all trials (aggregating over all 5 rats; cf. Figure 2f). **b**. Linear Mixed Model coefficients (with 95% confidence interval indicated by the error bar) predicting the cosine similarity between individual sweep directions and corresponding goal directions, given categorical predictors of which half each sweep occurred (n = 137,651 valid theta sweeps; Baseline intercept (first half): *β* = 0.1407, z = 5.7053, p = 0.0010; second half: *β* = 0.0304, z = 6.9913, p = 0.0005; Random effect variance: *β* = 0.0062, z = 1.3879, p = 0.1652). P-values were obtained from two-sided tests of whether each coefficient differed from zero. The LMM analysis indicates that relative to first halves, theta sweeps are significant more strongly aligned with goal directions over second halves within each session. **c**. Rat-specific median length of theta sweeps over the first and second half of all trials (paired two-sided t-test: p = 0.2342, t = 1.3996, d.f.=4). **d**. Rat-specific MRLs of relative angles between initial offset direction and goal direction over the first and second half of all trials (paired two-sided t-test: p = 0.0005, t = 10.3063, d.f.=4). **e**. Spatial occupancy over the first (left) and second (right) half of all trials (aggregated across all rats). The state occupancy distributions over early and late trials are highly similar (Bhattacharyya coefficient, *ρ* = 0.9909). **f**. Movement (left) and rotational (right) speed of rats over the first and second half of all trials (aggregated across all rats). Rats move and rotated significantly faster during early trials comparing to late trials (two-sided Welch two-sample t-test with pooled variance; linear speed: p = $8.8875\times {10}^{-5}$, t = 3.9193; angular speed: p = 0.0134, t = 2.4730; d.f.=116). LMM analysis for predicting linear and angular speed given categorical predictors (first/second half of all trials) whilst instantiating random intercepts for each rat for accounting for within-animal variability yielded similar findings of decreased movement and angular speeds during the second half of all trials (linear speed: baseline (first half): *β* = 9.7178, z = 14.8789, p = $4.5154\times {10}^{-50}$; fixed effect (second half): *β* = −0.2259, z = -3.9578, p = $7.5638\times {10}^{-5}$; random effect variance: *β* = 0.0245, z = 1.4037, p = 0.1604; angular speed: baseline (first half): *β* = 32.4867, z = 5.7054, p = $1.1610\times {10}^{-8}$; fixed effect (second half): *β* = −1.4264, t = -2.4909, p = 0.0127; random effect variance: *β* = 0.0185, z = 1.4099, p = 0.1586). Hence the increased concentration of sweep direction relative to the goal from the first to the second half is not due to speed confound (cf. Figure 2d). **g**. Rat-specific MRLs of relative angles between movement direction and goal direction (left), and between head direction and goal direction (right), over the first and second half of all trials. No significant difference between the spread of the two circular distributions was observed (two-sided paired t-test; Move vs Goal: p = 0.9030, t = 0.1298; Head vs Goal: p = 0.3955, t = 0.9509; d.f.=4). For boxplots, centre lines indicate medians, with box bounds indicate the interquartile range, and whiskers indicate the minimum and maximum values, excluding outliers. Source data

**Extended Data Fig. 7:**
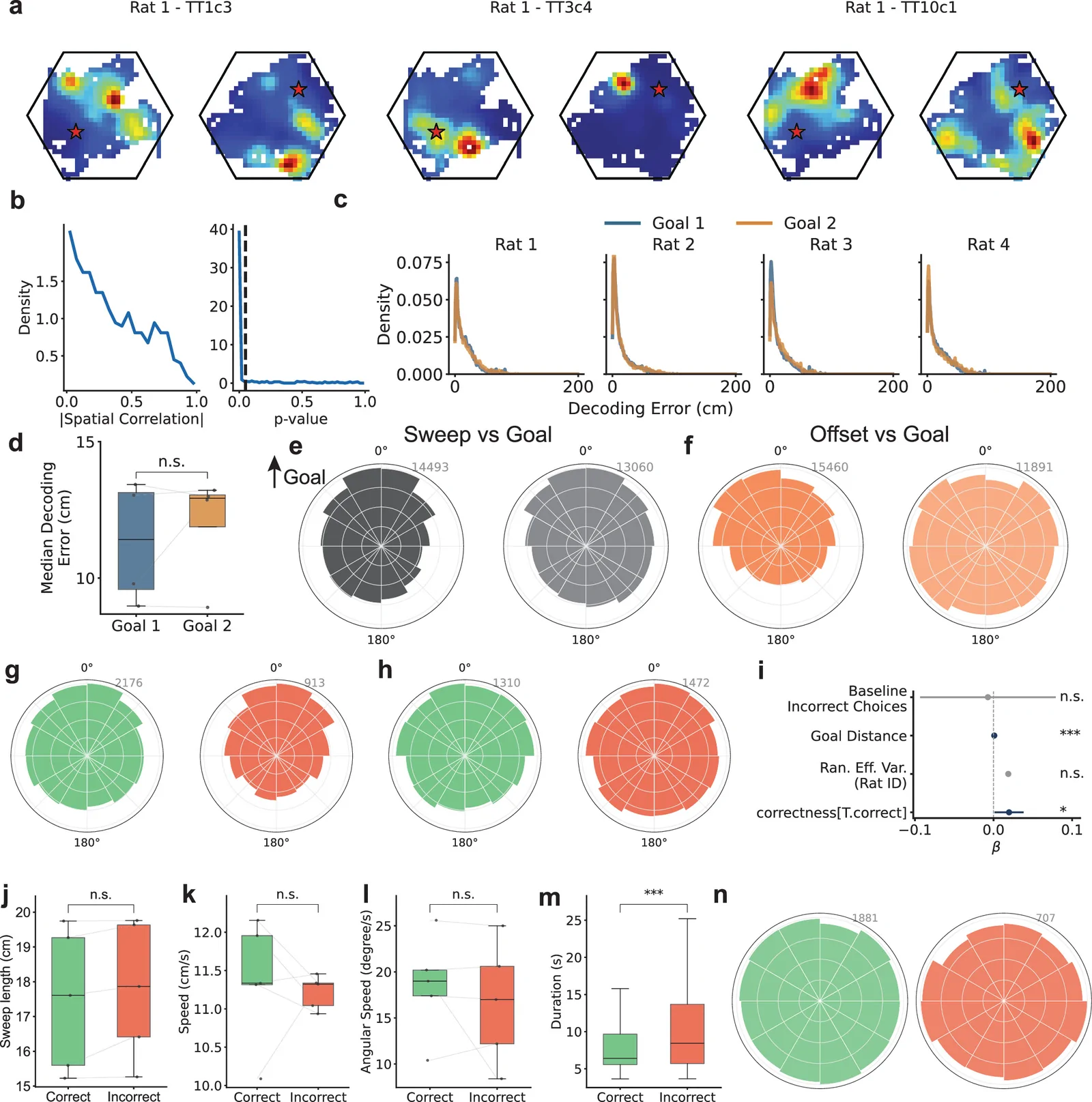
Elevated goal-modulation in theta sweeps to current goals after goal-switching and preceding correct navigational choices. **a**. Three example neurons exhibiting remapping corresponding to switching in goal location (left: before; right: after; red star indicates goal locations). **b**. Probability densities of spatial correlations between rate maps before and after goal-switching (left) and corresponding p-values given two-sided permutation tests against chance-level (right), aggregating all cells from Rats 1–3. There are 359/445 (80.7%) cells exhibiting significant changes (with p < 0.05; two-sided permutation test) in spatial tuning (‘remapping’) after goal-switching. **c**. Probability densities of decoding errors for Rats 1–4 (from left to right) before (Goal 1) and after (Goal 2) goal-switching. **d**. Comparison of rat-specific median decoding error in Goal−1 and Goal-2 trials (two-sided paired t-test, n = 5 animals: p = 0.7733, t = 0.3082, d.f.=4). **e**. Circular histogram of relative angles between the sweep direction, and directions to the current (left) and alternative (right) goal locations (aggregating over all trial before and after goal-switching and over all rats; cf. Figure 2g, left) (one-sided shuffled test against chance-level with n = 10,000 shuffles; current goal: p < 0.0001, alternative goal: p < 0.0001). **f**. Same as (e), but for the relative angles with respect to the direction of initial offset of theta sweeps (cf. Figure 2g, right) (one-sided shuffled test with n = 10,000 shuffles; current goal: p < 0.0001, alternative goal: p = 0.0440). Theta sweep directions and initial offsets preferentially extend towards current goal locations rather than the alternative goal. **g**. Distributions of relative angles between sweep and goal directions (raw data) of all theta sweeps (over 5 rats) extracted from ‘wait-periods’ preceding correct (green) and incorrect (red) choices (one-sided shuffled test against chance-level with 10,000 shuffles; correct choice: p < 0.0001, incorrect choice: p < 0.0001). **h**. Distributions of residual relative angles between sweep and goal directions, after regressing out the effect of distance to the goal^14,53^, over correct (green) and incorrect (red) choices (cf. Figure 3d) (one-sided shuffled test; correct choice: p < 0.0001; incorrect choice: p < 0.0001). **i**. Fitted Linear Mixed Model coefficients (with 95% confidence interval indicated by the error bar) for predicting cosine similarities between sweep direction and goal direction given predictors: correctness of subsequent choice (categorical) and distance to the goal (n = 137,651 valid theta sweeps; baseline incorrect choice: β = -0.0074, z = -0.1643, p = 0.8698; correct choice: β = 0.0207, z = 2.0945, p = 0.0364; goal distance: β = 0.0014, z = 14.5197, p = $9.6908\times {10}^{-48}$). P-values were obtained from two-sided tests of whether each coefficient differed from zero. **j**. Distributions of rat-level mean sweep lengths over correct and incorrect choices (two-sided paired t-test: p = 0.1095, t = -2.0515, d.f.=4). **k-l**. Rat-specific mean movement **(k)** and head-rotational (**l**) speed during correct and incorrect choices (two-sided paired t-test; movement speed: p = 0.3596, t = 1.0339; angular speed: p = 0.2224, t = 1.4434; d.f.=4). **m**. Duration of choice periods over correct and incorrect choices (two-sided paired Welch two-sample t-test with pooled variance: p = 0.0002, t = -3.7194, d.f.=32,136). Rats tend to spend more time before making erroneous decisions, but the frequency of sweep occurrence is similar between correct and incorrect choices (7.6751 sweeps/s and 7.5858 sweeps/s, respectively). **n**. Circular histograms of relative angles between movement and goal directions over correct (green) and incorrect (red) choices. Apart from distance to the goal, there are no additional significant behavioural differences between correct and incorrect choices that may act as potential confounding factors. For boxplots, centre lines indicate medians, with box bounds indicate the interquartile range, and whiskers indicate the minimum and maximum values, excluding outliers. Source data

Goal modulation of theta sweeps increased with experience within sessions, with respect to both sweep direction (Fig. 2e,f and Extended Data Fig. 6a,b) and initial offset (Extended Data Fig. 6d), despite all rats having been extensively trained and showing no significant behavioral improvements across trials within each session (Extended Data Figs. 1 and 6e–g). Both sweep directions and initial offsets were more strongly biased toward the new current goal than toward the previous goal in goal-switching sessions (Fig. 2g and Extended Data Fig. 7a–f).

### Increased goal direction bias precedes correct choices

**Fig. 3:**
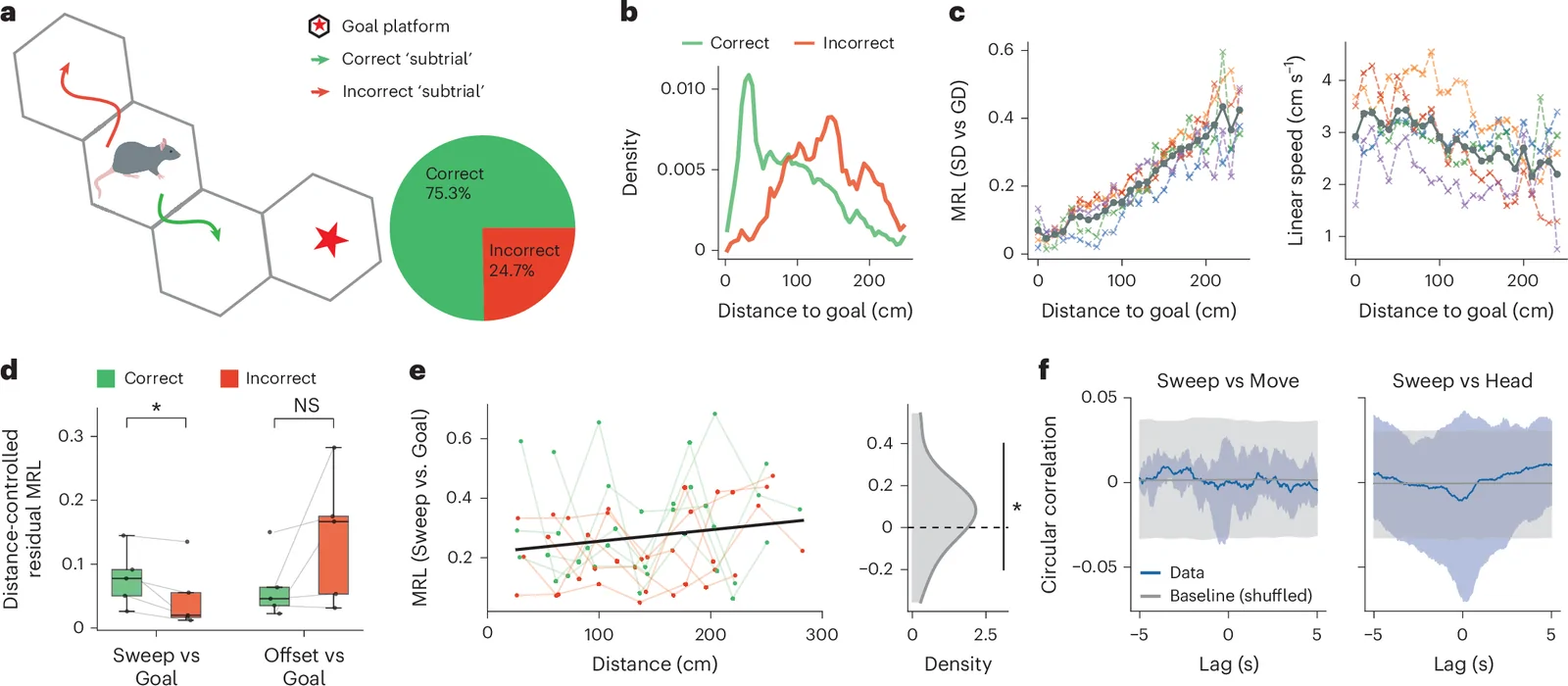
Theta sweeps are more strongly goal modulated prior to correct choices. **a**, Schematic illustration of correct and incorrect ‘choices’. Inset: proportions of correct versus incorrect choices. **b**, Probability density of a rat’s distance to the goal location over correct and incorrect choices. **c**, MRL (mean, black line, over five rats, colors) of sweep direction relative to goal direction (left) and movement speed (right) as a function of distance to the goal (measured at the start of each cycle; Methods). **d**, MRL of rat-specific angles of residual sweep direction and initial offset direction relative to goal direction (after regressing out the effect of distance to the goal in the circular−linear regression; one-sided paired *t*-test, *n* = 5 animals; sweep direction: *P* = 0.0306, *t* = 2.5826; initial offset: *P* = 0.9251, *t* = −1.7796; d.f. = 4). **e**, MRL of relative angles between sweep and goal direction in distance-matched subsamples of correct and incorrect trials (left; black solid line represents best linear fit between MRL and distance to the goal for all data). Difference in residual MRL relative to the regression line between correct and incorrect trials (right; one-sided paired *t*-test: *P* = 0.0136, *t* = 3.5139). **f**, Circular correlation between sweep and movement (left) and head (right) direction as a function of time lag. Shaded regions indicate the 95% confidence interval of the MRLs of the data (blue) and shuffled (gray) distributions (two-sided shuffled test with *n* = 1,000 shuffles; Sweep versus Move: *P* = 1.0 at zero-lag; 19/1,000 of all time lags over five rats with *P* < 0.05, average *P* = 0.4732 ± 0.2553; Sweep versus Head: *P* = 0.687 at zero-lag; 95/1,000 of all time lags over five rats with *P* < 0.05, average *P* = 0.4546 ± 0.3790). For box plots, center lines indicate medians, with box bounds indicating the interquartile range and whiskers indicating the minimum and maximum values, excluding outliers. d.f., degrees of freedom; NS, not significant. Source data

Having established the existence of goalward vectorial information in theta sweeps, we asked if such information could guide the planning as well as the successful execution of goal-directed navigation^6,7^. We analyzed theta sweeps from the final 5-seconds of ‘wait periods’ preceding choices of the next platform prior to correct (choosing the platform nearer the goal) versus incorrect choices (Fig. 3a; 24,200 and 7,938 sweeps, respectively). During this period, before they know the choices available on the upcoming trial, rats often rotate around the platform. Notably, there were no significant behavioral differences between correct and incorrect wait periods (Extended Data Fig. 7k–n), except that the rats made more erroneous choices when farther from the goal (Fig. 3b). Despite this, theta sweeps were more concentrated around the goal direction when the rat was farther from the goal (Fig. 3c), perhaps because the goal platform subtends a narrower range of angles from the animal when farther away. Accordingly, we compared the concentration of theta sweeps around the goal direction prior to correct versus incorrect choices after controlling for the effect of distance to goal, either by parametrically regressing out the distance effect with circular−linear correlation (Methods and Fig. 3d) or by subsampling to equalize the numbers of correct and incorrect trials at each distance (Fig. 3e). We found significantly stronger alignment between residual sweep direction and goal direction preceding correct compared to incorrect choices, whereas initial offsets (Fig. 3d) and sweep lengths did not differ (Extended Data Fig. 7j). There was also a stronger alignment between sweep and goal directions during the same periods at the individual sweep level, based on LMM analysis for predicting cosine similarities between sweep and goal direction, given independent predictors of distance to the goal (continuous) and correctness of subsequent choice (categorical) (Extended Data Fig. 7i). Taken together, these analyses demonstrate the strength of the correlation between the goal-directedness of theta sweeps and subsequent navigational performance, complementing previous findings of increased theta sweep quality with learning of reward location on a circular track^15^.

Notably, no significant correlations between sweep direction and subsequent (or previous) heading or movement directions were found (Fig. 3f), precluding the possibility that goal-directed sweeps simply reflect actual or planned movements. The goal direction bias of theta sweeps increased prior to correct choices and strengthened with experience, suggesting that theta sweeps actively contribute to successful navigation. Collectively, the relevance to cognitive (and not motoric) outcomes, combined with the dynamic encoding of both direction and distance to goals, positions theta sweeps as a potential neural substrate for the vectors underlying flexible online planning.

### Goal-directed theta sweeps during homing trials on the ‘Cheeseboard’ maze

**Fig. 4:**
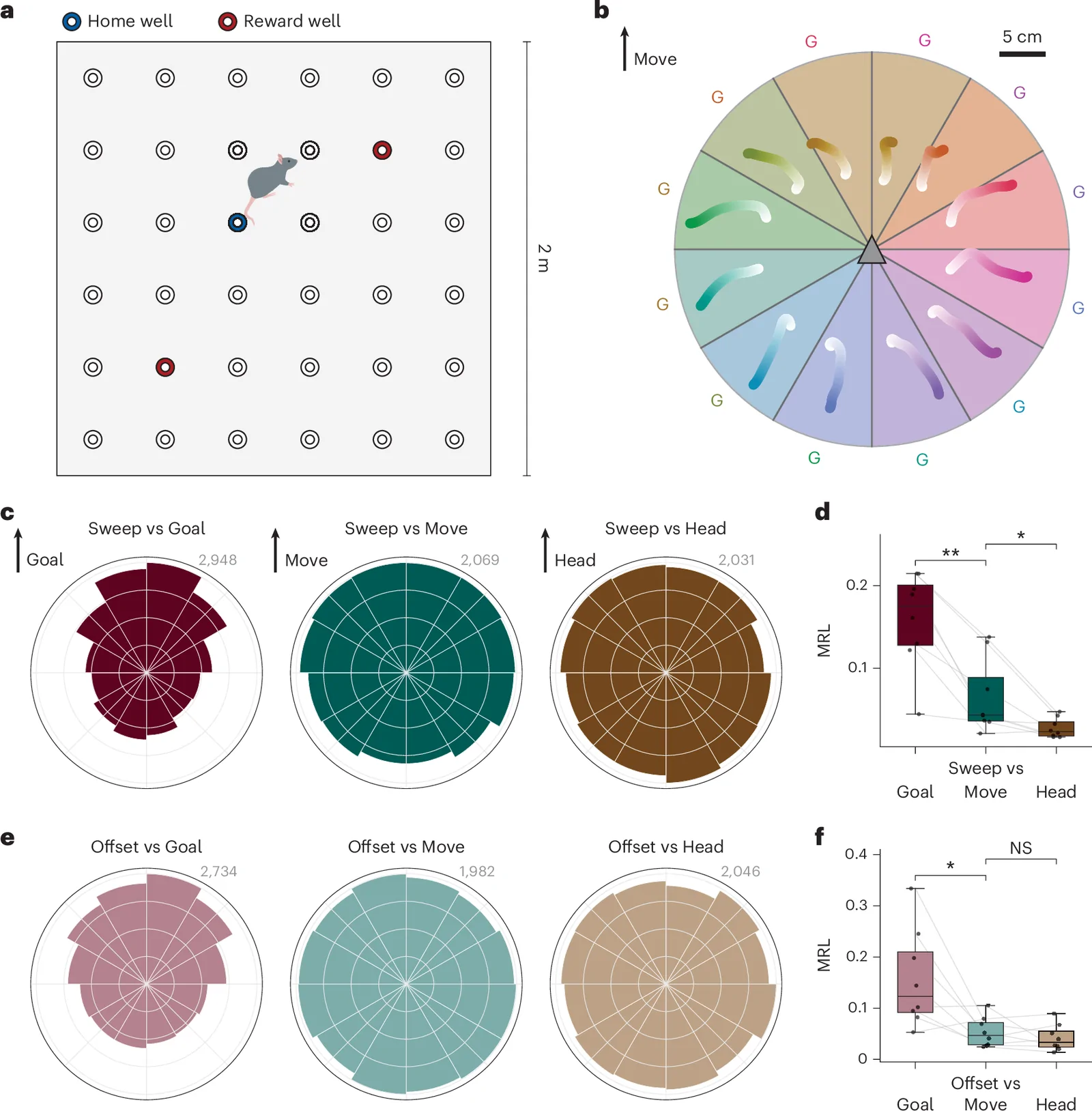
Theta sweeps exhibit task-contingent goal modulation in the Cheeseboard maze. **a**, Schematic of the Cheeseboard maze^20^. Rats performed foraging toward a randomly selected distant reward well (varying over trials) and goal-directed navigation toward a session-specific fixed home well on alternating trials. **b**, Median decoded theta sweep locations relative to an animal’s location (gray triangle) over homing trials, as a function of goal direction (colored ‘G’s) relative to movement direction (upward). **c**, Distributions of sweep direction relative to goal direction (left), movement direction (middle) and head direction (right), in all valid theta sweeps from homing trials in two recording sessions from each of the four recorded rats (one-sided shuffled test against chance level with *n* = 10,000 shuffles; Sweep versus Goal; *P* < 0.0001; Sweep versus Move: *P* < 0.0001, Sweep versus Head: *P* = 0.4390). **d**, Session-specific MRL of the distributions in **c** (two-sided paired *t*-tests with *n* = 8 sessions; Goal versus Move: *P* = 0.0030, *t* = 3.5791; Move versus Head: *P* = 0.0387, *t* = 2.2809; d.f. = 7). **e**, Same as **c** but with respect to the direction of the initial offset relative to the animal’s location (one-sided shuffled test with *n* = 10,000 shuffles: Offset versus Goal; *P* < 0.0001; Offset versus Move: *P* < 0.0001; Offset versus Head: *P* < 0.0001). **f**, Same as **d** but for distributions in **e** (two-sided paired *t*-tests with *n* = 8 sessions; Goal versus Move: *P* = 0.0113, *t* = 2.9171; Move versus Head: *P* = 0.4109; *t* = 0.8475; d.f. = 7). For box plots, center lines indicate medians, with box bounds indicating the interquartile range and whiskers indicating the minimum and maximum values. d.f., degrees of freedom; NS, not significant. Source data

**Extended Data Fig. 8:**
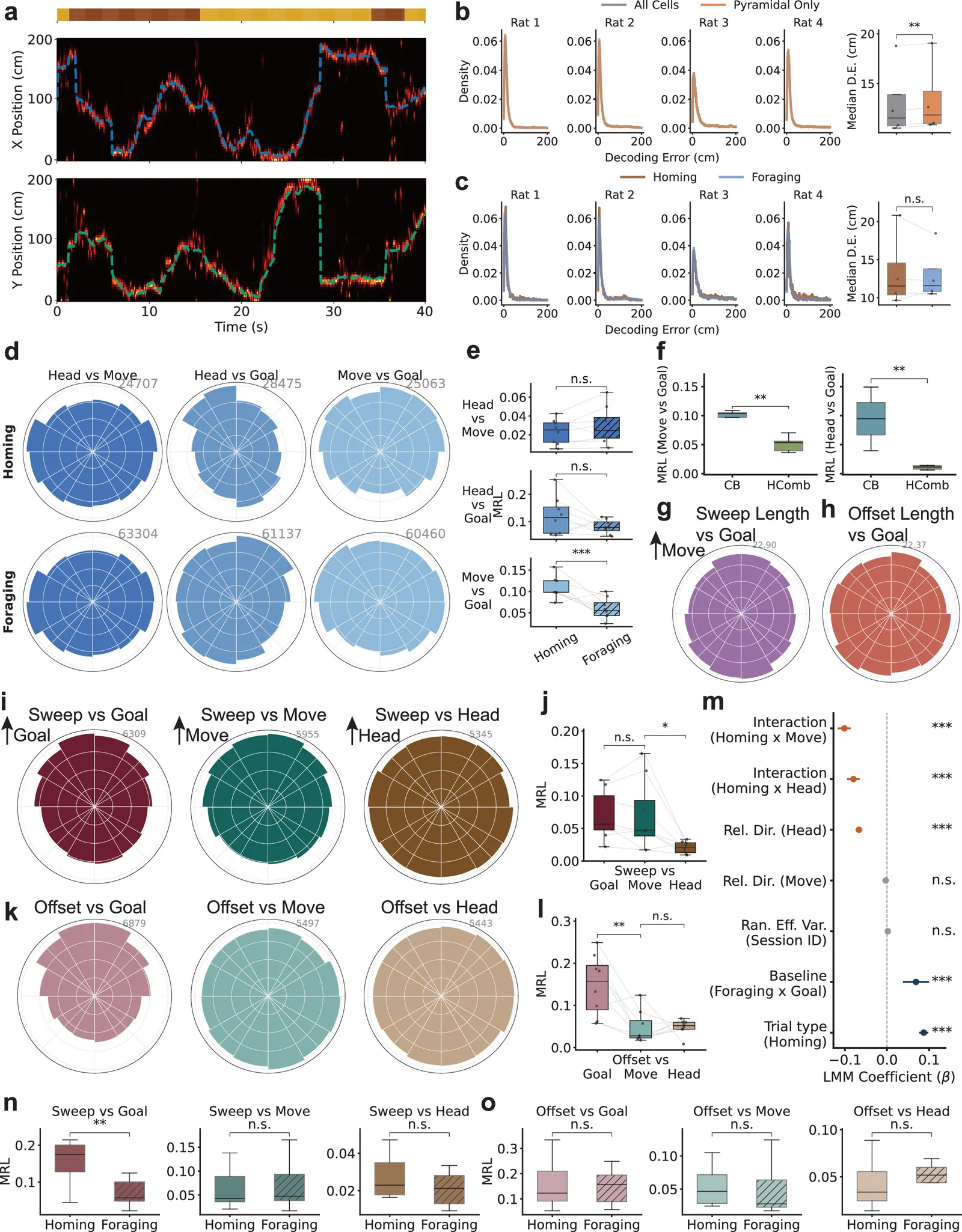
Theta sweeps exhibit goal-oriented directional bias in homing trials on the Cheeseboard maze, further details. **a**. Example Bayesian-decoding posterior probabilities over a held-out validation period lasting 40 seconds, plotted separately for the x- (top) and y- (bottom) positions. The presented probabilities are marginalised over the orthogonal dimension. Dashed lines represent the ground-truth x- (blue) and y- (green) locations of the rat. The horizontal bar above indicates trial identity (brown for homing; yellow for foraging). Qualitative inspection shows that Bayesian decoding yields accurate inference of the rat’s spatial position (median decoding error: 13.1050 ± 0.3490 cm, 4 rats). **b**. Probability of decoding errors given all cells and only putative pyramidal neurons for Rats 1–4 (from left to right). Rightmost panel: rat-specific median decoding error given Bayesian decoding using all cells and only pyramidal neurons (two-sided paired t-test: p = 0.0012, t = −10.5305; d.f.=3). **c**. Probability density of decoding errors over homing and foraging trials for Rats 1–4 (from left to right). Rightmost panel: rat-specific median decoding error over corresponding homing and foraging trials (two-sided paired t-test: p = 0.6622, t = 0.4705; d.f.=3). No significant difference in decoding accuracy is observed between homing and foraging trials. **d**. Circular histograms of relative angles between directions of (from left to right) heading and movement, heading and goal, movement and goal, over homing (top) and foraging (bottom) trials. Rats engaging in the Cheeseboard maze tasks exhibit strong alignment between head/movement direction and goal direction (cf. Extended Data Fig. 4b, c). Goal-movement alignment is stronger over homing trials compared to foraging trials. **e**. Session-level MRL of relative angles between head, movement, and goal directions, over homing and foraging trials in the Cheeseboard task (two-sided paired t-test, n = 8 sessions; Head vs Move: p = 0.7575, t = −0.7174; Head vs Move: p = 0.0775, t = 1.5029; Move vs Goal: p = 0.0008, t = 3.8906; d.f.=7). **f**. MRL of relative angles between goal direction and the animal’s movement (top) and head (bottom) directions during homing trials in the Cheeseboard (CB) maze and all trials over the Honeycomb (HComb) environment, showing greater alignment of movement and goal direction in the Cheeseboard maze (two-sided two-sample t-test, n_CB_ = 4 animals, n_HCoomb_ = 5 animals; Head vs Move: p = 0.0014, t = 5.1260; Head vs Goal: p = 0.5480, t = −0.6311; d.f.=8). **g**. Median sweep length as a function of relative movement direction (upward) to the goal. **h**. Same as **g**, but for the initial offset magnitudes of theta sweeps. **i**. Circular histograms of relative angles between directions of sweep and goal (left), sweep and movement (middle), and sweep and heading (right) over foraging trials. **j**. Session-specific MRL of distributions in **i** (two-sided paired t-tests, n = 8 sessions; Goal vs Move: p = 0.9752, t = 0.0316; Move vs Head: p = 0.0263, t = 2.4827; d.f.=7), **k**. Same as **i**, but for the direction of initial offsets of theta sweeps. **l**. Same as **j**, but for distributions in **k** (two-sided paired t-tests, n = 8 sessions: Goal vs Move: p = 0.0036, t = 3.4902; Move vs Head: p = 0.9621, t = −0.0484; d.f.=7). Sweep directions over foraging trials in the Cheeseboard maze exhibited similar levels of modulation by both goal and movement directions. In contrast, initial offset directions were exclusively modulated by goal directions and exhibited little movement-modulations. **m**. Linear mixed model coefficients (with confidence interval indicated by the error bar) predicting the cosine similarity of directions of individual theta sweeps (n = 85,210 valid theta sweeps) to goal, movement, and heading directions. The model includes random intercepts for session ID to account for the nested data structure (within-session variability). ‘Baseline’ represents the significant alignment between sweep and goal direction during foraging trials. ‘Homing’ trial type is the only additional factor inducing significant increase in goal-alignment of sweep directions (*β* = 0.0873, z = 15.9341, p = 3.6747 × 10^−57^). Error bars denote 95% confidence intervals for the coefficient estimates. Rel. Dir.: relative direction; Ran. Eff. Var.: random effect variance. **n**. Comparison of session-specific MRL of empirical distributions of relative sweep direction and goal, movement, and heading directions, over homing and foraging trials (two-sided two-sample t-test, n_homing_ = 8, n_foraging_ = 8 valid homing and foraging sessions, respectively, over 4 animals; Sweep vs Goal: p = 0.0259, t = 2.6133; Sweep vs Move: p = 0.4910, t = -0.7150; Sweep vs Head: p = 0.1197, t = 1.7016; d.f.=11). **o**. Same as **n**, but for the MRL of empirical distributions of relative angles with respect to initial offset directions (two-sided two-sample t-test; Offset vs Goal: p = 0.8479, t = 0.1954; Offset vs Move: p = 0.7473, t = 0.3287; Offset vs Head: p = 0.5573, t = -0.6012; d.f.=11). For boxplots, centre lines indicate medians, with box bounds indicate the interquartile range, and whiskers indicate the minimum and maximum values, excluding outliers. Source data

To validate our findings in settings with more recorded place cells (151.75 ± 59.25 putative place cells per rat, from four rats) but less separation among head, movement and goal directions (Extended Data Fig. 8d–f; compare to Extended Data Fig. 4b,c), we examined place cell recordings from the ‘Cheeseboard’ maze task^20^ (Fig. 4a). This task consists of trials of ‘foraging’ for randomly located rewards followed by ‘homing’, goal-oriented navigation toward a reward location, which remained constant for each session (see Pfeiffer and Foster^20^ for details). Theta sweeps were identified with similar criteria as described for the Honeycomb maze (minimum speed threshold increased to 5 cm s^−1^ as rats run faster on this maze), leading to a total of 85,210 theta sweeps over two recording sessions (rat 1: 6,180, 13,985; rat 2: 10,395, 12,299; rat 3: 12,375, 11,814; rat 4: 9,772, 8,410).

Compared to the Honeycomb maze, movement directions, but not head directions, were more frequently aligned with goal directions on the Cheeseboard maze (Extended Data Fig. 8d–f; compare to Extended Data Fig. 4b,c). During homing trials, we observed similar goal-oriented directional bias in both sweep directions and initial offset directions as in the Honeycomb maze (Fig. 4b–f). As expected, sweep directions exhibited significantly weaker goal bias during foraging trials compared to homing trials but similar movement biases (Extended Data Fig. 8i,j,m,n). By contrast, the goal-directed bias of sweep initial offsets did not differ between foraging and homing trials (Extended Data Fig. 8k,l,o).

To investigate whether goal bias in theta sweep directions is dependent on task demand at the level of the individual sweep, we applied the LMM analysis described above, now with trial type as an additional factor (categorical predictors: homing and foraging) as well as interaction terms between trial types and reference directions (Extended Data Fig. 8m). We found that sweeps were significantly goal directed even during foraging trials (baseline intercept: *β* = 0.0694, *z* = 4.3561, *P* = 0.0002); however, the effect of movement alignment was statistically indistinguishable from goal alignment (*β* = −0.0033, *z* = −0.7635, *P* = 0.4460), suggesting that sweep directions reflect the coupling between movement and goal directions during random foraging. Goal-directed navigation during homing trials significantly strengthened the alignment between sweep and goal directions (effect of homing trial type: *β* = 0.0872, *z* = 15.9349, *P* < 0.0001), effectively doubling the magnitude of the goal bias during foraging. Notably, although sweeps tracked both movement and goal directions equally during foraging, sweeps were selectively decoupled from movement direction during homing trials, evidenced by the negative interaction between homing and movement direction (*β* = −0.1018, *z* = −13.0906, *P* < 0.0001). Finally, we modeled individual recording sessions (each characterized by a distinct goal location during homing trials) rather than rat identity as random effects. We found no modulatory effect on alignment between sweep and corresponding goal directions arising from session-specific reward locations (session ID: *β* = −0.0017, *z* = 1.2146, *P* = 0.2240), similarly to the sweep bias toward the current rather than previous goal location after reward-switch trials on the Honeycomb maze.

In summary, theta sweeps during homing trials on the Cheeseboard maze^20^ showed a robust goal-directedness, similarly to those on the Honeycomb maze^19^. During foraging trials, theta sweeps had a weaker goal direction bias, of similar strength to the movement direction bias (as expected by the significant relationship between both directions in this task).

### CAN with theta rhythmicity, firing rate adaptation and directional goal modulation reproduces theta sweep characteristics

**Fig. 5:**
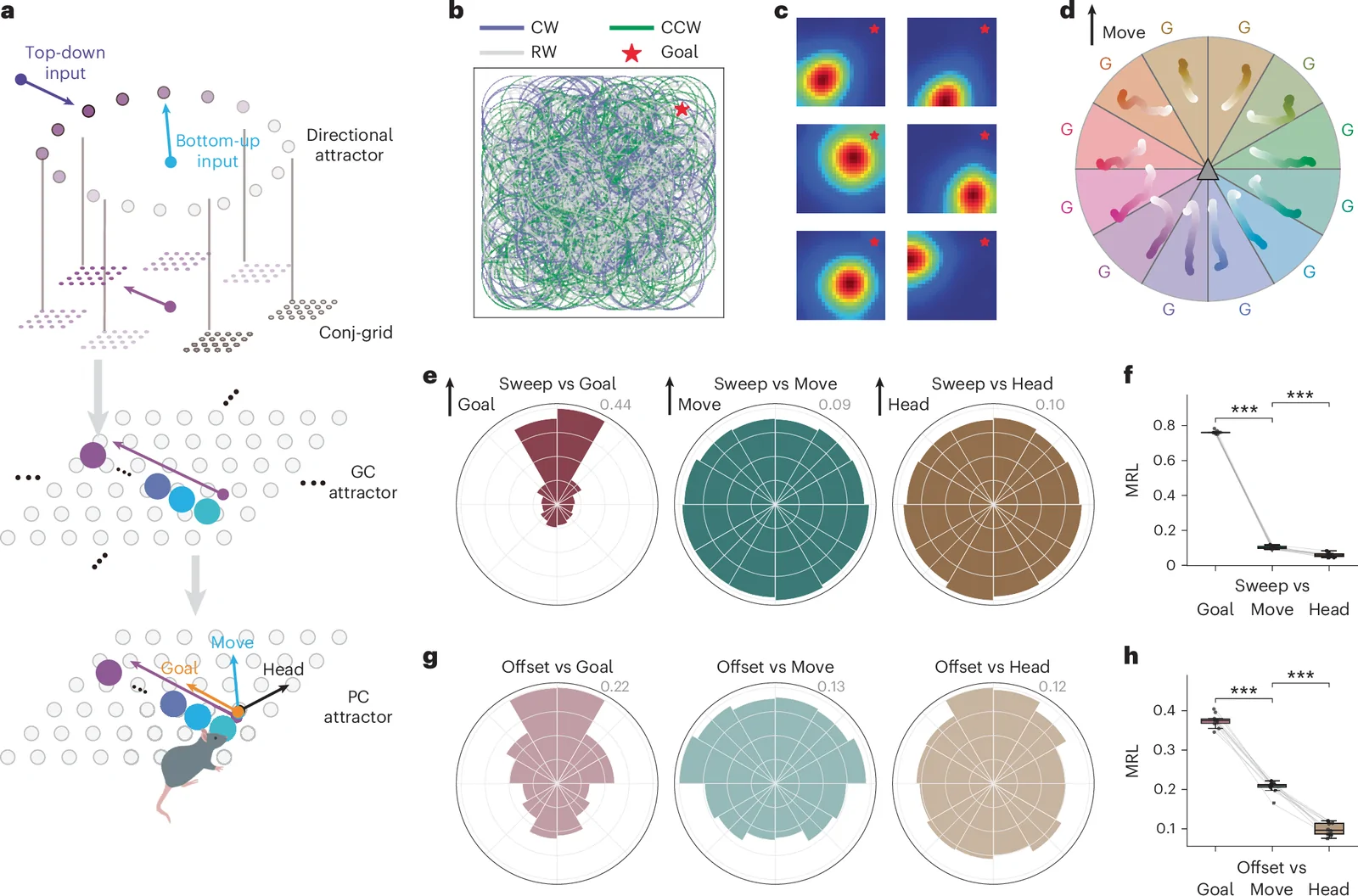
A CAN with firing rate adaptation and goal direction input captures goal-oriented theta sweeps. **a**, Schematic illustration of the hierarchical model. The highest-level directional cell (DC) ring attractor network receives input of the animal’s movement direction and a top-down allocentric directional input toward the goal location. Conceptually, the DC attractor projects directional input with goal-oriented bias to the conjunctive grid cells (GCs)^52^ which shifts the activity bump in the downstream GC attractor network along the goal direction. The GC attractor has a one-to-one mapping onto the hippocampal place cell (PC) attractor, driving the PC activity bump toward the goal direction. The presented schematic illustrates the conceptual hierarchical computations underlying the model, while our simulations used a simplified version with explicitly implemented DC and PC networks and equivalent computations that implicitly capture the influence of intermediate conjunctive and standard GC layers (see main text and Methods). Orange, cyan and black arrows indicate the goal, movement and head directions, respectively. **b**, Sample trajectory used in simulation, consists of random walks (RWs) and significant periods of rotational ‘scanning’ movements (both clockwise (CW) and counterclockwise (CCW)). **c**, Example spatial tuning of simulated PCs. **d**, Median simulated theta sweep locations as a function of goal direction (colored ‘G’s) relative to movement direction. **e**, Circular histograms of relative directions between sweep and goal (left), movement (middle) and head (right) directions in simulated theta sweeps. **f**, MRL of distributions in **e** (10 random seeds; two-sided paired *t*-tests; Goal versus Move: *P* = $4.7617\times {10}^{-30}$, *t* = 164.3844; Move versus Head: *P* = $1.9586\times {10}^{-8}$, *t* = 9.4988; d.f. = 9). **g**, Same as **e** but for relative angles with respect to the initial offset direction. **h**, Same as **f** but for distributions in **g** (two-sided paired *t*-tests; Goal versus Move: *P* = $2.1349\times {10}^{-15}$, *t* = 23.9281; Move versus Head: *P* = $5.1367\times {10}^{-12}$, *t* = 15.2046; d.f. = 9). For box plots, center lines indicate medians, with box bounds indicating the interquartile range and whiskers indicating the minimum and maximum values, excluding outliers. Conj-grid, conjunctive grid; d.f., degrees of freedom. Source data

Forward theta sweeps in place cells and their characteristic left−right alternation during open-field foraging are driven by theta-modulated parasubicular directional cells, mediated by conjunctive directional grid cells and classic grid cells in entorhinal cortex^9^. The emergence of these left−right alternating theta sweeps can be modeled by simulating these neural populations and including CAN dynamics, location-specific inputs, speed-dependent theta modulation and firing rate adaptation in the directional and grid populations. In this framework, intrinsic dynamics in the directional cell network shapes the direction of activity propagation in the grid cell attractor and yields sweep-like trajectories within each theta cycle. To extend this account to hippocampal goal-directed theta sweeps, we proposed a hierarchical extension (Fig. 5a), in which an additional goal-oriented allocentric directional input biases directional cell activities toward the goal direction. Conceptually, this bias would propagate through the grid cell circuitry to drive goal-directed sequential activation in a downstream two-dimensional place cell attractor network. Here, we explicitly simulate the directional cell and place cell populations as CANs with location-specific inputs, theta modulation and firing rate adaptation while implicitly including the intermediate grid cell computations in the coupling from directional cells to place cells (see further details in Methods).

**Extended Data Fig. 9:**
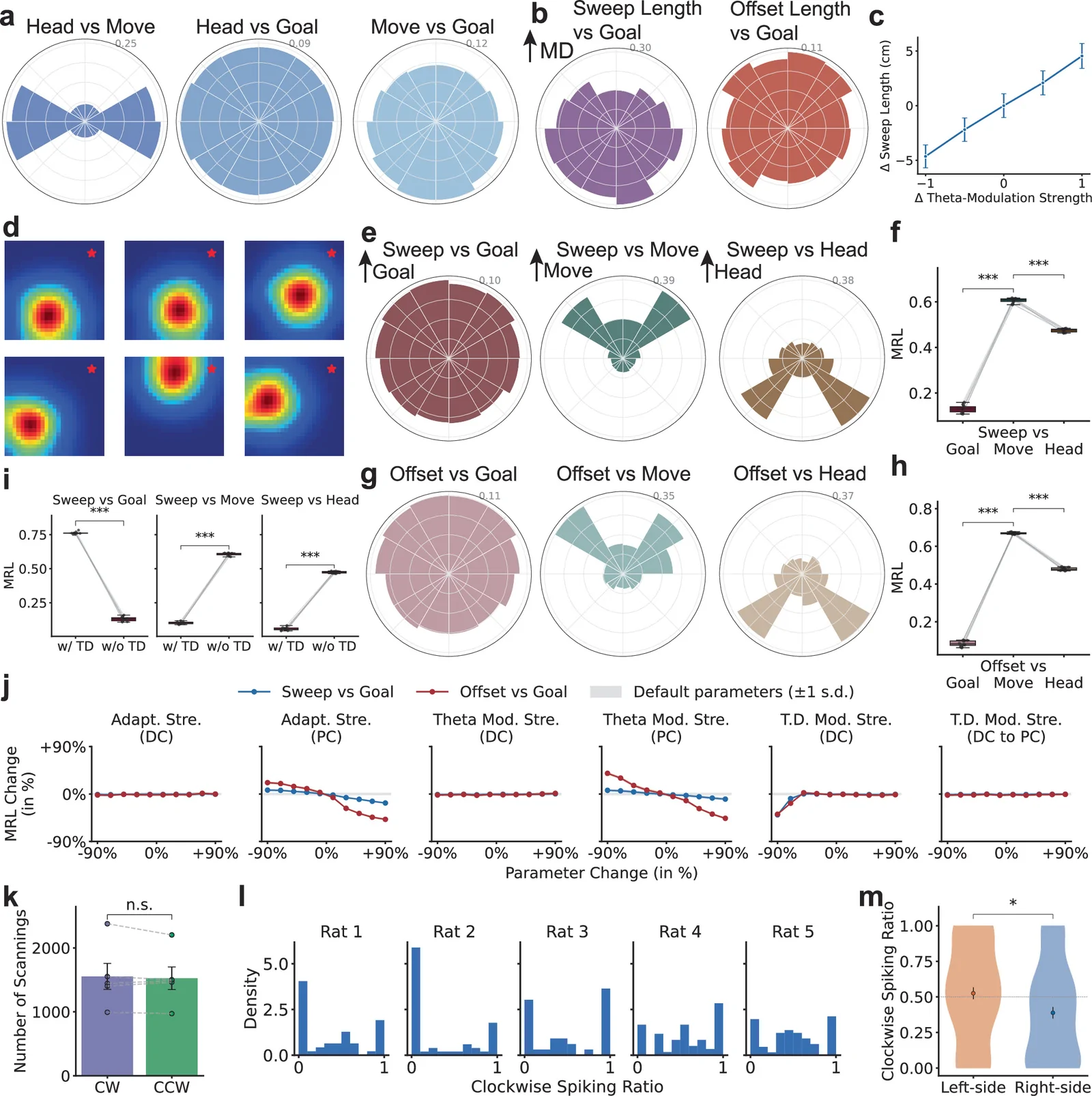
Absence of goal-directed theta sweeps after turning off the top-down goal-directed input in the hierarchical CAN model. **a**. Distributions of relative angles between head, movement, and goal directions over simulated trajectories (Fig. 5c). Same behavioural trajectories were used in both simulations with and without top-down inputs. The frequent occurrence of orthogonal head and movement directions was due to the simulated rotational scanning (where movement was always perfectly sideways to the heading, see Methods). Analogous to real data in the Honeycomb maze, neither headings nor movement exhibited goalward biases. **b**. Median sweep length (left) and initial offset length (right) given varying relative movement direction (upward) to the goal (cf. Figure 2a). **c**. Mean sweep length (± 1 s.e., n = 137,651 theta sweeps) as a function of increasing theta-modulation strengths given simulated theta sweeps (cf. Figure 2c, left panel). Note that results in panels **b** and **c** were simulated using our main model (Fig. 5a). **d**. We performed ablation studies by turning off the top-down input. This effectively yielded sweeps in the directional cell network that proceed along the internal directions, instead of the goal direction, resulting in left-right alternating sweeps along the movement direction in the activity bump in the downstream place cell network^9,21^. Example spatial tuning of selected neurons in the place cell network exhibits similar unimodal spatial selectivity when the top-down goal-oriented directional input is turned off. **e**. Distributions of relative angles between sweep direction and goal (left), movement (middle), and head (right) directions. **f**. MRLs of distributions in **e** (two-sided paired t-test, n = 10 random seeds; Goal vs Move: p = $2.5562\times {10}^{-28}$, t = −131.7294; Move vs Head: p = $8.0319\times {10}^{-26}$, t = 95.6657; d.f.=9). **g**. Same as **e**, but for distributions with respect to initial offset directions. **h**. Same as **f**, but for distributions in **g** (two-sided paired t-tests, n = 10 random seeds; Goal vs Move: p = $2.1677\times {10}^{-22}$, t = -61.5977; Move vs Head: p = $1.5801\times {10}^{-18}$, t = 37.4382; d.f.=9). **i**. Comparison of MRLs of distributions of relative angles between sweep direction and goal (left), movement (middle), and head (right) directions, when the top-down input to directional cells was turned on and off, respectively (two-sided paired t-test, n = 10 random seeds; Sweep vs Goal: p = $2.0694\times {10}^{-28}$, t = 133.2859; Sweep vs Move: p = $7.5782\times {10}^{-31}$, t = −182.0666; Sweep vs Head: p = $1.6067\times {10}^{-22}$, t = −62.6357). As predicted, the top-down input is an essential mechanism underlying goal-directed theta sweeps in our simulations, and simulated theta sweeps predominantly following movement direction and exhibit little goal-modulation in its complete absence. **j**. Changes in MRL (mean ± 1 s.d. over 10 random seeds) of distributions of relative angles between sweep and goal directions (blue), and initial offset and goal directions (red), as a function of varying values of adaptation strength in directional cells (top left), adaptation strength in place cells (top middle), theta modulation strength in directional cells (top right), theta modulation strength in place cells (bottom left), strength of top-down goal-oriented input to directional cells (bottom middle), and projection strength from directional cells to place cells (bottom right). All presented sensitivity analysis were uni-dimensional by fixing all other parameters in the model as default (Supplementary Table 1). All statistics were based on 10 independent simulations. **k**. Number of clockwise and counterclockwise active scanning periods of each rat (with bar heights and error bars indicate mean and s.e. over rats; two-sided paired t-test, n = 5 animals: p = 0.5348, t = 0.6782, d.f.=4). **l**. Histograms of ratio of cell-specific spikings during clockwise scanning (#spikes over CW scanning/#total spikes during active scanning) for all rats. **m**. Clockwise spiking ratio (with centre dot and error bar indicate mean and s.e. over cells) for cells with firing fields lying on the left- and right-side of the vector connecting the closest platform centre and the goal location (two-sided Mann-Whitney U test: p = 0.0138, t = 3915.0000, n_left_ = 79, n_right_ = 81). For boxplots, centre lines indicate medians, with box bounds indicate the interquartile range, and whiskers indicate the minimum and maximum values, excluding outliers. Source data

Applying our model to simulated trajectories resembling those of rats in the Honeycomb maze (interleaved random walks and rotational scanning periods; Fig. 5c and Methods), the place cell activity successfully reproduced our key empirical findings. Decoded theta sweeps, and their initial offsets, exhibited strong goal-oriented directional bias and significantly weaker modulatory effects from movement or head direction (Fig. 5f,g). By contrast, in the absence of top-down goal-directed inputs, the resultant model is equivalent to the base model^21^, yielding theta sweeps with left−right alternation about the movement direction (Fig. 5b and Extended Data Fig. 9d–i). To ensure the robustness of our findings, we performed a sensitivity analysis by independently varying key model parameters (Extended Data Fig. 9j). Goal-directed modulation remained robust across a wide range of parameter values. Simulated sweeps also extend further toward the goal during movement opposite to the goal direction (Extended Data Fig. 9b), consistent with the data. Thus, the model reproduces both forward sweeps with left−right alternation during foraging and goal-directed sweeps when a top-down goal-directed input is applied, as expected.

The model also reproduces more subtle aspects of the data. In addition to the dynamical input from the grid system, theta modulation and firing rate adaptation in the place cells contribute to the theta sweeps, with increasing theta modulation causing longer sweeps^10,21^, reproducing the positive scaling of sweep lengths with theta power (Extended Data Fig. 9c; compare to Fig. 2c). The effect of firing rate adaptation also interacts with movement direction in interesting ways. Place cells with firing fields behind the rat will have fired more recently than those with fields in front of it and, therefore, exhibit greater firing rate adaptation. This biases place cell activity in the forward direction from the ratʼs location, causing the initial offset of goal-directed theta sweeps in the forward movement direction, as seen in the data (compare to Figs. 1f, 2a and 5d and Extended Data Fig. 9b).

**Fig. 6:**
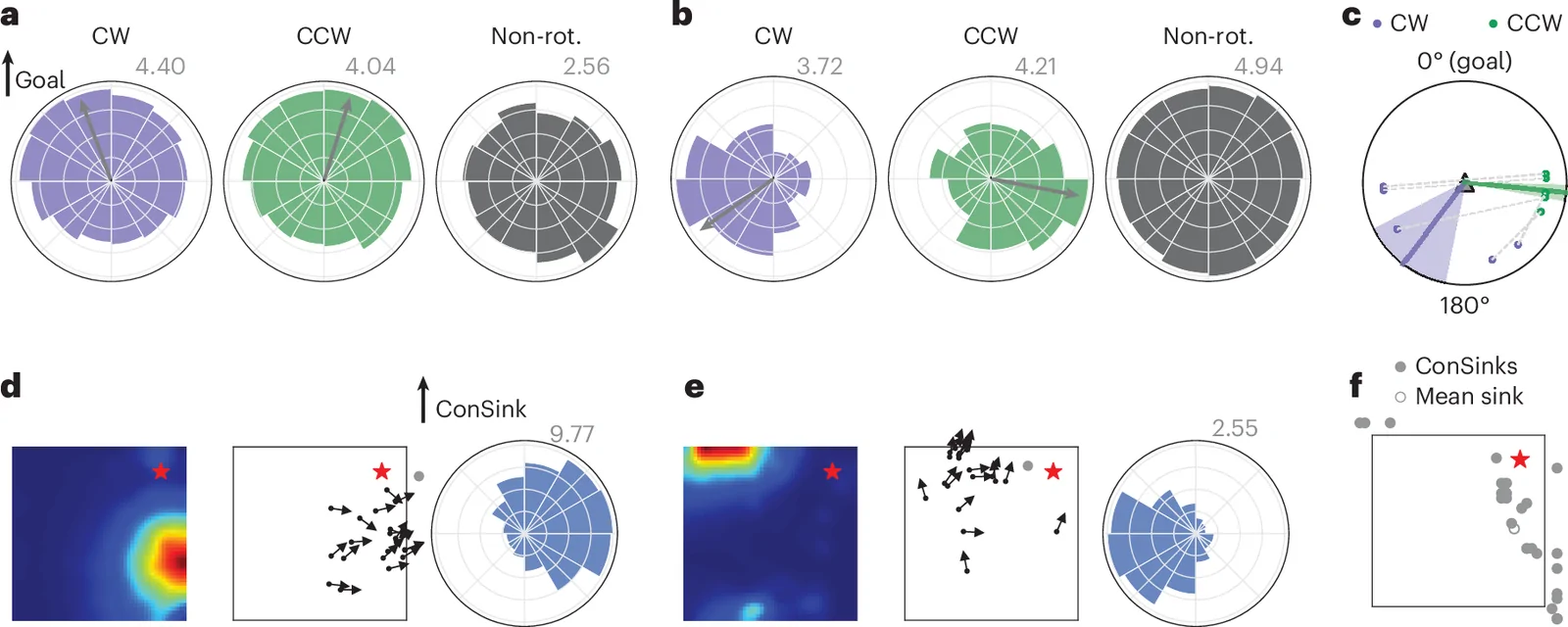
Goal-oriented anticipatory coding predicts goal-directed egocentric directional tuning in place cells. **a**, Average firing rate (over all simulated place cells) as a function of relative movement direction to the goal (upwards) during clockwise (CW) and counterclockwise (CCW) continuous rotational scanning periods (Methods) and all remaining periods (right). Gray arrow indicates expected relative direction of spiking. **b**, Same as **a** but for real data from all place cells on the Honeycomb maze. **c**, Rat-specific expected relative directions of spiking (dots, with s.e. over rats indicated by shaded areas) during CW and CCW rotational scanning. Gray dashed lines connect expected relative directions from the same rats. **d**, An exemplar ‘ConSink’ cell^19^ from the simulated model (from left to right): spatial rate maps; locations and head directions during spiking of the selected neuron and associated ConSink location (gray dot; MRL of head directions relative to the ConSink location during spiking is 0.2154); firing rate map as a function of head direction relative to the ConSink direction (upwards). Red star indicates the goal location. **e**, Same as **d** for a different ConSink cell. **f**, ConSink locations of all simulated neurons with significant egocentric ConSink-oriented directional tuning. Non-rot., non-rotational. Source data

The interaction between firing rate adaptation and movement direction also causes greater firing during goal-directed sweeps to goals ahead of the movement direction, because they involve place cells that have not fired recently. This predicts modulation of firing rate by alignment between movement and goal direction, which we verified in the data (Extended Data Fig. 4i). Thus, during rotational scanning behavior, place cells should fire more before the head direction reaches the goal direction (when the goal is ahead of the rotational movement) than after it passes the goal direction (Fig. 6a). We verified the presence of this rotational goal-anticipatory firing in the experimental data (Fig. 6b,c). The modulation of firing rates by the angle between goal and movement directions (greater firing when they align) also predicts a modulation of the firing rates of individual place cells by the angle between head and goal directions. This tuning will tend to be peaked when goal and head direction align but can peak at other angles according to the precise sampling of movement and head directions in the corresponding place fields (for example, 90° if predominantly sampled during clockwise or counterclockwise rotational scanning). This potential mechanism could contribute to the observation that place cells in the Honeycomb maze show a convergent egocentric directional bias toward specific locations (convergence sinks (ConSinks)), which cluster around the goal location^19^. Indeed, without any parameter tuning, many simulated place cells exhibited ConSink-like egocentric directional tuning (Fig. 6d,e), with ConSinks that cluster around the goal location (Fig. 6f).

### Goal-oriented theta phase coding

**Fig. 7:**
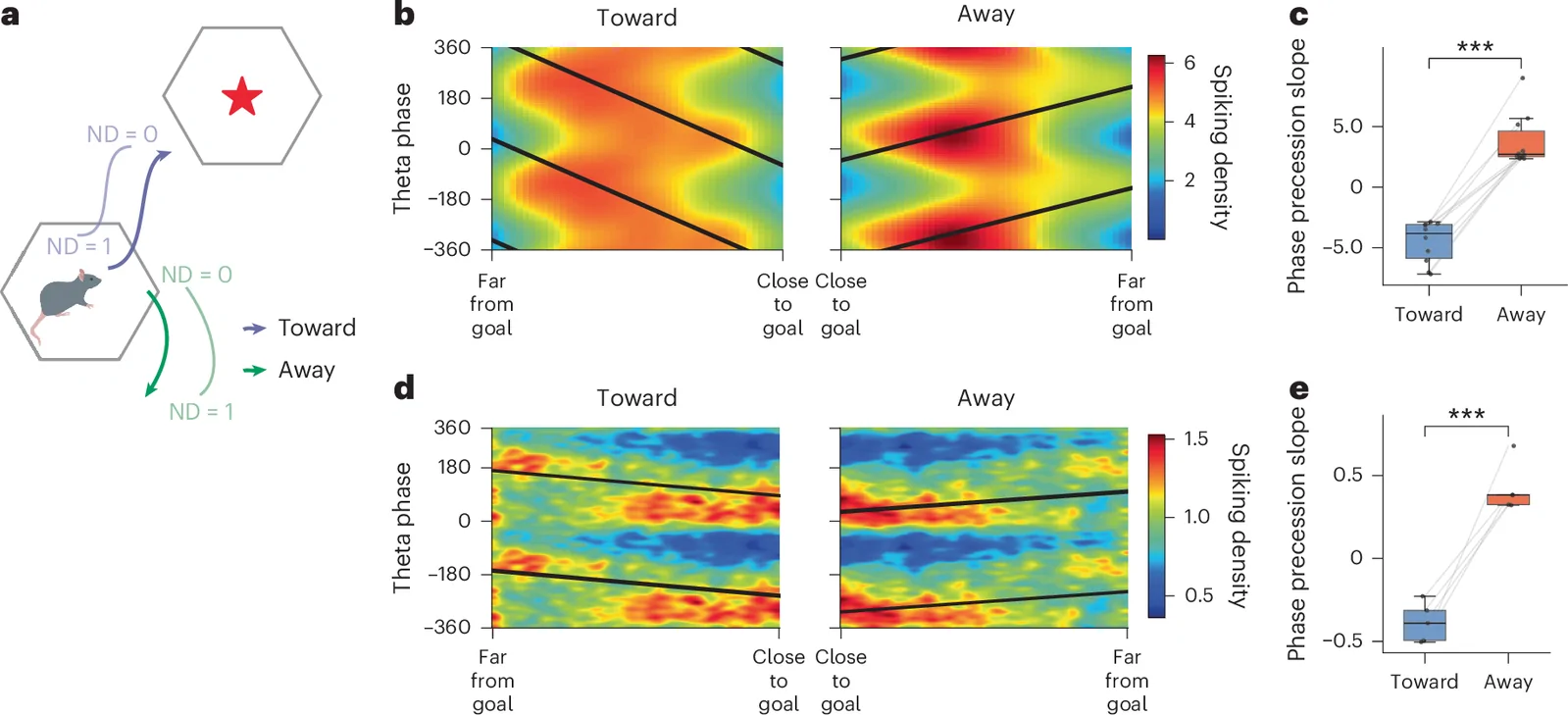
Goal-dependent phase coding as a single-cell consequence of goal-directed theta sweeps. **a**, Example trajectories toward and away from the goal (star platform), illustrating the normalized distance (ND) to the goal (Methods). **b**, Theta phase of model-generated Poisson spikes as a function of ND during continuous rotational scanning (Fig. 5b) toward and away from the goal. Black lines represent (wrapped) best-fit circular−linear regression line between ND and theta phase. *P* values were obtained using Rayleigh circular−linear tests^53^, which assess non-uniform phase concentration around the fitted circular−linear relationship using an upper-tailed test (toward: *r* = −0.0518, *P* < 10^−308^; away: *r* = 0.0448, *P* < 10^−308^). **c**, Circular−linear slopes between theta phase and ND during continuous trajectories toward and away from the goal, respectively, given network-simulated spikes (two-sided two-sample *t*-tests, *n* = 10 random seeds: *P*= $2.0136\times {10}^{-8}$, *t* = −9.4815, d.f. = 18). **d**, Same as **b** but with real spiking from all place cells during continuous movement periods toward and away from the goal (toward: *r* = −0.0264, *P* < 10^−308^; away: *r* = 0.0070, *P* = 0.0184). **e**, Rat-specific circular−linear slopes between theta phase and ND for toward and away trajectories, respectively (two-sided paired *t*-test, *n* = 5 animals: *P* = $4.2622\times {10}^{-5}$, *t* = −8.0312, d.f. = 4). For box plots, center lines indicate medians, with box bounds indicating the interquartile range and whiskers indicating the minimum and maximum values, excluding outliers. d.f., degrees of freedom. Source data

In rodents running on linear tracks or open fields, place cell firing exhibits ‘theta phase precession’, in which place cells fire at late phases of the theta rhythm when their firing field is ahead of the animal and at successively earlier phases as the animal moves through the field^22^, consistent with a higher intrinsic firing frequency than the LFP theta^22^. Thus, population theta sweeps are thought to be consistent with single-cell theta phase precession (that is, place cells active at early phases having firing fields behind the rat and those active at later phases having firing fields ahead of the rat)^2,3^, with the additional requirements of brief experience of the environment^28^ and CA3 activity^29^. However, the goal-directed theta sweeps presented here predict theta phase ‘precession’ when the animal is moving toward the goal and theta phase ‘procession’ when the animal moves away from the goal (Fig. 7a–c). This is because, during sweeps toward a goal located behind the rat, place cells active at early phases have firing fields ahead of the rat, and those active at later phases have firing fields behind the rat and closer to the goal. We confirmed this prediction, finding theta phase precession and procession with respect to distance traveled toward or away from the goal, respectively (Fig. 7d,e). Thus, in this situation, in which theta sweeps do not simply propagate forward, single-cell phase coding appears to reflect the population dynamics rather than vice versa.

### Goal-oriented hippocampal replay sequences

Another form of sequential neural representation in place cells^20,23^ occurs during immobility (either awake or asleep), associated with sharp-wave ripples^1^ instead of theta rhythmicity^22^, often referred to as ‘replay’. The decoded trajectories in open-field arenas often exhibit a goal-directed bias^15,20^, require LTP during encoding^30^ and contribute to memory consolidation^31,32^. These trajectories are generally thought to reflect recent experiences^33^, including those leading to reward consumption^34^, hence the term ‘replay’. However, the fact that replay is contingent on theta rhythmicity during encoding^25,35^ and includes locations that have not been physically visited^36^, combined with the way theta sequences afford plasticity between place cells with adjacent firing fields^22,37^, suggests that replay trajectories might be shaped by the trajectories played out during theta sweeps rather than the trajectories of locations that have been actually visited by the rat^15,24^.

**Fig. 8:**
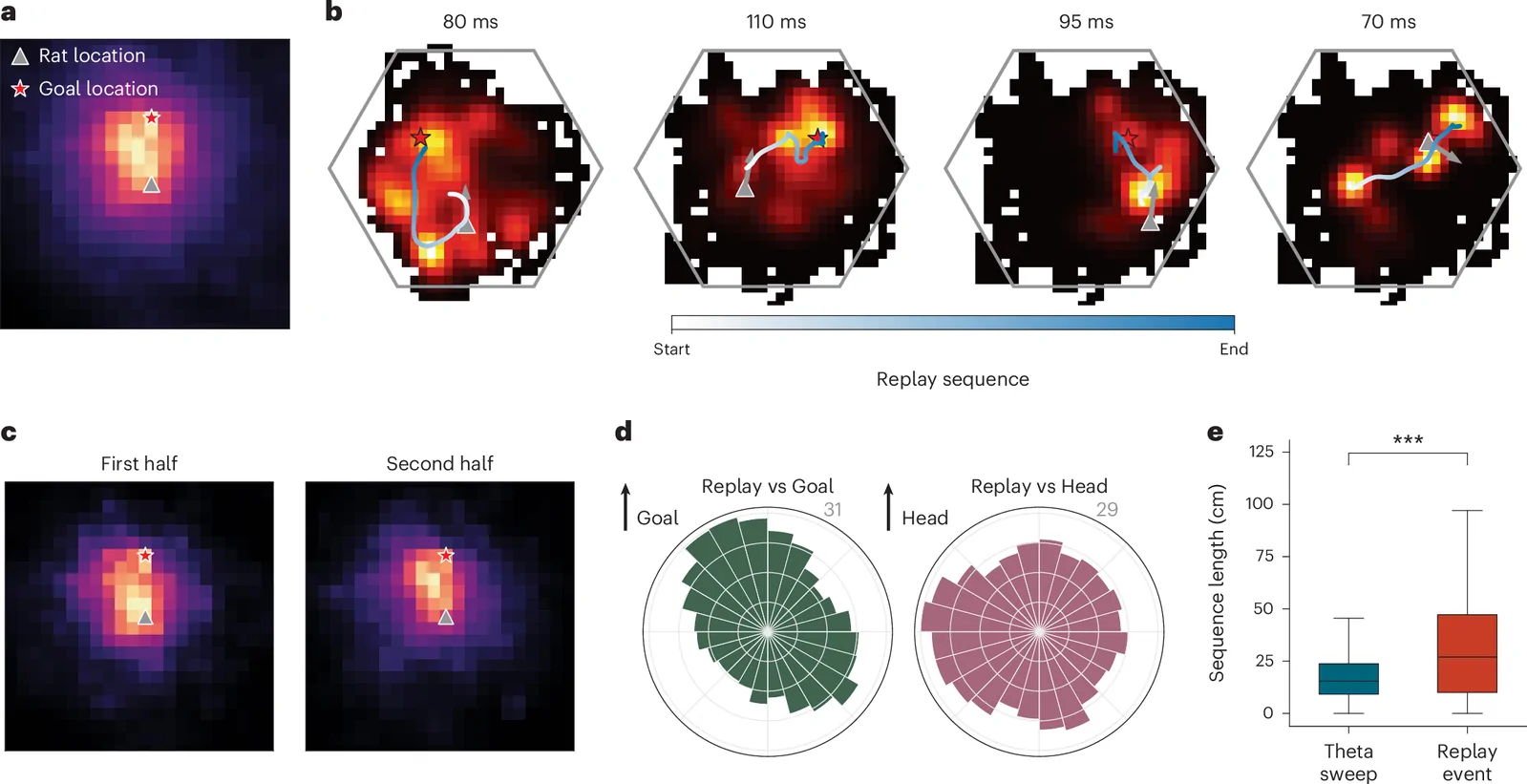
Goal-oriented replay sequences in the absence of direct path. **a**, Average decoded posterior probability of all replay events, standardized with respect to the rat location and direction to goal and scaled according to the distance to the goal location. **b**, Example replay trajectories (from light to dark blue) with summed posterior probability. Gray arrowhead denotes the rat’s head direction; the duration of the replay event is shown above. **c**, Average decoded posteriors (standardized and scaled) during the first (left) and second (right) half over all candidate replay events. **d**, Distributions of direction of replay sequences (Methods), relative to goal (left) and head (right) direction, upwards in each plot (one-sided shuffled test against chance level with *n* = 10,000 shuffles: Replay versus Goal: *P* < 0.0001; Replay versus Head: *P* = 0.4977). **e**, Distributions of lengths of theta sweeps and replay sequences (pooled over all five rats; two-sided Mann−Whitney *U*-test: *U* = 56,414,443.0, *P* < 10^−308^; effect size *r* = 0.2958; *N*_sweep_ = 137,651, *N*_replay_ = 537). For box plots, center lines indicate medians, with box bounds indicating the interquartile range and whiskers indicating the minimum and maximum values. Source data

**Extended Data Fig. 10:**
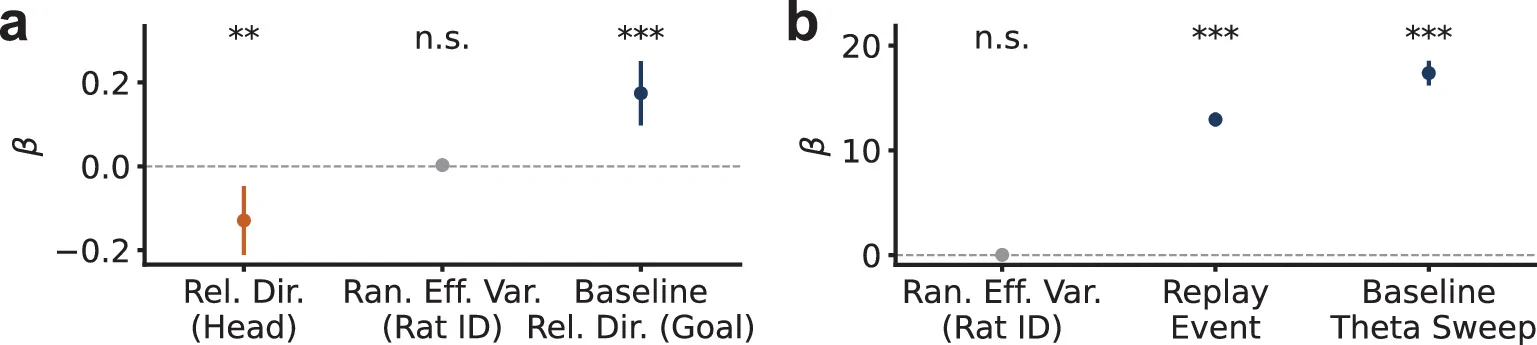
LMM analysis of replay direction and length. **a**. Linear Mixed Model coefficients for predicting cosine similarity between replay and reference relative directions (n = 537 valid replay sequences), based on fixed-effect predictors (categorical reference relative direction type: goal/head directions) whilst accounting for within-animal variability (rat-level random intercepts). Baseline intercept (relative goal direction): *β* = 0.1742, z = 4.4478, p = 8.6758 × 10^−6^; relative movement direction: *β* = −0.1294, z = −3.0878, p = 0.0020; random effect variance: *β* = 0.0027, z = 0.4679, p = 0.6398. **b**. LMM coefficients for predicting sequence lengths (n_sweep_=137,651, n_replay_ = 537 valid theta sweeps and replay sequences, respectively), based on fixed-effect predictors (categorical sequence type: theta sweep and replay event) whilst accounting for within-animal variability. Baseline intercept (theta sweep): *β* = 17.3682, z = 29.0586, p = 1.1976 × 10^−185^; fixed effect (replay event): *β* = 12.9355, z = 38.2558, p < 10^−308^; random effect variance: *β* = 0.0135, z = 1.4019, p = 0.1609. Rel. Dir.: relative direction; Ran. Eff. Var.: random effect variance. LMM analysis are reported in forest plots showing fixed-effect coefficients (with 95% confidence interval indicated by the error bar).

We examined periods of awake immobility on the Honeycomb maze (speed <2 cm s^−1^, *z*-scored MUA >3) and identified candidate replay events (rat at least 50 cm from the goal; in total, 537 events over five rats; Methods). For aggregated analysis, we rotated and scaled each decoded replay event such that the goal location is always at a fixed distance north of the rat’s location^20^. The decoded posterior probabilities overrepresented locations between the rat and the goal, with decoded locations closer to the goal in the second half of each event than in the first half (Fig. 8a,c). Visual examination of individual replay trajectories corroborated the aggregated analysis (Fig. 8b). Replay trajectories preferentially aligned with the goal direction (Fig. 8d). To account for intra-animal variability, we again performed the corresponding LMM analysis and found that replay directions are more strongly aligned with goal directions than head directions (Extended Data Fig. 10a). Replay sequences were significantly longer than theta sweeps (Fig. 8e and Extended Data Fig. 10b). As noted above, active movements on the Honeycomb maze did not exhibit oversampling of goal directions (Extended Data Fig. 4b). Taken together, our results suggest that goal-directed ‘replay’ sequences are not simple recapitulations of recently experienced trajectories. Instead, they represent trajectories to the goal perhaps shaped by plasticity between place cells with adjacent fields that are activated during theta sweeps^24^, effectively linking multiple theta sweeps into longer replay trajectories^15,24^.

## Discussion

Our results revealed robust directional modulation of hippocampal theta sweeps toward remembered goal locations rather than sampling potential movement directions^4,6,7,38^ or perceptual targets^13^. Although previous studies showed that theta sweeps can overrepresent salient locations on narrow-armed mazes, such as aversive zones^11,12^, we demonstrate that they represent a true memory-guided goal direction vector, decoupled from the animal’s movement, heading or perception. The goal direction bias is stronger before correct navigational choices and increases with experience, complementing previously observed correlations with learning^15^. Our findings are consistent across navigation tasks in both the Honeycomb maze^19^, which has strong behavioral constraints, and the open-field Cheeseboard maze^20^, in which the goal-directedness of theta sweeps is task dependent^13^, occurring more on homing than foraging trials.

Our findings suggest that theta sweeps may implement a key element of the cognitive map^1,39^, encoding the goalward vector for planning. These vectors may represent a pure cognitive goal vector encoding the direction and distance to remembered goal locations irrespective of immediate sensory or movement constraints and potentially support flexible online planning strategies.

Left−right and forward sweeps during foraging are driven by parasubicular theta-modulated directional cells via grid cells^9^, and we assume that the same circuit drives goal-directed sweeps. Building upon an existing CAN model that captures forward and left−right alternating theta sweeps in parasubicular−entorhinal circuitry^21^, we proposed a hierarchical extension to include place cells and an additional goal-directed top-down input to directional cells. Our model successfully reproduced key characteristics of goal-directed theta sequences without requiring task-specific training or parameter optimization. Notably, the additional goal-oriented input does not merely impose a goalward firing rate bias; it modulates population dynamics while preserving constraints from sensorimotor input, theta modulation and firing rate adaptation. This leads to numerous nontrivial predictions, including rotational goal-anticipatory firing, a reversal between theta phase precession and procession depending on alignment between goal and movement directions and the emergence of goal-directed egocentric directional tuning in individual place cells^19^. The proposed mechanism unifies goal-directed coding and theta phase coding on both population and single-cell levels and identifies circuit-level variables whose causal contributions can be tested in future experiments.

Our modeling assumptions raise the question of the source of the goal direction signal. One candidate would be retrosplenial cortex, which encodes behaviorally salient locations in both allocentric and egocentric reference frames^40,41^ and potentially receives self-motion and goal information from posterior parietal cortical neurons^42,43^. An alternative would be medial prefrontal cortex, consistent with hippocampal−prefrontal theta phase coupling associated with learning at choice points^44,45^.

We found that ‘replay’ sequences during immobility on the Honeycomb maze, in which no direct goalward trajectories physically occurred, were also goal directed, like the theta sweeps. This supports the idea that these trajectories are formed by plasticity between place cells activated during theta sweeps^3,15,25^, consistent with hypotheses that they are concatenations of theta sweep segments^24,30,35^. Thus, offline replay events may consolidate desired trajectories encoded by theta sweeps during active locomotion into long-term spatial knowledge, potentially supporting subsequent planning^36^.

The conservation of theta rhythms and place-cell-like representations across mammals^46–48^ suggests that goal-directed sequential processing represents a fundamental computational principle. The goal-directed theta sweeps demonstrated here may represent predictive representations of task-relevant locations at later theta phases^49–51^ while retaining the representation of current location at early phases. The precise role of theta sweeps in supporting spatial planning could be causally assessed via interventions targeting specific theta phases or theta-related sequential dynamics in the entorhinal−hippocampal circuitry^25^. Our results suggest a plausible mechanism for incorporating goal direction into the hippocampal cognitive map^1^.

## Methods

### Behavioral and electrophysiological data

#### Honeycomb maze

Here we briefly summarize the experimental and data collection procedures used in our main analysis, and we refer readers to Ormond and O’Keefe^19^ for details. Five male Lister hooded rats, aged 9−12 months at time of electrophysiological recordings, were trained to navigate to a session-specific fixed reward location from different initial locations in the Honeycomb maze, up to the point when rats were consuming food on the reward platform without hesitation (Extended Data Fig. 1a–e). The Honeycomb maze consists of 61 tesselated hexagonal platforms in an overall hexagonal configuration (200 cm in width). Each platform can be independently raised and lowered, and the raised position is approximately 30 cm above the lowered position. After the initial platform was selected and raised, the animal was manually placed on the platform. Two randomly selected adjacent platforms are raised such that at least one of the platforms provides a position closer to the goal location than the current platform. After the animal has made its choice (indicated by continuous presence on the platform for 5 seconds), the remaining two platforms are lowered. In some choices, the two choice platforms are of equal distance to the goal, which we preclude from the analysis associated with correct and incorrect choices (Fig. 3 and Extended Data Fig. 7g–n). For the single-goal sessions used in the main analysis, each animal was recorded over two sessions on the Honeycomb maze, with interleaving open-field foraging periods, with 13 trials in the first session and seven trials for rats 2 and 3 and 13 trials for rats 1, 4, 5 in the second session. We pooled data from two sessions within the same day to form a single session for each rat. Each trial lasted between 15.63 seconds and 614.84 seconds (164.10 ± 107.02 seconds, averaged over all 118 trials). For the goal-switching session, all rats were first recorded over 13 trials to goal 1 (same goal as in the single-goal sessions), followed by interleaved ‘easy’ trials guiding animals to the alternative goal location and ‘no-reward’ trials that guide the animal back to the original goal location but without reward. Rats 1–4 were successfully trained to navigate toward the new goal locations (rat 2 required training over 6 days) and were subsequently recorded for 13 trials after switching within the same session. Rat 5 persisted in going toward the original goal after extended training and is, thus, excluded from all associated analyses (Extended Data Fig. 7a–f).

Neural recordings were obtained via an electrode array with 32 gold-plated tetrodes in the dorsal CA1 region of hippocampus, with recording frequency of 20 kHz for rat 1 and 30 kHz for rats 2–5. The numbers of neurons recorded per rat (putative place cells/all recorded neurons) were as follows: rat 1: 98/110; rat 2: 112/132; rat 3: 117/126; rat 4: 114/128; rat 5: 95/107. Behavioral recordings were based on two infrared LEDs positioned on top of the animals’ implant (one in the front and one behind, along the rostral−caudal axis), sampled at 25 Hz. Tracking of the LEDs was performed with DeepLabCut^54^. Vectors connecting the two LEDs determine the animal’s location (midpoint) and head direction. Histology showing recording sites in the CA1 layer of dorsal hippocampus can be found in Extended Data Fig. 2b.

#### Cheeseboard maze

We briefly summarize the experimental and data collection procedures in the Cheeseboard maze (Fig. 4 and Extended Data Fig. 8), and we refer readers to Pfeiffer and Foster^20^ for detailed description. Four male adult Long-Evans rats, aged 10–20 weeks, were trained on a foraging task in a 2-m × 2-m open-field arena, with 36 identical, evenly spaced reward wells over a 6 × 6 grid. All rats received training (in addition to pretraining on a linear track in a separate room) to perform an alternating homing/foraging task, where a session-specific fixed home well is initially filled at the start of the session. Once the rat discovered and consumed the reward at the home well (homing trial), the foraging trial started immediately, and a randomly chosen well was then filled for the rat to discover and consume. All four rats were recorded over one 30-minute sessions per day, over 2 days.

Upon reaching a performance threshold on the task, neural recordings were obtained via a microdrive array with 40 gold-plated tetrodes implanted in the CA1 region of dorsal hippocampus, with recording frequency of 32,556 Hz. The numbers of neurons recorded per rat (session 1 | session 2) were as follows: rat 1: 204/213 | 253/263; rat 2: 174/181 | 153/164; rat 3: 79/80 | 79/80; rat 4: 94/97 | 178/186. The behavioral recording was based on two distinctly colored, head-mounted LEDs, and the vector connecting the two LEDs determines the animal’s location (midpoint) and head direction. The behavioral data were recorded at 60 Hz and downsampled to 30 Hz for all analyses.

In both tasks, we analyzed the sorted spikes used in the original study. Putative place cells and inhibitory interneurons were classified based on mean firing rates and spatial information of firing rate maps^55^ (Extended Data Fig. 2d). The LFP signal was retrieved from one representative electrode from one selected tetrode in the Honeycomb task (note that we used MUA for extracting theta oscillations in the Cheeseboard task; see below).

#### Processing behavioral data

All second-order behavioral information, including speed, angular speed and movement direction, were estimated from tracking data after temporal smoothing with a ±150-ms boxcar window. The allocentric goal direction was computed as the moment-by-moment angle prescribed by head location (midpoint of the vector prescribed by the LEDs) and the goal location. All relative directions of the form ‘*x* versus *y*’ were computed by setting the *y* direction as the anchoring direction (0°) and then computing the signed angle of the *x* direction. For instance, ‘SD versus GD’ indicates the relative sweep direction with respect to the goal direction (with goal direction being the 0°).

#### Processing spiking data

In the Cheeseboard maze, hippocampal place cell data are constrained to periods above a certain speed threshold (>5 cm s^−1^) to ensure robust theta rhythmicity and reduce noise in spatial tuning. By contrast, in the Honeycomb maze task, the animal cannot move freely around the maze despite active engagement with the task contingency. We, hence, performed data exclusion for selecting valid theta cycles for our analysis, when the following criteria are met simultaneously for a minimum duration of 50 ms: (1) theta power (6–12 Hz) lower than 1 s.d. below the mean; (2) MUA greater than 2 s.d. above the mean; and (3) ripple power (100–250 Hz) lower than 1 s.d. below the mean. The spectral power was computed as the magnitude of the Hilbert-transformed LFP signal.

### Neural data analysis

#### Spatial decoding

Spatial decoding procedures for both tasks were largely similar, with only differences lying in the binning of the spatial enclosure. The two-dimensional Honeycomb maze is partitioned into 40 × 32 bins (approximately 5-cm bin size; compare to 50 × 50 approximately 4-cm bins in the Cheeseboard maze). The spatial tuning maps were computed as the smoothed (Gaussian kernel, s.d. 8 cm) firing rates (number of spikes divided by the dwell time) within each spatial bin. To reduce falsely high estimates of firing rates, we postprocessed the rate maps with Skaggs adaptive smoothing^56^. Specifically, we placed a circle centered at each bin location and gradually increased the radius (in bins) such that the spikes/dwell time within the expanded circle exceeded $\frac{\alpha }{{n}_{r}\sqrt{{S}_{r}}}\,$, where *α* is a constant and *n*_*r*_ and *S*_*r*_ are the number of 50-ms-long occupancy samples and number of spikes lying within the circle with radius *r*, respectively. We set *α* = 2,000 for both datasets. Only cells with peak firing rate (given the spatial tuning curves) >1 Hz were selected in downstream spatial decoding. The width of place fields was computed as the radius of the largest circle around the peak firing location with all rates within the circle greater than 50% of the peak firing rate.

We estimated the rat’s spatial location based on a memoryless maximum-likelihood decoding algorithm^26^ (also known as the Bayesian decoder, assuming uniform prior), using the unit-specific spatial rate maps and the population spike trains given temporal binning. Specifically, given a time window Δ*t*, we assume that each cell exhibits Poisson variability, and the associated firing is exclusively modulated by spatial location. Applying Bayesʼ rule, decoded spatial location is taken as the MAP estimate given the posterior distribution over spatial location

S1 $$\hat{{\boldsymbol{x}}}={argmax}_{{\boldsymbol{x}}}P\left({\boldsymbol{x}}|{\boldsymbol{S}}\right)={argmax}_{{\boldsymbol{x}}}\left({\prod }_{i=1}^{M}\left({\Delta }t\cdot {f}_{i}{\left(\left.{\boldsymbol{x}}\right)\right)}^{{s}_{i}}\right)\right)\exp \left.\left(-{\Delta }t\cdot {\prod }_{i=1}^{M}{\;f}_{i}\left({\boldsymbol{x}}\right)\right)\right),$$

where *f*_*i*_ (***x***) and *s*_*i*_ are the spatial tuning curve and number of emitted spikes within the selected time window of the *i*-th unit at location *x*, respectively. Note that we have assumed independent firing rates across all neurons. Bayesian decoding yielded accurate inference of the ground truth spatial location (Extended Data Fig. 3). Decoding accuracy (given temporal binning of 250 ms) was cross-validated by computing the decoding error for all valid periods within each trial, using rate maps constructed from all remaining trials. We included only putative place cells for spatial decoding, as the inclusion of inhibitory neurons leads to insignificant improvement in decoding accuracy (Extended Data Fig. 3b,c,f), largely due to the minimal gain in spatial information (Extended Data Fig. 2d). In addition to the MAP estimate, the full posteriors also reflected nontrivial uncertainty associated with spatial decoding. We, hence, additionally used the alternative estimate of the posterior mean to demonstrate the robustness of our findings (Supplementary Fig. 2):

S2 $$\widetilde{{\boldsymbol{x}}}=\mathop{\sum }\limits_{{\boldsymbol{x}}}P\left({\boldsymbol{x}}|{\boldsymbol{S}}\right).$$

We did not find any significant difference between firing properties of place cells under correct or incorrect choices (Extended Data Fig. 2); we, thus, included all valid periods in recorded trials, regardless of the choice correctness, in our spatial decoding and sweep analysis.

Across trials and spatial locations, we did not find significance difference in decoding accuracy between Bayesian decoders given all cells and only pyramidal cells in the Honeycomb maze (Extended Data Fig. 3); we, thus, proceeded with the latter in associated analyses. However, we found that decoding accuracy was significantly more accurate when using all recorded neurons instead of only pyramidal cells in the Cheeseboard task (Extended Data Fig. 8a–c); hence, we used all neurons for spatial decoding in associated analyses.

#### Theta sweep analysis

Theta cycles were retrieved from theta-filtered oscillations (6–12 Hz) of the LFP. The start and end of theta cycles were defined as the troughs of the Hilbert-transformed LFP. Using the Bayesian decoder, decoded position within each theta sequence was estimated over a phase window of 60° (equivalent to 14–28 ms), advancing in sliding phase window of 15° (equivalent to 3.5–7 ms).

We additionally performed analyses for theta sweeps given theta cycles identified from the MUA (sampled at 30 kHz) given all recorded neurons (inclusive of interneurons) for both the Honeycomb and Cheeseboard tasks, which is a smoothed histogram (Gaussian kernel, s.d. of 5 ms) over time (sampled at 1 kHz) (Fig. 4 and Supplementary Fig. 3). Notably, the sign of the recorded LFP activity can be arbitrary and is dependent on the actual location of the tetrode^57^. We, thus, apply an optional constant 180° phase shift to all detected LFP theta cycles such that the corresponding start and end times are maximally consistent with that of the MUA. For the Cheeseboard dataset, only MUA was used for extracting theta, as we have access to only one LFP channel per animal; hence, the arbitrary phase reversal in LFP could be mitigated by using the robust approximation of MUA.

Sample theta sweeps for visual demonstrations (Fig. 1d) were selected randomly from the pool of strongly goal-oriented theta sweeps (relative angle between sweep and goal directions within [−30°, 30°]), separately curated for periods when animals were moving toward, away from and sideways to the goal direction.

We observed that theta sweeps exhibit a cyclic pattern (Extended Data Fig. 4g–i). Thus, we included only the longest outward-extending subsequence^9,27^, lasting at least 180° (half cycle), of each theta sweep in our analysis while fixing the rat-specific start phase (given the 30° phase binning). Sweep directions and lengths were computed as the allocentric angle and magnitude of vectors connecting the decoding locations at endpoints, respectively. To ensure robust theta modulation, theta cycles during which the rat is moving >5 cm s^−1^ were included in the theta sweep analysis in the Cheeseboard maze, whereas the speed threshold was set to 2 cm s^−1^ in the Honeycomb maze due to the behavioral nature of the animal in the task configuration.

In plotting the roulette plot, we aggregated theta sweeps over equally sized angular bins (30°) of relative movement direction to the goal. Aggregation over sequence under varying rat poses in the environment requires standardizing the decoded MAP estimates according to the moment-by-moment position and movement direction of the rat, through translation and rotations, which yields the rat’s location at the origin, and movement direction pointing upwards after standardization.

S3 $${\bar{{\boldsymbol{x}}}}^{\phi }={f}_{{\rm{aggregate}}}\left({\left\{{\bar{{\boldsymbol{x}}}}_{i}^{\phi }\right\}}_{i}\right),\,{\bar{{\boldsymbol{x}}}}_{i}^{\phi }=\left[\begin{array}{cc}\cos \left({{\rm{\theta }}}_{{\rm{i}}}^{{\rm{\phi }}}\right) & -\sin \left({{\rm{\theta }}}_{{\rm{i}}}^{{\rm{\phi }}}\right)\\ \sin \left({{\rm{\theta }}}_{{\rm{i}}}^{{\rm{\phi }}}\right) & \cos \left({{\rm{\theta }}}_{{\rm{i}}}^{{\rm{\phi }}}\right)\end{array}\right]\cdot \left({\hat{{\boldsymbol{x}}}}_{i}^{\phi }-\,{{\boldsymbol{x}}}_{i}^{\phi }\right),$$

where ${\bar{{\boldsymbol{x}}}}_{i}^{\phi }$ is the standardized MAP estimate (after translation and rotation), and ${x}_{i}^{\phi }$ and ${\theta }_{i}^{\phi }$ are the rat’s location and relative movement direction to the goal, at phase bin *ϕ* of the *i*-th sequence. To mitigate potential bias introduced by extreme events, the aggregation function *f*_aggregate_ (⋅) is taken to be the median of decoded values. Due to the varying lengths of sequences, the sample size of the averaging varies within different phase bins. We found that this leads to little effect in practice, due to the small variations in the lengths of selected theta sweeps (202.42° ± 17.02°).

Quantitative comparisons of alignment between sweep directions and offset directions, relative to goal, movement and head directions, were based on the Rayleigh concentration (or, equivalently, the MRL) of the circular distributions around its mean (which is usually close to 0°).

S4 $$R\left(\theta \right)=\frac{1}{N}\sqrt{{\left.\left({\sum }_{i=1}^{N}\cos \left({\theta }_{i}\right)\right)\right)}^{2}+{\left.\left({\sum }_{i=1}^{N}\sin \left({\theta }_{i}\right)\right)\right)}^{2}\,},$$

where $\theta ={\left\{{\theta }_{i}\right\}}_{i=1}^{N}$ is the set of observations of the angular variable of interest.

#### Replay sequence analysis

We restricted our detection of replay events within sharp-wave ripples, given the MUA of all clustered units. Events during which the animal’s speed is <2 cm s^−1^ and the MUA is greater than 3 s.d. from the mean, and which last between 65 ms and 1,000 ms, were considered a valid candidate event for replay sequence decoding. Candidate events separated by less than 50 ms were merged and considered to be a single event. We focused our analysis on ‘distant’ replay events, such that candidate events during which the animal’s location was less than 50 cm from the goal location were excluded from analysis. Bayesian decoding of spatial locations (equation (S1)) within replay events were based on temporal binning of 20 ms with a 5-ms sliding window. Following Pfeiffer and Foster^20^, each replay sequence was truncated to the longest subsequence of time bins (but constrained to be >50 ms) with peak posterior probability less than 20 cm between consecutive time bins. We used similar standardization procedures described above for aggregating theta sequences (equation (S3)) but with additional scale normalization, such that the goal location was always placed five bins away from the animal’s location in the upward direction after standardization (similarly for the aggregation over the full decoding posteriors; Fig. 6d,f). For minimizing the effect of noisy decoding, we estimated the direction of replay sequences as the angle prescribed by the vector connecting the expected locations with respect to the averaged decoding posteriors aggregated over the first and second half of each candidate replay event. Replay sequence lengths are given by the lengths of the same vectors.

#### Phase coding analysis

To investigate phase coding with respect to distance traveled toward or away from the goal (Fig. 7a–c), we constrained our analysis to periods of continuous movement toward or away from the goal, lasting more than 800 ms (approximately eight theta cycles) for robust examination of phase coding. Additionally, we allow sporadically brief discontinuity in the movement direction, by including trajectories with brief gaps (<150 ms of reversed direction), in order to maximize data utilization. Normalized distance to the goal is computed as the path length along the trajectory (reversed when moving away from the goal), normalized between 0 and 1.

#### Statistical analysis

##### Rat-level statistical tests

To avoid pseudo-replication, our preliminary analyses treated the rat (or the recording session in the Cheeseboard maze) as the unit of inference. Specifically, for each rat/session, we first aggregated across all sweeps or replay events to obtain a single summary statistic (for example, MRL of sweep direction distributions or mean sweep lengths). We then compared these rat/session-level summary values between conditions using two-sided tests (or one-sided test when there is strong prior hypothesis about the relationship between the two groups under comparison). We used Studentʼs *t*-tests as the default statistical test. Some comparisons violate assumptions under *t*-tests—for example, the normality assumption for speed/angular speed comparison due to right-skewness and unequal variances for comparison between theta sequences and replay sequences. In these cases, we used nonparametric tests such as Wilcoxon signed-rank test for paired comparisons and Mann−Whitney *U*-test for unpaired comparisons. Following the general wisdom, *r* < 0.2 is considered a minor effect size (Fig. 8e). For all rat-level tests, we report *P* values, test statistics and degrees of freedom (or sample size for nonparametric tests). We note that, for *P* values, we state *P* < 10^−308^ whenever the true *P* value is less than the smallest numerically calculable values.

##### Individual event-level tests

In addition to the rat-level comparison, we also performed LMM analysis^58^ to make full use of the data at individual event level while accounting for hierarchical dependence. For instance, multiple theta sweeps within a session are derived from spatial decoding of the same simultaneously recorded neuronal population, and sessions are nested within animals; consequently, treating sweeps as independent observations would inflate degrees of freedom. LMMs allow fixed effects of experimentally defined predictors (for example, trial type, alignment between movement and goal direction or reference direction category) to be estimated while modeling correlation structure induced by repeated measurements within the same animal and/or session via random effects. For each dependent variable *y*_*i*_ (for example, cosine similarity between sweep or replay direction and goal direction; sweep length; and movement speed within each theta cycle), we fit linear models of the following form:

$${{\rm{y}}}_{{\rm{i}}}={{\beta}}_{0}+\mathop{\sum}\limits_{k=1}^{p}{\beta }_{k}{u}_{{ik}}\,+\mathop{\sum}\limits_{m=1}^{q}{b}_{g\left(i\right),m}{z}_{{im}}+{\epsilon }_{i},$$

where ${u}_{{ik}}$ is the *k*-th fixed-effect predictor (for example, reference direction or trial type) for the *i*-th observation (for example, animal or session nested within animal), with coefficient ${\beta }_{k}$ and corresponding grouping $g\left(i\right)$, and ${z}_{{im}}$ are the corresponding random-effect variables with group-specific coefficients ${b}_{g\left(i\right),{m}}$. Random effects are assumed to be a priori Gaussian distributed, and noise terms are assumed independent conditioned on the random effects (${\epsilon }_{i}{\mathscr{\sim }}{\mathscr{N}}(0,\,\sigma ^2$). In some cases, we include additional second-order interaction terms between existing fixed-effect predictors with corresponding coefficients (for example, trial type × reference direction interaction in the Cheeseboard maze). Fixed-effect significance is assessed using two-sided Wald tests unless otherwise stated. Note that, in testing for alignment strength between sequence directions (sweep or replay) and relative directions (head/movement/goal; for example, Extended Data Figs. 4d,e,l, 5d,g, 6b, 7i, 8m and 10 and Supplementary Fig. 2h), we chose to use cosine similarity of the relative angles as the dependent variable, transforming circular distances into a bounded linear scale [−1, 1] where the assumption of Gaussian noise is more likely to hold. Notably, assuming that mean angular difference is sufficiently close to zero, MRL is the population-level summary of cosine similarities at the individual level, hence connecting the LMM analysis and the population analysis (at the rat level).

##### Permutation/shuffled tests

We used data shuffling to construct the null hypothesis distributions wherever necessary. For example, in assessing the effect of goal modulation in sweep direction in the Honeycomb maze, movement modulation is a potential confound (despite absence of frequent goalward movement). Regressing out the effect of movement direction with circular regression^53^, we computed the MRL of the relative angles between residual sweep directions and goal directions. We then shuffled the relationship between movement and goal directions and performed the same residual analysis for each random shuffle and compared the ground truth MRL with the shuffled values (Fig. 1g–j).

#### Detecting neurons with significant egocentric goal-directional tuning

To identify neurons with significant egocentric goal-directional tuning (Extended Data Fig. 5), we fitted the following position-augmented linear−nonlinear Poisson spiking models to single-cell spiking for all recorded pyramidal neurons^59^:

$${n}_{{it}}\sim \mathrm{Poisson}\left({r}_{i,\,{\boldsymbol{x}}\left({\boldsymbol{t}}\right)}+\mathop{\sum }\limits_{k=1}^{p}{w}_{{ik}}{u}_{{kt}}\right),$$

where *n*_*it*_ denotes the number of spikes emitted by the *i*-th neuron in the *t*-th time bin (with 20-ms temporal binning); ${r}_{i,\,{\boldsymbol{x}}\left(t\right)}$ is the positional firing rate of the *i*-th neuron at the location at time *t*; and ***x***(*t*); *u*_*kt*_ denotes the *k*-th additional predictor value at time *t* (for example, head direction or egocentric goal direction) with corresponding coefficient for the *i*-th neuron, ${w}_{{ik}}$. We considered two instantiations of such models: the base model with only head direction as the additional predictor and an alternative model with both head direction and egocentric goal direction as predictors. For all pyramidal neurons, we fitted the parameters for both models and computed the difference between their evidence. We then performed one-sided shuffled testing (over 1,000 random seeds) over egocentric goal directions (given random time shifts without breaking the temporal continuity): fitting the alternative model given the shuffled egocentric goal directions and computing the likelihood difference with respect to the base model, and a cell is identified as exhibiting significant tuning to egocentric goal direction if the *P* value (probability of random shuffles with likelihood difference greater than that the difference with respect to the unshuffled alternative model) is less than 0.05.

### Mechanistic modeling with CANs

Conceptually, goalward directional sweeps drive activation of neurons in their downstream network of conjunctive grid cells with preferred direction of firing equal to the internal direction of directional cell sweeps. The activity bump over the two-dimensional neuronal sheet in the conjunctive grid cell network provides external phase shift input to the grid cell network, driving grid cell activity sweeping along the goal direction. The activity bump of the place cell network is then a feedforward projection of the grid call activity bump, which effectively inherits the geometric property of sweeps in grid cell activity. In actual simulations, the projected signals along the directional cells−conjunctive grid cells−grid cells−place cells pathway was abstracted into an effective directionally gated spatial input to the place cell network, directly projected from directional cells (equation (S16)).

Below, we provide mathematical details of each subcomponent of the model. Parameters used in all simulations (Fig. 5 and Extended Data Fig. 9) can be found in Supplementary Table 1. All simulations were numerically integrated with the exponential Euler method^60^ and were implemented using the BrainPy library^61^. All simulation results were based on 10 random seeds unless stated otherwise.

#### Directional ring attractor

Neurons in the directional cells ring attractor (for example, parasubicular direction cells) are organized over a ring, equally spaced between 0° and 360°, with each cell tuned to a specific ‘internal direction’in the allocentric reference frame^13^. Directional cells receive top-down goal-oriented directional inputs, are modulated by theta rhythmicity and exhibit firing rate adaption^9,62^.

S5 $${\tau }_{h}\frac{\partial {h}_{i}}{\partial t}=-{h}_{i}+\mathop{\sum}\limits_{j=1}^{{N}_{h}}{\;J}_{{ij}}^{\;h}{\;f}_{h}\left({h}_{j}\right)-{a}_{i}^{h}+{I}_{i}^{h}\left({v}_{t},\,{\theta }_{t}\right)+{I}_{i}^{{\rm{td}}}\left({\theta }_{t},\,{\varphi }_{t}\right),\,{\rm{for}}\,i=1,\,\ldots ,\,{N}_{h},$$

where *τ*_*h*_ is the time constant; *h*_*i*_ is the presynaptic input to the *i*-th directional cell; *N*_*h*_ is the number of neurons in the directional cell network; ${a}_{i}^{h}$ denotes the corresponding firing rate adaptation modulation; ${I}_{i}^{h}\left({v}_{t},\,{\theta }_{t}\right)$ denotes the directional-dependent external sensory inputs that are jointly modulated by speed and theta rhythmicity; ${I}_{i}^{\mathrm{td}}\left({\theta }_{t},\,{\varphi }_{t}\right)$ denotes the top-down goal-modulated directional input; and *v*_*t*_ *θ*_*t*_ and *φ*_*t*_ are the animal’s speed, allocentric head direction and goal direction, respectively.

The external sensory input is modeled with a squared-exponential response function, with peak at the animal’s current head direction, *θ*_*t*_.

S6 $${I}_{i}^{h}\left({v}_{t},{\theta }_{t}\right)={A}_{h}{\alpha }_{h}\left({v}_{t}\right)\exp \left.\left(-\frac{{\left|{\psi }_{i}-\,{\theta }_{t}\right|}^{2}}{4{\sigma }_{h}^{2}}\right)\right),$$

where ${\psi }_{i}$ represents the preferred internal direction of the *i*-th neuron (in radians); $\left|{\psi }_{i}-{\theta }_{t}\right|=\min (\mathrm{mod}\left({\psi }_{i}-{\theta }_{t},\,2\pi \right),\,2\pi -\mathrm{mod}\left({\psi }_{i}-{\theta }_{t},\,2\pi \right)$ represents circular distance between the internal direction for the *i*-th neuron and current head direction; ${\sigma }_{h}$ controls the tuning width of sensory input; ${A}_{h}$ denotes the strength of sensory input; and ${\alpha }_{h}\left({v}_{t}\right)$ denotes the speed-regulated theta modulation from medial septum^1^, in the form of sinusoidal waves.

S7 $${\alpha }_{h}\left({v}_{t}\right)=1+{\bar{\alpha }}_{h}{v}_{t}\cos \left({\omega }_{t}\right),$$

where ${\bar{\alpha }}_{h}$ denotes the strength of theta modulation, and *ω*_*t*_ is the theta phase at time *t*. Note that we assume the preferred internal directions of neurons in the directional cell network tile the [0, 2*π*] space with equal spacing.

The top-down goal-oriented directional input, potentially originating in the retrosplenial cortex^40,41,63^, is also modeled with a squared-exponential tuning, with peak at the allocentric goal direction, *φ*_*t*_.

S8 $${I}_{i}^{{\rm{td}}}\left({\theta }_{t},\,{\varphi }_{t}\right)={A}_{h}^{{\rm{td}}}\exp \left.\left(-\frac{{\left|{\psi }_{i}-{\varphi }_{t}\right|}^{2}}{4{\gamma }_{h}^{2}}\right)\right)$$

where ${A}_{h}^{{\rm{td}}}$ and ${\gamma }_{h}^{2}$ denote the strength and tuning width of top-down directional inputs, respectively.

The nonlinear activation function is modeled as second-order global inhibition.

S9 $${f}_{h}\left({h}_{j}\right)=\frac{{\left[{h}_{j}\right]}_{+}^{2}}{1+{k}_{h}{\sum }_{l=1}^{{N}_{h}}{\left[{h}_{l}\right]}_{+}^{2}},$$

where *k*_*h*_ represents the global inhibition strength within the directional cell network, and [⋅]_+_=max(⋅, 0) denotes the rectified linear unit (ReLU) transformation. Previous work has shown that global inhibition is crucial for maintaining a localized activity bump on the ring attractor^37,64^. Note that there were no nonlinear transfer functions for ensuring positive firing activity in the classical CAN literature^65^. However, to avoid ambiguity about negative inputs, we first transformed the presynaptic inputs through a ReLU nonlinearity before applying the divisive normalization. Incorporating the additional ReLU nonlinearity yielded indistinguishable results for the main simulations (goal-directed bias, sweep length effects and phase coding). To avoid further ambiguity about non-negative firing neurons, we adopted the ReLU nonlinearity in all our current simulations (equations (S9) and (S13)).

The recurrent connections between neurons *i* and *j*, with preferred firing directions *ψ*_*i*_ and *ψ*_*j*_ (in radians), respectively, are defined by a circular squared exponential connectivity.

S10 $${J}_{{ij}}^{h}=\frac{{J}_{0}^{h}}{2\pi {\eta }_{h}^{2}}\exp \left.\left(-\frac{{\left|{\psi }_{i}-\,{\psi }_{j}\right|}^{2}}{2{\eta }_{h}^{2}}\right)\right),$$

where ${J}_{0}^{h}$ and *η*_*h*_ control the strength and tuning width of the directional cell connectivity, respectively.

The firing rate adaptation ${a}_{i}^{h}$ is implemented as a negative feedback that induces dynamic reduction in the neuron’s firing rate in response to constant-intensity input^21,66^.

S11 $${\tau }_{h}^{a}\frac{\partial {a}_{i}^{h}}{\partial t}=-{a}_{i}^{h}+{m}_{h}{f}_{h}\left({h}_{i}\right),$$

where ${\tau }_{h}^{a}$ is the time constant of firing rate adaptation, and ${m}_{h}$ represents the adaptation strength. The time constant ${\tau }_{h}^{a}$ is usually set to be a magnitude greater than ${\tau }_{h}$, modeling the fact that firing rate adaptation is a slower dynamic than spiking. At the network level, firing rate adaptation induces nontrivial intrinsic dynamics to the activity bump, leading to sequential activation of neurons in the absence of sensory updates.

#### Place cell attractor network

Place cells are assumed to lie on a two-dimensional neural sheet, with equally spaced field centers. The place cell network receives spatial sensory inputs and theta-modulated directionally shifted inputs from conjunctive grid cells, driven by the projection of directional cell activities.

S12 $${\tau }_{p}\frac{\partial {p}_{i}}{\partial t}=-{p}_{i}+\mathop{\sum }\limits_{j=1}^{{N}_{p}}{\;J}_{{ij}}^{\,p}{\;f}_{p}\left({p}_{j}\right)-{a}_{i}^{p}+{I}_{i}^{p}\left({{\boldsymbol{x}}}_{t}\right)+{I}_{i}^{{\rm{gc}}}\left({{\boldsymbol{v}}}_{t},\,{\theta }_{t},\,{{\boldsymbol{x}}}_{t}\right),{\rm{for}}\;\;i=1,\,\ldots ,\,{N}_{p},$$

where *τ*_*p*_ is the time constant; *p*_*i*_ is the presynaptic input to the *i*-th place cell; *N*_*p*_ is the number of place cells; ${a}_{i}^{p}$ denotes the firing rate adaptation; ${I}_{i}^{p}\left({{\boldsymbol{x}}}_{t}\right)$ denotes the spatially selective external sensory input at location ***x***_*t*_; and ${I}_{i}^{{\rm{gc}}}$ (***v***_*t*_, *θ*_*t*_, ***x***_*t*_) denotes the directionally shifted input from conjunctive grid cells with joint modulations from theta oscillations and speed.

We assume that the global inhibitory normalization of place cell activity is of the same form as for head direction cells (equation (S9)) but with different inhibition strength, *k*_*p*_, and an additional population-wide gain factor, *g*.

S13 $${f}_{p}\left({p}_{j}\right)=\frac{g\cdot {\left[{p}_{j}\right]}_{+}^{2}}{1+{k}_{p}{\sum }_{l=1}^{{N}_{p}}{\left[{p}_{l}\right]}_{+}^{2}},$$

Similarly, we assume Gaussian-like recurrent connections between place cells *i* and *j*, with field centers ${{\boldsymbol{s}}}_{i}$ and ${{\boldsymbol{s}}}_{j}$.

S14 $${J}_{{ij}}^{p}=\frac{{J}_{0}^{p}}{2\pi {\eta }_{p}^{2}}\exp \left.\left(-\frac{{{{\boldsymbol{||}}{\boldsymbol{s}}}_{i},\,{\,{\boldsymbol{s}}}_{j}{\rm{||}}}^{2}}{2{\eta }_{p}^{2}}\right)\right),$$

where ${{\boldsymbol{||s}}}_{i},\,{\,{\boldsymbol{s}}}_{j}{||}$ denotes the Euclidean distance between field centers ${{\boldsymbol{s}}}_{i}$ and ${{\boldsymbol{s}}}_{j}$, and ${J}_{0}^{p}$ and *η*_*p*_ control the strength and tuning width of the place cell connectivity, respectively.

The strength of external sensory input is also modeled with Gaussian tuning, with peak at the allocentric spatial location ***x***_*t*_.

S15 $${I}_{i}^{p}\left({{\boldsymbol{x}}}_{t}\right)=\exp \left.\left(-\frac{{{{||}}{{\boldsymbol{x}}}_{t},{{\boldsymbol{s}}}_{i}{\rm{||}}}^{2}}{4{\sigma }_{p}^{2}}\right)\right),$$

As discussed above, the computations afforded by entorhinal cells can be simplified and viewed as implementing a mapping from an internal directional state (set by the directional cells ring attractor) to a directionally structured spatial drive that steers downstream spatial encoding activity. When the downstream place cell network receives inputs that are already spatially tuned and directionally shifted, the specific intermediate implementation of a grid cells attractor is not identifiable from the place cell sweep statistics. Accordingly, we replaced the explicit grid cell layers with the following effective input term to the place cell network, directly projected from directional cells:

S16 $$\begin{array}{l}{I}_{i}^{gc}\left({{\boldsymbol{v}}}_{t},{\theta }_{t},\,{{\boldsymbol{x}}}_{t}\right)={\alpha }_{p}\left({v}_{t}\right){\beta }_{p}\left({v}_{t}\right)\frac{{\sum }_{j=1}^{{N}_{h}}{{ {\mathbb{I}} }}\left.\left({f}_{h}\left({h}_{j}\right) > \nu \,{\max \left\{{f}_{h}\left({h}_{l}\right)\right\}}_{l=1}^{{N}_{h}}\right)\right){I}_{ij}^{\mathrm{conj}-\mathrm{gc}}\left({{\boldsymbol{x}}}_{t}\right)}{{\sum }_{j=1}^{{N}_{h}}{{ {\mathbb{I}} }}\left.\left({f}_{h}\left({h}_{j}\right) > \nu \,{\max \left\{{f}_{h}\left({h}_{l}\right)\right\}}_{l=1}^{{N}_{h}}\right)\right)},\\ \,\,\,\,\,\,{I}_{ij}^{\mathrm{conj}-\mathrm{gc}}\left({{\boldsymbol{x}}}_{t}\right)=\exp \left.\left(-\frac{{\left\langle {{\boldsymbol{x}}}_{t}+{{\boldsymbol{w}}}_{j}\left({\theta }_{t}\right),\,{{\boldsymbol{s}}}_{i}\right\rangle }^{2}}{4{\gamma }_{p}^{2}}\right)\right),\\ \,\,\,\,\,\,\,\,\,\,\,\,\,\,\,\,{{\boldsymbol{w}}}_{j}\left({\theta }_{t}\right)={w}_{0}{\left[cos\left({\psi }_{j}\right),sin\left({\psi }_{j}\right)\right]}^{T}+\,{w}_{1}{\left[cos\left({\theta }_{t}\right),\,sin\left({\theta }_{t}\right)\right]}^{T},\end{array}$$

where the strength of input scales linearly with running speed.

S17 $${\beta }_{p}\left({v}_{t}\right)={\beta }_{0}{v}_{t}+{C}_{p},$$

for constants *β*_0_ and *C*_*p*_. ${I}_{{ij}}^{{\rm{conj}}-{\rm{gc}}}\left({{\boldsymbol{x}}}_{t}\right)$ represents the Gaussian-shaped projection strength from the directionally-shifted activity bump of conjunctive grid cells with preferred internal direction *ψ*_*j*_, at the current location ***x***_*t*_. The internal direction can reflect both the goal and the current head/movement direction. ${\mathbb{I}}\left(\cdot \right)$ is the indicator function, modeling a soft winner-takes-all thresholding mechanism, given *ν*∈ [0, 1] that sets the threshold on firing rates of head direction cells. The subset of idealized conjunctive grid cells with preferred directions aligned with directional cells whose firing rate supersedes the threshold are activated, effectively ensuring that only conjunctive grid cells with preferred internal direction lying along the current goal direction contributing to the formation of directionally shifted spatial input. Notably, this simplification does not assume that grid cell layers are absent or unimportant; rather, it treats them as a latent transformation whose output to hippocampus can be parameterized directly and whose detailed internal dynamics are outside the scope of the present study.

We additionally induce speed-regulated septal theta modulation, *α*_*p*_ (*v*_*t*_), given that conjunctive grid cells are tuned to theta rhythm^52^.

S18 $${\alpha }_{p}\left({v}_{t}\right)=\left(1+{\bar{\alpha }}_{p}{v}_{t}\cos \left({\omega }_{t}\right)\right),$$

where ${\bar{\alpha }}_{p}$ is the strength of theta modulation, and ${\omega }_{t}$ denotes the phase of the simulated theta rhythmicity at time *t*.

Place cells follow the same firing rate adaptation mechanism as the grid cells, with time constant ${\tau }_{p}^{a}$ and adaptation strength *m*_*p*_.

#### Simulated trajectory

Simulated trajectories were designed to capture the dominant features in the rat’s behavior in the Honeycomb maze (Extended Data Fig. 4a). For simplicity, we model the rat body as a rigid stick, with the outward end representing the head location, which enables efficient simulation of rotational scanning movements. We interleave random walk and random head rotation movements, with constant speeds. At each timepoint (simulated at 1,000 Hz), with 0.05 probability, the simulated rat undergoes a rotational scanning movement spanning angles sampled uniformly from [0.3*π*, *π*] (Fig. 5c).

#### Theta sweep decoding and phase coding with mechanistic simulations

The proposed CAN is rate based by design, and, instead of instantiating a spiking model for decoding theta sweep based on generated spikes, we use the peak location of the place cell activity bump as a proxy for the decoded location, which is unbiased on expectation.

To simulate phase precession/procession, we generated Poisson spikes given the instantaneous firing rate of each place cell^67^. To reduce visual clutter, we apply a gain factor of 0.05 to place cell firing rates to reduce the total number of spikes emitted.

### Reporting summary

Further information on research design is available in the Nature Portfolio Reporting Summary linked to this article.

## Online content

Any methods, additional references, Nature Portfolio reporting summaries, source data, extended data, supplementary information, acknowledgements, peer review information; details of author contributions and competing interests; and statements of data and code availability are available at https://doi.org/10.1038/s41593-026-02365-2.

## Supplementary information


Supplementary InformationTable 1 and Figs. 1–3.
Reporting Summary
Peer Review file


## Source data


Source Data Fig. 1Statistical source data
Source Data Fig. 2Statistical source data
Source Data Fig. 3Statistical source data
Source Data Fig. 4Statistical source data
Source Data Fig. 5Statistical source data
Source Data Fig. 6Statistical source data
Source Data Fig. 7Statistical source data
Source Data Fig. 8Statistical source data
Source Data Extended Data Fig./Table 1Statistical source data
Source Data Extended Data Fig./Table 2Statistical source data
Source Data Extended Data Fig./Table 3Statistical source data
Source Data Extended Data Fig./Table 4Statistical source data
Source Data Extended Data Fig./Table 5Statistical source data
Source Data Extended Data Fig./Table 6Statistical source data
Source Data Extended Data Fig./Table 7Statistical source data
Source Data Extended Data Fig./Table 8Statistical source data
Source Data Extended Data Fig./Table 9Statistical source data


## Data Availability

The analyses in this study used previously published datasets from Ormond and O’Keefe^19^ (Honeycomb maze) and Pfeiffer and Foster^20^ (Chessboard task). Source data underlying the figures in this paper are provided with the paper. The Honeycomb maze dataset is available from the corresponding authors of the present study upon reasonable request. The Pfeiffer and Foster dataset was generated by the original authors and is not redistributed by the present authors; requests for access should be directed to the corresponding author(s) of Pfeiffer and Foster^20^. Source data are provided with this paper.

## Code availability

The custom Python code used for all analyses and model simulations can be accessed through the GitHub repository: https://github.com/changmin-yu/goal_directed_theta_sweeps_honeycomb_maze.
