# Supplementary material for: Hippocampal theta sweeps indicate goal direction during navigation
Source: Nat Neurosci. 2026 Jul 1;29(9):2225–36. doi: 10.1038/s41593-026-02365-2 (PMC13533849; doi:10.1038/s41593-026-02365-2)
Supplement: Supplementary file 1 [file 41593_2026_2365_MOESM1_ESM.pdf]

nature neuroscience

Supplementary information

https://doi.org/10.1038/s41593-026-02365-2

Hippocampal theta sweeps indicate goal direction during navigation

In the format provided by the authors and unedited

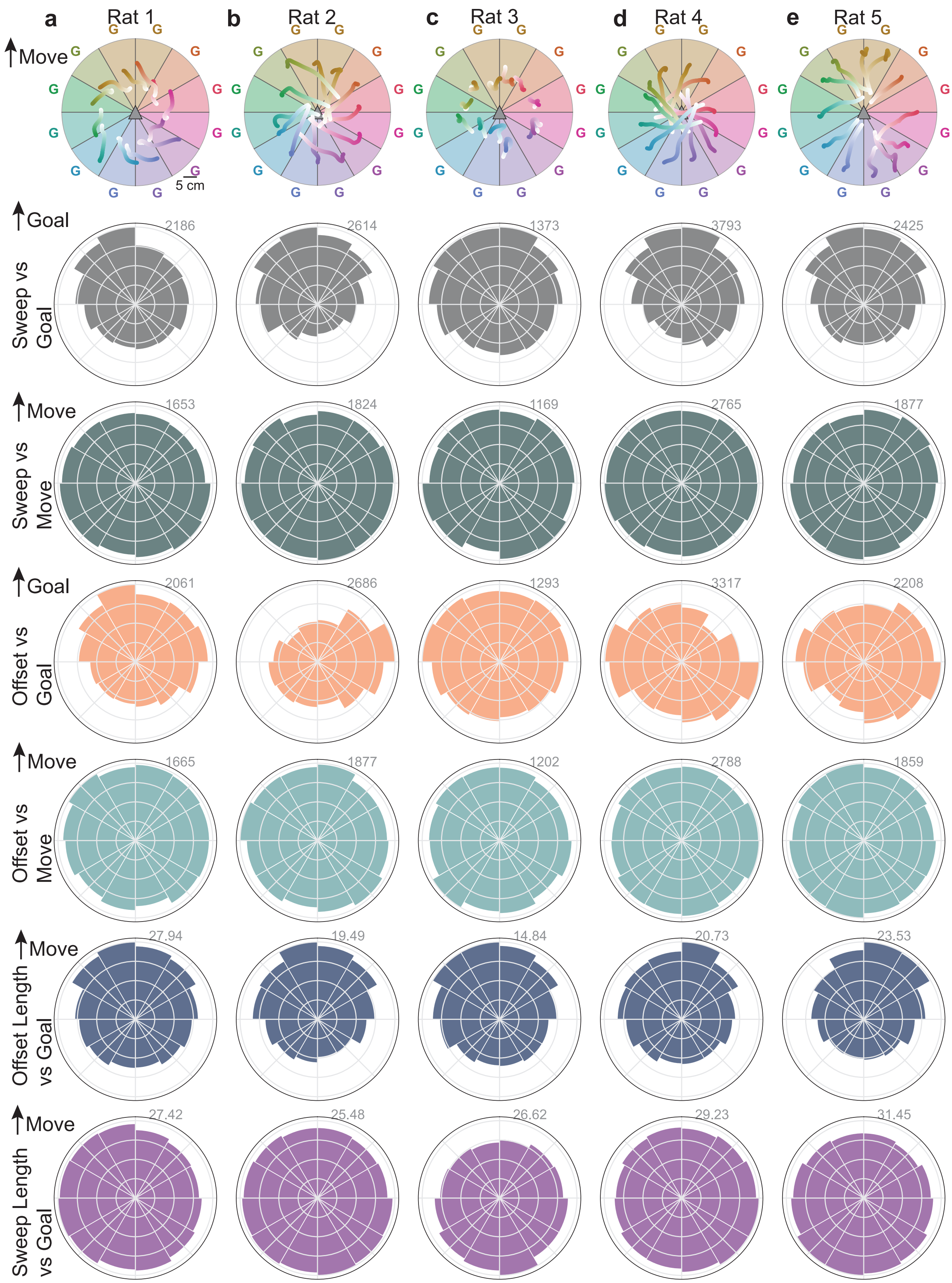
a
b
c
d
e
Rat 1
Rat 2
Rat 3
Rat 4
Rat 5
Move
5 cm
Goal
Sweep vs Goal
Move
Sweep vs Move
Goal
Offset vs Goal
Move
Offset vs Move
Move
Offset Length vs Goal
Move
Sweep Length vs Goal
2186
2614
1373
3793
2425
1653
1824
1169
2765
1877
2061
2686
1293
3317
2208
1665
1877
1202
2788
1859
27.94
19.49
14.84
20.73
23.53
27.42
25.48
26.62
29.23
31.45

Supplementary Figure S1. **Goal-oriented theta sweeps in individual rats. a.** Rat 1. From top to bottom: median theta sweeps as a function of relative movement direction (upward) to the goal; circular histogram of relative angles between sweep- and goal-directions; circular histogram of relative angles between sweep- and movement-directions; circular histogram of relative angles between initial offset- and goal-directions; circular histogram of relative angles between initial offset-direction and movement-direction; median lengths of initial offset of decoded theta sweeps given varying relative movement direction to the goal; median lengths of theta sweeps given varying relative movement direction to the goal. **b-e.** Same as (a), but for Rats 2 - 5. Data from individual rats yield similar qualitative observations as in the aggregated analysis (Figure 1): theta sweeps within individual rats exhibit strong goal-oriented direction bias.

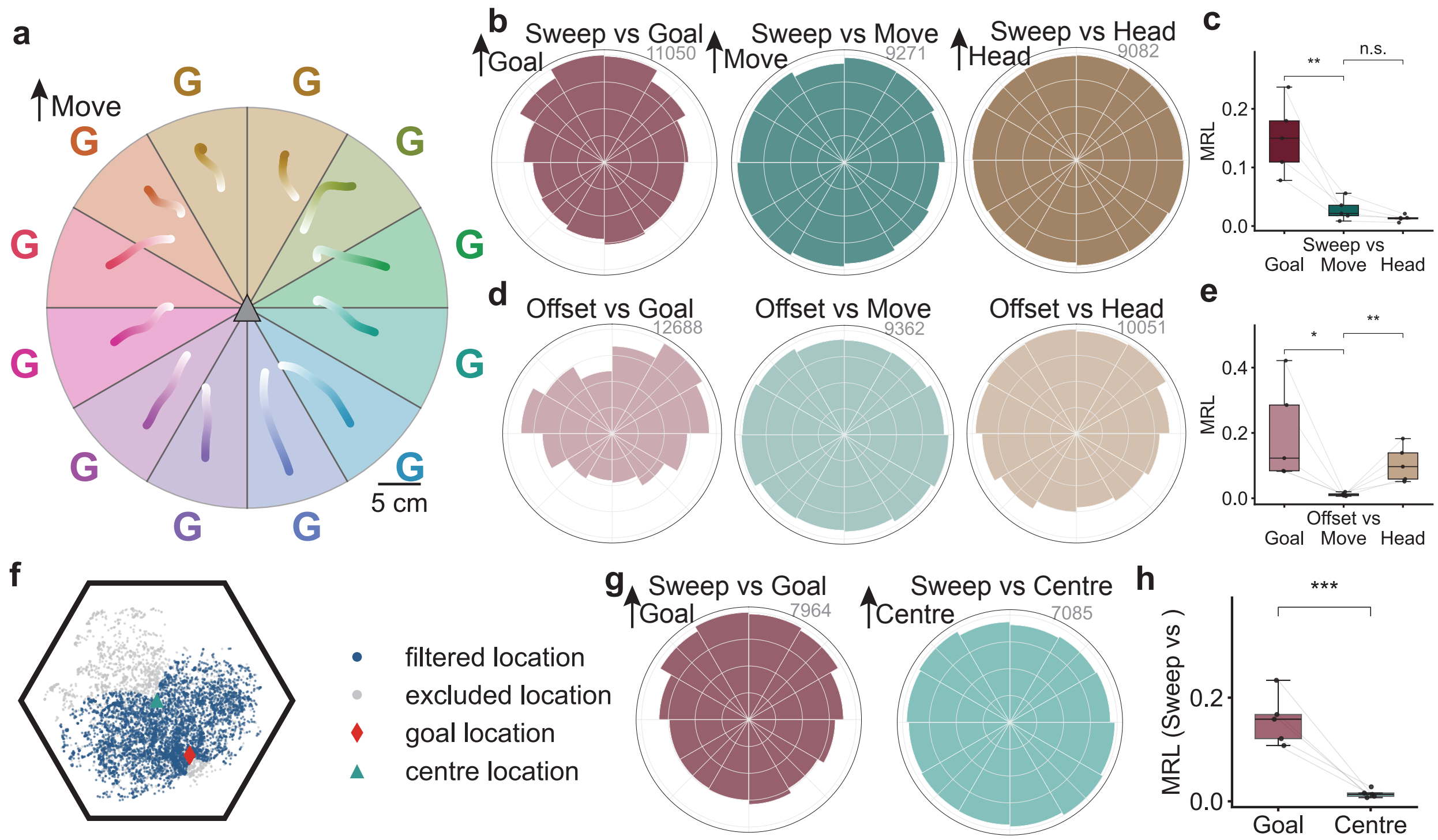

Supplementary Figure S2. **Goal-oriented directional bias in theta sweeps persists given theta sweeps computed using posterior expectations.** We note that theta sweeps were defined by the decoded locations in the main analysis, i.e. choosing the *maximum-a-posteriori* estimate from the probability distribution at each timestep, and subsequently aggregated across trials. Here we take expectations with respect to the full posterior distributions rather than taking the single most likely location as decoded sweep locations. **a.** Median theta sweeps as a function of varying relative movement direction (upwards) to the goal. **b.** Distributions of relative angles between sweep direction and goal direction (left), sweep direction and movement direction (middle), and sweep direction and head direction (right), aggregated over all valid theta sweeps (one-sided shuffled test against chance-level with 10,000 shuffles; Sweep vs Goal: $p<0.0001$; Sweep vs Move: $p=0.0003$; Sweep vs Head: $p=0.084$). **c.** MRL of rat-specific distributions in **b** (two-sided paired t-test, n=5 animals; Goal vs Move: $p=0.0028$, $t=4.2593$; Move vs Head: $p=0.1341$, $t=1.6668$; d.f.=4). **d.** Same as **b**, but for the distributions with respect to initial offset directions (one-sided shuffled test; Offset vs Goal: $p<0.0001$; Offset vs Move: $p=0.0019$; Offset vs Head: $p<0.0001$). **e.** Same as **c**, but for distributions in **d** (two-sided paired t-test, n=5 animals; Goal vs Move:

p=0.0231, t=2.8014; Move vs Head: p=0.0055, t= -3.7687; d.f.=4). **f.** Demonstration of control for centroid bias. Taking expectations with respect to the posterior distribution can bias decoded locations towards the centre of the environment. Here we control for such bias and remove theta sweeps with the corresponding rat's location (approximately) lying along the extension of the line connecting the goal and centre locations (± 30°). Additional analysis on filtered sequences removes potential confound arising from overlapping directions to the goal and centre. **g.** Distribution of relative sweep direction relative to the goal (left) and environment centre (right) direction, over all filtered theta sweeps (one-sided shuffled test; Sweep vs Goal: p<0.0001; Sweep vs Centre: p=0.2307). **h.** MRL of relative sweep direction with respect to the goal direction and centroid direction, over filtered theta sweeps (one-sided paired t-test: p=0.0002, t=6.4021; d.f.=4). We performed additional analysis on the individual sweep-level via fitting LMMs for predicting the cosine similarity between sweep and reference directions given categorical predictors (reference direction: movement/head/goal/centroid directions), taking into account the nested data structure. Fitted LLM yielded significant decrease in predicted alignment between sweep directions and non-goal reference directions (reference movement direction: β= -0.1311, z= -43.0605, p=0.0; reference head direction: β= -0.1673, z= -54.7004, p=0.0; reference centre direction: β= -0.2008, z= -65.4404, p=0.0). Similar to the main results, decoded theta sweeps given the full decoding posterior preferentially extended towards the goal direction (and not the centroid direction despite the potential centroid bias of the decoding method).

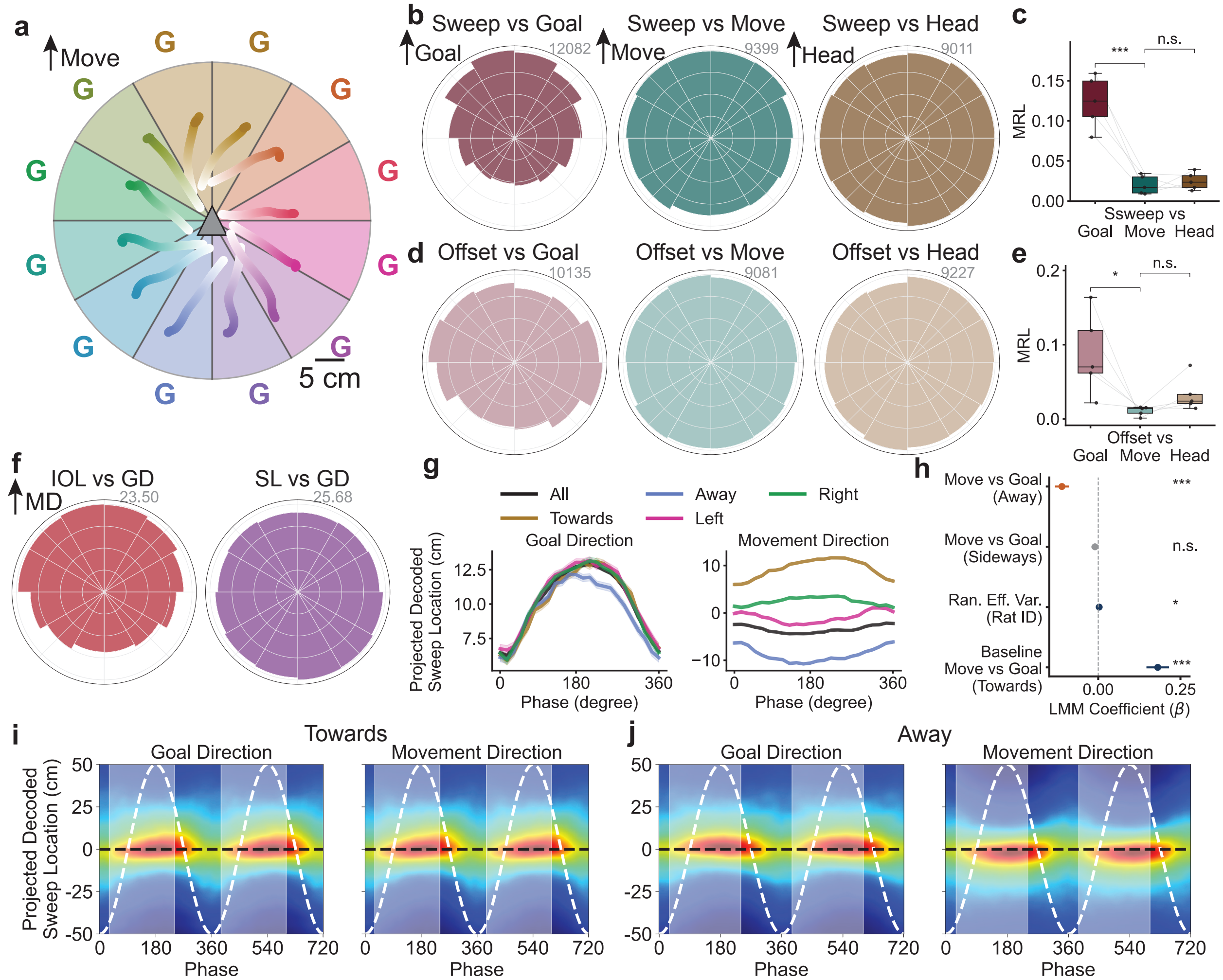

Supplementary Figure S3. **Goal-oriented directional bias in theta sweeps persists given MUA-extracted theta rhythmicity.** Theta cycles were derived from LFP in the main analyses of the Honeycomb maze. Here we show the goal-oriented directional bias persists when theta cycles are calculated from multi-unit activities (MUA; cf. Figure 1, using the same labels). **a.** Median theta sweeps as a function of relative movement direction (upward) to the goal. **b.** Distributions of relative angles between sweep direction and goal direction (left), sweep direction and movement direction (middle), and sweep direction and head direction (right) for all valid theta sweeps (n=106,264 valid theta sweeps in total given MUA-extracted theta oscillation) (one-sided shuffled test with 10,000 shuffles; Sweep vs Goal: p<0.0001; Sweep vs Move: p=0.0037; Sweep vs Head: p<0.0001). **c.** MRL of rat-specific distributions in **b** (two-sided paired t-test, n=5 animals; Goal vs Move: p=0.0001, t=6.7214; Move vs Head: p=0.5184, t=

-0.6755; d.f.=4). **d.** Same as **b**, but for distributions with respect to the initial offset direction (one-sided shuffled test; Offset vs Goal: p<0.0001; Offset vs Move: p=0.1031; Offset vs Head: p<0.0001). **e.** Same as c, but for distributions in **d** (two-sided paired t-tes, n=5 animalst; Goal vs Move: p=0.0007, t=5.3264; Move vs Head: p=0.8574, t= -0.1856; d.f.=4). **f.** Median (over all n=106,624 valid theta sweeps) initial offset distance to the animal (left) and sweep length (right), as a function of relative movement direction (upward) to the goal (upward). **g.** Mean projection of decoded theta sweeps (± 1 s.e.) along the goal- (left) and movement-directions (right), over all periods and movements towards, away from and sideways to the goal. Similar to the observation given LFP-extracted theta cycles, we identified robust goal-oriented directional modulation in theta sweeps. **h.** Fitted LMM coefficients (with 95% confidence interval indicated by the error bar) predicting the cosine similarity between sweep and goal directions of individual theta sweeps (with corresponding 95% confidence interval), given categorical predictors of animal's relative movement direction to the goal (towards, away, and sideways; cf. Extended Data Figure 4l). Moving opposite the goal direction yielded significant decrease in the alignment between sweep and goal directions (β= -0.1105, z= -10.3192, p=0.0008), whereas moving sideways to the yielded little changes (β= -0.0103, z= -0.0215, p=0.0721). **i-j.** Averaged decoding posteriors over all theta sweeps, projected onto corresponding goal- (left) and movement-directions (right), as a function of theta phase, relative to rats' position (black dashed line) when rats are moving towards **(i)** and away from **(j)** the goal. Two theta cycles (white dashed line) are shown. Shaded areas indicate average (over all rats) phase range of "forward-extending" theta sweeps [9,25]. Analysis given either MUA- or LFP-derived theta cycles lead to the same main findings of robust goal-oriented directional modulation of theta sweeps.

Component	Parameter	Value	Description
**Directional Cells Network**	τ_h	10 ms	Time constant
	N_h	100	Number of neurons
	A_h	3.0	Sensory input strength
	σ_h	0.4 radians	Sensory input tuning width
	$\bar{\alpha}_h$	0.4	Theta modulation strength
	A_h^{td}	5.0	Top-down goal-modulated input strength
	γ_h	0.4 radians	Top-down goal-modulated input tuning width
	J_0^h	0.8	Recurrent connection strength
	η_h	0.4 radians	Recurrent connection width
	τ_a^h	100 ms	Adaptation time constant
	m_h	1.0	Adaptation strength
	k_h	1.0	Global inhibition strength
	τ_p	10 ms	Time constant
	N_p	2500	Number of neurons
	A_p	5.0	Spatial input strength
	σ_p	50 cm	Spatial input width
	$\bar{\alpha}_p$	0.5	Theta modulation strength
	k_p	1.0	Global inhibition strength
	g	1000	Population gain factor
	J_0^p	0.01	Recurrent connection strength

**Place Cells Network**	η_p	50 cm	Recurrent connection width
	τ_p^a	100 ms	Adaptation time constant
	m_p	0.6	Adaptation strength
	γ_p	50 cm	Conjunctive grid cell input tuning width
	β_0	0.2	Speed scaling factor
	C_p	0.4	Speed scaling baseline
	w_0	45 cm	Internal direction shift
	w_1	150 cm	Head direction shift
	ν	0.99	Activity threshold
**Simulation Parameters**	dt	1 ms	Time step for numerical integration
	f_θ	10 Hz	Theta frequency
	$Duration$	300 s	Simulation duration

Table S1. **Default model parameters used in continuous attractor network simulations.**
