# Supplementary material for: Hippocampal theta sweeps indicate goal direction during navigation
Source: Nat Neurosci. 2026 Jul 1;29(9):2225–36. doi: 10.1038/s41593-026-02365-2 (PMC13533849; doi:10.1038/s41593-026-02365-2)
Supplement: Supplementary file 3 [file 41593_2026_2365_MOESM3_ESM.pdf]

nature portfolio

Peer Review File

Hippocampal theta sweeps indicate goal direction during navigation

Corresponding Author: Dr Changmin Yu

Version 0:

Reviewer comments:

Reviewer #1

(Comments for the Author)
Summary
In this paper, Yu and colleagues describe how hippocampal theta-cycle ‘sweeps’ are directionally biased towards the location of goals. These sweeps are independent of current the animal’s current locomotor trajectory or head direction, suggesting that the hippocampal network likely generates, within each theta cycle, a temporally brief representation of a future behaviorally relevant location. This representation is in an egocentric goal-directed frame, and thus provides a moment-by-moment substrate for prospective planning. Moreover, they propose a hierarchical continuous attractor model that can account for these sweeps. Broadly, this work nicely describes how theta sweeps may not only provide a forward prediction of where the animal will go next but are actively biased towards goal directions. I found the work compelling and novel, in that it links theta sweeps to goal directions and nicely extends ideas regarding phase precession and sequence coding to relate these phenomena to behaviorally relevant path trajectories. I also found strength in the inclusion of the model, which provides a mechanistic framework for linking the cellular circuits to the theta sweeps. I have some comments however, that should be addressed prior to publication:

More details regarding the task and the goal direction activity should be provided. It was hard for me to determine how stable the goal representation was across different trajectories or whether neurons involved in goal direction vectors were active across multiple goal locations. I realize some of these details are in the supplemental figures and methods but I’d recommend adding more information to the main results as it currently is hard to interpret. Relatedly, it was very challenging to determine how reproducible or stable these observations were – numbers of sweeps, sessions and cells that went into given analyses were either not presented (or I could not find these details in the main results or figure legends). For example, was Figure 1j all cells (~105 per rat, 5 rats) over all (n = ?) sessions and different (n = ?) goal locations? How many theta sweeps were examined in a given session?

I was a little surprised that the goal direction sweeps were less robust in the cheese board maze, which the authors indicate reflects the frequent alignment between movement and goal direction. How critical is this correspondence? In other words, does this phenomenon only occur when movement direction is aligned with goal direction? This also raised the question of how general this observation might be across different types of tasks. Would the authors expect to see such goal-biases in flexible goal conditions – such as a stable versus moving reward site – or vary with novelty? Some discussion on this would be useful for framing the application of the experimental observation and modeling work across behavioral contexts.

Was there equal representations of all positions from the rat to the goal, or were the sweeps biased by neurons that more preferentially represent reward, regardless of the trajectory the rat plans to take to the reward? Clarifying whether reward coding contribute to the decoding would help determine how much of the effect reflects trajectory planning versus it being driven by reward overrepresentation.

Various plots need to provide information about what is plotted (and ideally show individual data points) – as well as include n values (e.g. Figure 1i, 2f, 2h, 2i).

The modeling section could benefit from more discussion on how sensitive the simulated theta sweeps are to specific parameter choices. For example, how dependent is the emergence of goal-directed bias on the strength of the top-down goal input or the firing rate adaptation?

(Remarks on code availability)

Reviewer #2

(Comments for the Author)
This paper reports the exciting and significant finding that forward sweeping trajectories coded by theta sequences indicate goal direction, independently of movement or head direction. The potential significance of this finding is worthy of publication in a widely read journal like Nature Neuroscience. However, the evidence provided to support the main conclusions is not entirely convincing as currently reported. Assuming that the findings are robust, the authors should be able to provide convincing support to increase the impact of their reported findings by clarifying and correcting statistical analyses, as well as providing further details for some of the methodology and results. My specific comments are as follows:
1. A major concern that occurs throughout the paper is that multiple measures (e.g., theta sweeps, replay events, etc.), presumably from the same trials and recording sessions (and thus the same populations of cells) within the same rats, appear to have been treated as independent measures in the statistical tests. Multiple observations of sequences computed from the same population of cells within a rat are not independent values (i.e., they are not free to vary independently from one another). Thus, treating these types of observations as independent measures artificially inflates the degrees of freedom and could lead to biased results. If I am indeed understanding correctly what was done, the statistical analysis should be re-done to account for between-subject variability and within-subject variability. This issue pertains to the comparisons of measures related to theta sequences and replay throughout the paper, and the same point applies to the speed comparisons in Figure S10.
2. Related to the above point, the authors should report the statistics and the degrees of freedom for every statistical comparison, not just the p-values, so that it is clear to readers how the statistical tests were performed.
3. Related to the above points, in the "Statistical analysis" section of the Methods, the authors write, "Most statistical comparisons are on the rat-level, which we report the p-values of paired independent t-tests…" What does "at the rat-level" mean here? It makes it sound as though measures were averaged within each rat, yielding a sample of 1 for each group from each rat. But, I don't think that is what was actually done. Later in the same paragraph, the authors write, "…we use the Mann-Whitney U test and report the statistical significance, as well as the effective size, r, as the significance can be deceiving especially given large sample sizes". What were the sample sizes? The sample sizes for sequence metrics were not provided. The rat sample size (n=5) was not particularly large. In general, the description of how statistics were performed should be clarified. The number of rats, recording sessions, trials, sequences, etc. included in each analysis is unclear, as is the experimental design for the statistical comparisons.
4. How were the examples selected? An unbiased method for selecting examples would make the examples more convincing (e.g., the sequences corresponding to the top 5% of r2 values, or something similar).
5. How was the Bayesian decoder cross-validated? Figure S4a states that the example posterior probabilities were from a held-out validation trajectory, but the Methods do not clearly state whether all decoding was performed on held-out data.
6. The paper uses a large number of non-standard abbreviations (e.g., SD, IOD, etc.). This impairs readability. The authors should consider writing out many of the non-standard abbreviations.
7. In Figure S7, it appears as though the authors conclude that MVLs across different angles differ for homing and foraging, but it appears that they base this conclusion on non-significant p-values for foraging vs. significant p-values for homing. If this is indeed the case, the authors should instead include all of the factors in the same analysis and show a significant interaction between task type (i.e., foraging and homing) and relative angle type (e.g., GD, HD, etc.) to support this conclusion (see Makin TR, Orban de Xivry JJ. Ten common statistical mistakes to watch out for when writing or reviewing a manuscript. Elife. 2019 Oct 9;8:e48175. doi: 10.7554/eLife.48175.).
8. It would be helpful to show example histology of hippocampal recording locations.
9. Regarding the first few sentences in the "Goal-oriented hippocampal replay sequences" section where multiple relevant references are cited, reference 16 (i.e., Zheng et al. 2021) also reported a bias for replay events to terminate at goal locations.
10. On page 14, the authors write, "The top-down goal-oriented directional input, potentially originated in the RSC…" I think a citation is missing here.
11. In Figure S9, panel g, why is the sweep ahead structure not apparent in the GD plot as rats are moving toward the goal?

(Remarks on code availability)

Reviewer #3

(Comments for the Author)
This manuscript presents data and modeling from a task in which rats are forced to take indirect paths to a goal. The authors analyze "theta sweeps" – a rodent phenomenon in which hippocampal place cells rapidly encode sequences of locations. They show that theta sweeps are directed at the location of the goal, even when that goal is not ahead of the animal.

I agree that this is, to date, the cleanest experimental separation of the goal direction from the heading direction, and the main result of the paper is convincing. However, various results in the literature have long pointed at theta sequences being a cognitive rather than motor-related phenomenon. Theta sequences represent both past and future locations and can quickly alternate between choices (many papers from Frank, Redish, and Foster labs, among others). This alternation includes locations behind the animal's head and even around corners of a maze (e.g. Johnson and Redish 2007, Calvin 2024). Theta sweeps include not only the goal but even salient locations that the animal wants to avoid (Dvorak 2018). All

these papers paint a picture of theta sweeps representing salient, behaviorally-relevant, attended locations. The authors certainly overstate the strawman hypothesis of theta sweeps being a movement-related phenomenon, as well as the conceptual novelty of their findings.

A major issue with the paper itself is that the model is highly problematic – in its motivation, presentation, and results. I suspect that the main result of the model is actual a trivial consequence of parameter choices. As currently written, the model is a major part of the manuscript. Another issue is that the paper is exceptionally poorly written. It is riddled with run-on sentences, unclear and obfuscating terminology, sentence fragments, grammatical errors, and typos. For a reviewer, this manuscript was a frustrating, time-sinking struggle to dig through, and I am still pretty sure I understood only a fraction of what the authors wanted to say. This manuscript should not be submitted again without a major re-write of the text.

MAJOR ISSUES

1. Issues with the model

a. It is unclear why the model exhibits strong goal direction tuning but not movement direction tuning. In the model, both of these signals enter through the DC network and, as far as I can tell, are parametrized in a similar way. If that's the case, then the model is goal-tuned simply because the goal input is stronger than the movement input. That's a trivial result. If that's not the reason, then what exactly about the goal input causes it to have such a large effect on place cells?

b. Along these lines, it is generally important that a model not only replicates features of the data, but also *explains* something about what causes the system to work. It is not at all surprising that a complex, multi-layered model with many fine-tuned parameters replicates data, or even predicts something about the data that the authors hadn't previously analyzed. The authors need to show under what conditions their model *fails* to replicate the data. Then, based on comparing successful and unsuccessful regions of the parameter space, they might conclude something about what makes the model work. It doesn't help that the authors provide very little justification, and in some cases omit information, about their parameter choices for the model (see Minor Issues).

c. The authors claim to have two layers of grid cells, one of which is conjunctive and one that is an attractor network. But this is not what they actually simulated! From what I gathered, the "PC network" is simply a layer of place cells that receives goal-biased directional input and input from other place cells. As an excuse for not modeling grid cells, the authors invoke "GPU-memory constraints" (which raises an eyebrow in the era of cloud computing) and "surjective Hebbian plasticity" – a term that they do not explain and that appears nowhere in the literature. The authors either need to model grid cells or very clearly explain why they do not need to do so. Otherwise the text is highly misleading about what the authors actually did.

2. Issues with the text

Although each of these by itself is minor, together these issues create a paper that is very difficult, or impossible, to read and understand. I'm pointing out particularly egregious issues with the text. In my opinion, almost every sentence in the manuscript should be read out loud and reworked until it makes sense to outside readers.
"Recent results have suggested a cognitive…" is a sentence fragment. Who is doing the sampling?
"Cognitive affordance of theta sweeps". What does this mean? Affordance is an unrelated psychological term from Gibson.
"Due to its cyclic nature…" Confusing/grammatically incorrect sentence
"differential directional-modulatory effects" What does this mean?
"Residual sweep direction…" run-on sentence
"To induce goal-modulation…" another run-on sentence
"Given simulated trajectories…" long and very confusing sentence
"simulated theta sweeps revert to forward and left-right alternation" What does it mean for theta sweeps to revert?
"Mechanistically, this is due to the joint effect of concurrent movement of the animal during the course of goal-directed sweeps, and reduced firing rate adaptation due to misalignment of sweep- and movement-directions (compared to goalward movement)." What does "this" refer to? What is "concurrent movement"? What is misalignment and sweep and movement directions, and what does it mean to compare misalignment with goalward movement? How does this misalignment reduce firing rate adaptation (if I understood that logical link correctly)? How does all of this explain "this"?
"Thus the appearance of theta sequences" is not in a clear logical way related to what was said in the preceding sentence.
"However, the goal-directed sweeps here require a population-level description…" What does this entire sentence mean? What does it mean for sweeps to require something? What does it mean for them to cause something?
"In this case, the goal-directed sweeps…" Again, the logic of this sentence is completely unclear.
"We confirmed this prediction empirically…" Unclear sentence.
"replay might reflect spatial trajectories composed of theta sweeps" What does it mean for replay to reflect trajectories? What does it mean for these trajectories to be composed of sweeps?
"towards the goal location and moving closer from the first to second half" Who is doing the moving?
"trajectories reflect the composition of multiple theta sweeps" What does that mean? Later in that sentence, what does it mean for replay to "reflect LTP". What is the logical link between the observation and LTP?
"Complementing fan-tail representations of preferred goal directions…" What does that mean?
"Building upon an existing…" Run-on sentence; unclear what "which" refers to
"potentially reflecting their affordance of plasticity across place cell assemblies" What does this mean?
The tense is constantly mixed up in the paper. The authors sometimes switch between past and present several times per paragraph.
Please correct numerous typos and grammatical issues like is/are confusion. There were too many for me to mark. There is

software these days that will do a good job detecting problems automatically.

MINOR ISSUES

Discussion. “location-independent sense of direction” What results show location independence?
Discussion of mammalian evolution in the last paragraph is very questionable. If anything, a growing body of work suggests highly species-specific processing. See primates (very little spatial coding, if any; very little theta, if any) and bats (place cells, no theta). Perhaps goal-directed nonlocal coding in the hippocampus is a general principle. But the details that the authors refer to here might be specific to the ecological niche of mice and rats. If the authors are referring to the general principles, then they likely did not emerge “early in mammalian evolution”: see results on place cells in birds and fish.
Why do the authors not make circular histograms for head direction tuning? These are mentioned in the text but not shown. Given the emphasis on head direction vs. goal direction, these should be presented more extensively side-by-side.
“Discovered subicular… circuitry” isn’t quite right
Result in Fig. 3g is critical because it’s predicted by the model. But it’s not clear where the result of the model is and how it compares. A better statistical treatment of this effect is needed.
In Eq. 4, shouldn’t 1/N be 1/sqrt(N). Or is 1/N correct here?
The authors should clearly state the number of units used in the network, as well as all the other parameters needed to implement the model.
Eqns. 9 and 13 appear to be missing a threshold operation, otherwise one simply has a quadratic activation function, which would be a very strange choice.
Eq. 13 has a typo in the numeration. I believe it should be g h_j^2
Using brackets to denote Euclidean distance is unusual, since these are usually used for inner products or for expectations. It is better to use norm notation: dist(s_i,s_j) = ||s_i – s_j||
Is modulo 2pi missing in the distance operations?
What is omega_t ?
Fig. 1h campus should be same as (g) not same as (f)
The claim “The DC attractor… driving PC activity bump towards the goal direction” is not true. None of these features are actually present in the model.
If this paper is submitted again, please include line numbers.

(Remarks on code availability)

Version 1:

Reviewer comments:

Reviewer #1

(Comments for the Author)
The authors have thoroughly addressed my reviews and concerns. I recommend publication.

(Remarks on code availability)

Reviewer #2

(Comments for the Author)
The authors have thoroughly addressed my previous concerns.

(Remarks on code availability)

Reviewer #3

(Comments for the Author)
The new version of the manuscript is a substantial improvement from the original and addresses some of my concerns. In particular, the manuscript is now more readable, softens some of the most egregious claims, and is more transparent about the model implementation.

Unfortunately some of my central concerns about the model – currently a major component of the paper – remain, in part because they are now confirmed by the authors themselves. The model is not truly a mechanistic circuit model, since the grid cell layer is not actually modeled. The authors have truthfully removed those claims. Its explanatory power about the circuit is therefore extremely limited if not nonexistent.

More importantly, the authors have now admitted that the central claim of the model – that there is goal tuning in neural activity – is a trivial consequence of a strong goal input into the network. Without this central claim, and without a clear mechanistic link to biological circuits, I struggle to see what the contribution of the model is. As I wrote in my original review, demonstrating that some arbitrary model replicates some features of data is a very weak scientific contribution. There could

be dozens of broad classes of models that replicate the same features of the data. There needs to be some explanation of *what* causes the model to work and, conversely, what could cause other plausible models to fail.

I am not convinced by the authors’ sensitivity analysis in the revision, which frankly feels like a cosmetic change to try appeasing a critical reviewer. The analysis is one-dimensional, changing one parameter at a time, and it shows that the conclusions are not horribly dependent on the exact values of the parameters. But it still fails to provide any explanatory power to the model. What exactly have we learned about what causes models to work or not work – that is a litmus test of modeling that the manuscript fails.

It would help if some predictions of the model were truly unexpected or demonstrated to be difficult to obtain in computational models. But unfortunately most of the predictions seem to be very expected consequences of model construction (e.g. that stronger theta modulation produces longer sweeps, given that theta modulation in the model is what produces the sweeps). The only somewhat interesting prediction is phase precession vs. procession depending on the movement direction. But, again, without a strong mechanistic explanation I’m not sure that the model is viable simply based on this one prediction.

(Remarks on code availability)

Open Access This Peer Review File is licensed under a Creative Commons Attribution 4.0 International License, which permits use, sharing, adaptation, distribution and reproduction in any medium or format, as long as you give appropriate credit to the original author(s) and the source, provide a link to the Creative Commons license, and indicate if changes were made.
In cases where reviewers are anonymous, credit should be given to 'Anonymous Referee' and the source.
The images or other third party material in this Peer Review File are included in the article’s Creative Commons license, unless indicated otherwise in a credit line to the material. If material is not included in the article’s Creative Commons license and your intended use is not permitted by statutory regulation or exceeds the permitted use, you will need to obtain permission directly from the copyright holder.
To view a copy of this license, visit https://creativecommons.org/licenses/by/4.0/

We thank all reviewers for their constructive comments and overall recognition of the strengths of the manuscript. Below we address each reviewer's comments individually, with both direct response and reference to updated text/figures in the updated manuscript.

Reviewer 1 (Comments for the Author)

In this paper, Yu and colleagues describe how hippocampal theta-cycle 'sweeps' are directionally biased towards the location of goals. These sweeps are independent of current the animal's current locomotor trajectory or head direction, suggesting that the hippocampal network likely generates, within each theta cycle, a temporally brief representation of a future behaviorally relevant location. This representation is in an egocentric goal-directed frame, and thus provides a moment-by-moment substrate for prospective planning. Moreover, they propose a hierarchical continuous attractor model that can account for these sweeps. Broadly, this work nicely describes how theta sweeps may not only provide a forward prediction of where the animal will go next but are actively biased towards goal directions. I found the work compelling and novel, in that it links theta sweeps to goal directions and nicely extends ideas regarding phase precession and sequence coding to relate these phenomena to behaviorally relevant path trajectories. I also found strength in the inclusion of the model, which provides a mechanistic framework for linking the cellular circuits to the theta sweeps. I have some comments however, that should be addressed prior to publication:

> We are grateful to the reviewer for their recognition of the validity and novelty of the manuscript, and for providing constructive comments. Your suggestions helped us to improve the clarity and impact of the manuscript substantially.

More details regarding the task and the goal direction activity should be provided. It was hard for me to determine how stable the goal representation was across different trajectories or whether neurons involved in goal direction vectors were active across multiple goal locations. I realize some of these details are in the supplemental figures and methods but I'd recommend adding more information to the main results as it currently is hard to interpret. Relatedly, it was very challenging to determine how reproducible or stable these observations were – numbers of sweeps, sessions and cells that went into given analyses were either not presented (or I could not find these details in the main results or figure legends). For example, was Figure 1j all cells (~105 per rat, 5 rats) over all (n = ?) sessions and different (n = ?) goal locations? How many theta sweeps were examined in a given session?

> Thank you for the specific suggestion for improving clarity. Regarding the specific question on the number of sweeps, sessions, and cells that went into each analysis, we previously included such information in the supplementary, and have now also included this in the main text (honeycomb maze: page 5 line 1-3; open-field: page 9 line 4-5, page 9 line 9-12).
>
> Honeycomb maze:
>
> - In total, 137,651 theta sweeps were identified from 5 animals (25,574; 27,696; 16,070; 39,993; 28,813 for Rats 1-5, respectively; one recording session per animal with constant session-specific reward location).
>
> Cheeseboard maze:

- To validate our findings in settings with more recorded place cells (151.75±59.25 putative place cells per rat, from 4 rats)…
- Theta sweeps were identified with similar criteria as described for the Honeycomb maze (minimum speed threshold increased to 5 cm/s as rats run faster on this maze), leading to a total of 85,210 theta sweeps over two recording sessions (Rat 1: 6,180, 13,985; Rat 2: 10,395, 12,299; Rat 3: 12,375, 11,814; Rat 4: 9,772, 8,410).

I was a little surprised that the goal direction sweeps were less robust in the cheese board maze, which the authors indicate reflects the frequent alignment between movement and goal direction. How critical is this correspondence? In other words, does this phenomenon only occur when movement direction is aligned with goal direction? This also raised the question of how general this observation might be across different types of tasks. Would the authors expect to see such goal-biases in flexible goal conditions – such as a stable versus moving reward site – or vary with novelty? Some discussion on this would be useful for framing the application of the experimental observation and modeling work across behavioral contexts.

We thank the reviewer for raising the important point on dependence of goal bias in theta sweeps on movement-goal alignment. In the cheeseboard environment, the reason we see less robust goal-oriented directional bias overall was mainly due to the fact that rats did not know the distant reward location during foraging trials. We actually observed similar levels of concentration (quantified through MRL) in distributions of relative angles between sweep and goal directions for sweeps in the honeycomb maze and in *homing trials* in the cheeseboard maze (Figure 1g-h, cf. Figure 4c-d in the updated manuscript). We did, however, find that sweep directions are most strongly goal-oriented when movement and goal directions align in the Honeycomb maze (page 6 line 3-7 and Extended Data Figure 4g-k).

- Combining the goal-directionality of the theta sweeps with the (weaker) movement-directionality showed a significantly tighter concentration around the goal direction when animals were moving towards the goal compared to moving away from or at right angles to the goal (Extended Data Figure 4j), both at the level of the individual rat (Extended Data Figure 4k) and of the individual sweep (based on LMM analyses as above; Extended Data Figure 4l).

Was there equal representations of all positions from the rat to the goal, or were the sweeps biased by neurons that more preferentially represent reward, regardless of the trajectory the rat plans to take to the reward? Clarifying whether reward coding contribute to the decoding would help determine how much of the effect reflects trajectory planning versus it being driven by reward overrepresentation.

Given the aggregated population rate maps of all pyramidal neurons (Extended Data Figure 3a), we confirmed that there was no over-representation of the goal location. We have included the following text in the main text for clarification (page 6 line 13-14).

- First, we demonstrated that aggregated population rate maps did not over-represent goal locations (Extended Data Figure 2c).

We further noted the existence of neurons with significant egocentric goal-directional tuning (as reported by Ormond and O'Keefe, 2022) and computed the firing phase of these neurons. We verified that there is no systematic pattern in their spiking phase, hence precluding the possibility that goal-directed sweeps were driven by these neurons firing at specific theta phases (e.g. biasing sweeps by firing at later but not earlier phases). We have included these new analyses in the main text (page 6 line 16-24, Extended Data Figure 5).

- If goal-directed sweeps arose from selective amplification of goal-tuned cells, these cells should exhibit elevated firing rates during goal-directed sweeps (i.e. sweep direction within 45° of the goal direction) compared to non-goal-directed sweeps, and particularly at late phases when sweeps are at their maximum extent. Contrary to this hypothesis, the firing rate ratio between goal-directed and non-goal-directed sweeps remained stable at approximately 1.0 across all theta phases for cells with egocentric goal-direction tuning, and for all remaining cells (Extended Data Figure 5b-h). Collectively, these results demonstrate that goal-directed sweeps reflect coordinated population dynamics, rather than goal-related firing rate modulations in individual neurons.

We also found that, while reward-related firing rate modulation could not cause the goal-directed theta sweeps, the goal directed theta sweeps could cause firing rate modulation by egocentric goal direction, via an interaction with firing rate adaptation (a prediction of the model, also validated in data; page 12 line 6-9).

- The interaction between firing rate adaptation and movement direction causes greater firing during goal-directed sweeps to goals ahead of the movement direction, because they involve place cells that have not fired recently. We also found the same modulation of firing intensity given alignment between movement and goal direction in the data (Extended Data Figure 4i).

Various plots need to provide information about what is plotted (and ideally show individual data points) – as well as include n values (e.g. Figure 1i, 2f, 2h, 2i).

We thank the reviewer for the suggestion. Our figures include individual data points where possible (and we omitted showing all data points if analysis was performed on the individual sweep-level due to extended rendering time). Regarding statistical tests, in addition to p-values, we have additionally included the test statistics and degree of freedom (or sample size for non-parametric tests) in the updated manuscript.

The modeling section could benefit from more discussion on how sensitive the simulated theta sweeps are to specific parameter choices. For example, how dependent is the emergence of goal-directed bias on the strength of the top-down goal input or the firing rate adaptation?

We thank the reviewer for raising the important point. We have included sensitivity analysis results for key model parameters (Extended Data Figure 9j; page 11 line 15-18). We found that the goal-directed bias in simulated theta sweeps was robust to parameter changes, and was primarily driven by the top-down goal-directed input, but that there is also an important interaction between the top-down input and the intrinsic dynamics of place cells. This interaction causes the model to make a number of non-trivial predictions, which we verified in the real data. These predictions include: sweep length variations dependent on theta modulations (Extended

Data Figure 9c, Figure 2c; page 11 line 23-26) and alignment between goal and movement directions (Figure 2a, Extended Data Figure 9b; page 11 line 26 - page 12 line 5), goal-oriented anticipatory coding and egocentric directional tuning in place cells (Figure 6; page 12 line 6-21), goal-dependent phase coding (Figure 7; page 12 line 23 - page 13 line 10).

- To ensure the robustness of our findings, we performed a systematic sensitivity analysis by independently varying key model parameters (Extended Data Figure 9j). Goal-directed modulation remained robust across a wide range of parameter values.
- In addition to the dynamical input from the grid system, the theta modulation and firing rate adaptation in the place cells contribute to the occurrence of theta sweeps, with increasing theta modulation causing longer sweeps[19,26], successfully reproducing the positive scaling of sweep lengths with theta power (Extended Data Figure 9c; cf. Figure 2c).
- The effect of firing rate adaptation interacts with movement direction in interesting ways. Place cells with firing fields behind the rat will have fired more recently than those with fields in front of it and therefore exhibit greater firing rate adaptation. This biases place cell activity in the forward direction from the rat's location, causing the initial offset of goal-directed theta sweeps in the forward movement direction, as seen in the data (Extended Data Figure 9b; cf. Figure 2a).
- The interaction between firing rate adaptation and movement direction causes greater firing during goal-directed sweeps to goals ahead of the movement direction, because they involve place cells that have not fired recently. We also found the same modulation of firing intensity given alignment between movement and goal direction in the data (Extended Data Figure 4i). This predicts that, during rotational scanning behaviour, place cells will fire more before the head direction reaches the goal direction (when the goal is ahead of the rotational movement) than after it passes the goal direction (Figure 6a). We verified the presence of this rotational goal-anticipatory firing in the data (Figure 6b-c). The modulation of firing rates by the angle between goal- and movement-directions (greater firing when they align) creates a modulation of the firing rates of individual place cells by the angle between head- and goal-directions. This tuning will tend to be peaked when goal and head direction align, can peak at other angles according to the precise sampling of movement and head directions in the corresponding place fields (e.g., 90° if predominantly sampled during clockwise or counterclockwise rotational scanning). This provides a potential mechanism for place cells in the Honeycomb Maze to show a convergent egocentric directional bias towards specific locations (convergence sinks or ConSinks) which cluster around the goal location[17]. Indeed, without any parameter tuning, many simulated place cells exhibited ConSink-like egocentric directional tuning (Figure 6d-e), with ConSinks that cluster around the goal location (Figure 6f).
- In rodents running on linear tracks or open fields, place cell firing exhibits "theta phase precession", in which place cells fire at late phases of the theta rhythm when their firing field is ahead of the animal and at successively earlier phases as the animal moves through the field[20], consistent with a higher intrinsic firing frequency than the LFP theta[20]. Thus, population theta sweeps are thought to be consistent with single cell theta phase precession

(i.e., place cells active at early phases having firing fields behind the rat and those active at later phases having firing fields ahead of the rat)[2,3], with the additional requirement of brief experience of the environment[27] and CA3 activity[28]. However, the goal-directed theta sweeps presented here predict theta phase precession when the animal is moving towards the goal, and theta phase procession when the animal moves away from the goal, as shown by simulations of the model (Figure 7a-c). We confirmed this prediction empirically, finding theta phase precession and procession with respect to distance travelled towards or away from the goal, respectively (Figure 7d-e). Thus, in this situation, in which theta sweeps do not simply propagate forwards, single cell phase coding appears to reflect the population dynamics rather than vice versa.

Reviewer 2 (Comments for the Author)

This paper reports the exciting and significant finding that forward sweeping trajectories coded by theta sequences indicate goal direction, independently of movement or head direction. The potential significance of this finding is worthy of publication in a widely read journal like Nature Neuroscience. However, the evidence provided to support the main conclusions is not entirely convincing as currently reported. Assuming that the findings are robust, the authors should be able to provide convincing support to increase the impact of their reported findings by clarifying and correcting statistical analyses, as well as providing further details for some of the methodology and results. My specific comments are as follows:

We are grateful to the reviewer for their recognition of the significance of the manuscript, and for their instructive comments. Their comments have helped us to make clear the robustness of the results.

1. A major concern that occurs throughout the paper is that multiple measures (e.g., theta sweeps, replay events, etc.), presumably from the same trials and recording sessions (and thus the same populations of cells) within the same rats, appear to have been treated as independent measures in the statistical tests. Multiple observations of sequences computed from the same population of cells within a rat are not independent values (i.e., they are not free to vary independently from one another). Thus, treating these types of observations as independent measures artificially inflates the degrees of freedom and could lead to biased results. If I am indeed understanding correctly what was done, the statistical analysis should be re-done to account for between-subject variability and within-subject variability. This issue pertains to the comparisons of measures related to theta sequences and replay throughout the paper, and the same point applies to the speed comparisons in Figure S10.

We thank the reviewer for raising this important point. We agree that the dependence of multiple observations of sequential events (theta sweeps, replay trajectories, and related behavioural data) derived from the same simultaneously recorded neuronal population within a rat/session should be included in statistical analysis. Treating these as independent would inflate degrees of freedom. We would like to clarify that, in the original submission, our statistical tests were not performed at the individual sweep/event level. Instead, wherever possible, we firstly aggregated within each rat (or session for open-field navigation) so that all sweeps/events from a given rat/session contributed to a single summary statistic (e.g., mean resultant vector length). We then performed statistical comparisons across rats/sessions between conditions using these summary

statistics. Consequently, the nominal degrees of freedom in these tests were typically small — often on the order of the number of animals (e.g., d.f. = 4 for n = 5 rats in the Honeycomb maze and d.f. = 7 for n = 8 sessions in the cheeseboard environment) — rather than the number of individual sweeps/events. In order words, the original statistical tests were designed to avoid pseudo-replication by using rat/session as the unit of inference.

That said, we appreciate that aggregation within animals can (i) obscure within-rat variability and (ii) limit power. Therefore, in the revised manuscript, we additionally implemented linear mixed-effects models (LMMs) on the individual sweep/event level, with explicitly hierarchical structure to account for the nested data structure. For example, complementing the rat-level MRL comparisons (e.g., Figure 1g, h in the updated manuscript), we fitted LMMs for predicting cosine similarity between each individual sweep direction and reference directions given fixed effects corresponding to the predictors of interests (e.g., reference direction type) and random effects that account for the nested structure of the data. The LMM analysis used all available sweeps/events whilst ensuring that inference was driven by inter-animal/session variability rather than inflated by within-session replication. Importantly, the sweep/event-level analyses confirmed the conclusions obtained from the rat-session-level aggregated tests, and we have retained the aggregated (rat/session-level) analyses in the revised manuscript for transparency and to preserve the original unit-of-inference interpretation. We now report both types of comparisons, which yield convergent results. Finally, regarding the reviewer's note that the same concern applies to the speed comparisons (currently Extended Data Figure 6 in the updated manuscript), we have treated these measures analogously: speed-related quantities are summarised at the rat/session level for the primary comparisons, and we additionally report mixed-effects analyses where repeated measurements within rat/session are used (in captions of Extended Data Figure 6).

2. Related to the above point, the authors should report the statistics and the degrees of freedom for every statistical comparison, not just the p-values, so that it is clear to readers how the statistical tests were performed.

We thank the reviewer for the suggestion. We have now included, in addition to p-values, the test statistic and degree of freedom (or sample size for non-parametric tests) for all tests in the updated manuscript.

3. Related to the above points, in the "Statistical analysis" section of the Methods, the authors write, "Most statistical comparisons are on the rat-level, which we report the p-values of paired independent t-tests…" What does "at the rat-level" mean here? It makes it sound as though measures were averaged within each rat, yielding a sample of 1 for each group from each rat. But, I don't think that is what was actually done. Later in the same paragraph, the authors write, "…we use the Mann-Whitney U test and report the statistical significance, as well as the effective size, r, as the significance can be deceiving especially given large sample sizes". What were the sample sizes? The sample sizes for sequence metrics were not provided. The rat sample size (n=5) was not particularly large. In general, the description of how statistics were performed should be clarified. The number of rats, recording sessions, trials, sequences, etc. included in each analysis is unclear, as is the experimental design for the statistical comparisons.

We thank the reviewer for raising the points on including details about data and test results. All sample sizes (and degree of freedom where appropriate), and relevant details of the data are now

included in the relevant main text and figure legends. Regarding the rat-level tests, please refer to the above response to the reviewer's first comment for detailed description. We have additionally updated the "statistical analysis" subsection in the Methods for more concrete description of the tests we used both at the rat-level and at the individual event-level (with LMM analysis; page 37 line 12 - page 39 line 3).

- Statistical Analysis

Rat-level statistical tests. To avoid pseudo-replication, our preliminary analyses treated the rat (or the recording session in the Cheeseboard maze) as the unit of inference. Specifically, for each rat/session, we firstly aggregated across all sweeps or replay events to obtain a single summary statistic (e.g., MRL of sweep-direction distributions, or mean sweep lengths). We then compared these rat/session-level summary values between conditions using two-sided tests (or one-sided test when there is strong prior hypothesis about the relationship between the two groups under comparison). We used student-t tests as the default statistical test. Some comparisons violate assumptions under t-tests, e.g., the normality assumption for speed/angular-speed comparison due to right-skewness, and unequal variances for comparison between theta sequences and replay sequences. In these cases, we used nonparametric tests such as Wilcoxon signed-rank test for paired comparisons and Mann-Whitney U test for unpaired comparisons. Following the general wisdom, $r<0.2$ is considered a minor effect size (Figure 8e). For all rat-level tests, we report p-values, test-statistics, and degree of freedom (or sample size for non-parametric tests).

Individual-event-level tests. In addition to the rat-level comparison, we also performed linear mixed model (LMM) analysis[57] to make full use of the data at individual event-level whilst accounting for hierarchical dependence. For instance, multiple theta sweeps within a session are derived from spatial decoding of the same simultaneously recorded neuronal population, and sessions are nested within animals; consequently, treating sweeps as independent observations would inflate degrees of freedom. LMMs allow fixed effects of experimentally defined predictors (e.g., trial type, alignment between movement and goal direction, or reference-direction category) to be estimated while modelling correlation structure induced by repeated measurements within the same animal and/or session via random effects. For each dependent variable, y_i (e.g., cosine similarity between sweep or replay direction and goal direction; sweep length; movement speed within each theta cycle), we fit linear models of the following form**.**

$$y_i = \beta_0 + \sum_{k=1}^{p} \beta_k u_{ik} + \sum_{m=1}^{q} b_{g(i),m} z_{im} + \epsilon_i,$$

where u_{ik} is the k-th fixed-effect predictor (e.g., reference direction or trial type) for the i-th observation (e.g., animal or session nested within animal), with coefficient β_k and corresponding grouping $g(i)$, and z_{im} are the corresponding random-effect variables with group-specific coefficients $b_{g(i),m}$. Random effects are assumed to be *a priori* Gaussian-distributed, and noise terms are assumed independent conditioned on the random effects ($\epsilon_i \sim \mathcal{N}(0, \sigma$^2). In some cases, we include additional second-order interaction terms between

existing fixed-effect predictors with corresponding coefficients (e.g., trial type × reference direction interaction in the Cheeseboard maze). Fixed-effect significance is assessed using Wald t-tests, and we report fixed effect estimates, test statistics, and p-values. Note that in testing for alignment strength between sequence directions (sweep or replay) and relative directions (head/movement/goal; e.g., Extended Figure 4d, e, l; Extended Data Figure 5d, g; Extended Data Figure 6b; Extended Data Figure 7i; Extended Data Figure 8m; Extended Data Figure 10; Supplementary Figure S2h), we chose to use cosine similarity of the relative angles as the dependent variable, transforming circular distances into a bounded linear scale [-1, 1] where the assumption of Gaussian noise is more likely to hold. Importantly, assuming that mean angular difference is sufficiently close to 0, MRL is the population-level summary of cosine similarities at the individual-level, hence connecting the LMM analysis and the population analysis (at the rat-level).

We thank the reviewer for pointing out our omission of the selection criterion of samples for visual demonstrations. We have now included the selected criterion in Methods (page 34 line 13-16).

- Sample theta sweeps for visual demonstrations (Figure 1d) were selected randomly from the pool of strongly goal-oriented theta sweeps (relative angle between sweep and goal directions within $[-30°, 30°]$), separately curated for periods when animals were moving towards, away from, and sideways to the goal direction.

5. How was the Bayesian decoder cross-validated? Figure S4a states that the example posterior probabilities were from a held-out validation trajectory, but the Methods do not clearly state whether all decoding was performed on held-out data.

The Bayesian decoder was cross-validated on a trial-by-trial basis. Specifically, decoding accuracy was validated on all valid data from a held-out trial, given spatial ratemaps computed from all other trials in the session (page 33 line 4-6; Extended Data Figure 3).

- Decoding accuracy (given temporal binning of 250 ms) was cross-validated by computing the decoding error for all valid periods within each trial, using rate maps constructed from all remaining trials.

6. The paper uses a large number of non-standard abbreviations (e.g., SD, IOD, etc.). This impairs readability. The authors should consider writing out many of the non-standard abbreviations.

We thank the reviewer for the formatting suggestion. We have significantly decreased the frequency of non-standard abbreviations in both figures and texts in the updated manuscript.

7. In Figure S7, it appears as though the authors conclude that MVLs across different angles differ for homing and foraging, but it appears that they base this conclusion on non-significant p-values for foraging vs. significant p-values for homing. If this is indeed the case, the authors should instead include all of the factors in the same analysis and show a significant interaction between task type (i.e., foraging and homing) and relative angle type (e.g., GD, HD, etc.) to support this conclusion (see Makin TR, Orban de Xivry JJ. Ten common statistical mistakes to watch out for when writing or reviewing a manuscript. Elife. 2019 Oct 9;8:e48175. doi: 10.7554/eLife.48175.).

We thank the reviewer for this insightful question. Similar to the LMM analysis described above, we applied LMM analysis to identify significant factors that contributed to changes in alignment between sweep and various relative directions (head/movement/goal) in open-field navigation, given fixed effect factors of relative direction type and trial type (homing or foraging), as well as their interaction terms, taking into account the nested structure of the data. We indeed found significantly negative interaction between movement/head relative direction and foraging trial type, consistent with our original findings (page 9 line 20 - page 10 line 12; Figure 4, Extended Data Figure 8).

- To investigate whether goal-bias in theta sweep directions is dependent on task demand at the level of the individual sweep, we applied the LMM analysis described above, now with trial type as an additional factor (categorical predictors: homing and foraging), as well as the interaction terms between trial types and reference directions (Extended Data Figure 8m). We found that sweeps were significantly goal directed even during foraging trials (baseline intercept: $\beta = 0.0694$, z=4.3561, p=0.0002), however the effect of movement-alignment was statistically indistinguishable from goal alignment ($\beta = -0.0033$, z=-0.7635, p=0.4460), suggesting that sweep directions reflect the coupling between movement- and goal-directions during random foraging. Goal-directed navigation during homing trials significantly strengthened the alignment between sweep and goal directions (effect of homing trial type: $\beta = 0.0872$, z=15.9349, p< 0.0001), effectively doubling the magnitude of the goal bias during foraging. Importantly, while sweeps tracked both movement and goal directions equally during foraging, sweeps were selectively decoupled from movement direction during homing trials, evidenced by the negative interaction between homing and movement direction ($\beta = -0.1018$, z=-13.0906, p<0.0001). Finally, we modelled individual recording sessions (each characterised by a distinct goal location during homing trials) rather than rat identity as random effects. We found no modulatory effect on alignment between sweep and corresponding goal directions arising from session-specific reward locations (session-ID: $\beta = -0.0017$, z=1.2146, p=0.2240), similarly to the sweep bias toward the current rather than previous goal location following reward-switch trials on the Honeycomb maze.

8. It would be helpful to show example histology of hippocampal recording locations.

We thank the reviewer for the suggestion. We have now included example histology figures in supplementary Extended Data Figure 2b.

9. Regarding the first few sentences in the "Goal-oriented hippocampal replay sequences" section where multiple relevant references are cited, reference 16 (i.e., Zheng et al. 2021) also reported a bias for replay events to terminate at goal locations.

We thank the reviewer for correctly noting our previous omission. We have now updated the draft with corrected references (page 13 line 14, note that currently reference 13 is Zheng et al., 2021).

- The decoded trajectories in open-field arenas often exhibit a goal-directed bias[13,18]…

10. On page 14, the authors write, "The top-down goal-oriented directional input, potentially originated in the RSC…" I think a citation is missing here.

We thank the reviewer for noting this omission. We have now included our existing citations on RSC's contribution to egocentric and allocentric encoding of goal locations (Vedder et al., 2017, Miller et al., 2019) at the specified location (page 42 line 4), and included citations of another more recent studies from Tiago Branco's lab on RSC encoding of behaviourally salient locations (Campagner et al., 2023).

- The top-down goal-oriented directional input, potentially originated in the RSC[39,40,62]…

11. In Figure S9, panel g, why is the sweep ahead structure not apparent in the GD plot as rats are moving toward the goal?

Firstly, please accept our apologies in that the ordering of original Figure S9g and h was incorrect, with the h panel being the projected posteriors when movement and goal directions align, and g panel being the projection when rats were moving away from the goal (note the right panels in g and h). We are now presenting the figures in correct ordering, and increasing the y-axis range to help with the visualisation. This shows that (currently Supplementary Figure S3i and j; give MUA-extracted theta) decoded posterior preferentially swept forward along the goal direction, regardless of the movement direction, but sweeps appeared more forward-extending when rats were moving towards the goal. This observation is coherent with our findings that despite sweep directions being predominantly goal-oriented, we still observed significant, albeit weaker, movement modulations (Extended Data Figure 4j-l; given LFP-extracted theta).

Reviewer 3 (Comments for the Author)

This manuscript presents data and modeling from a task in which rats are forced to take indirect paths to a goal. The authors analyze "theta sweeps" – a rodent phenomenon in which hippocampal place cells rapidly encode sequences of locations. They show that theta sweeps are directed at the location of the goal, even when that goal is not ahead of the animal.

I agree that this is, to date, the cleanest experimental separation of the goal direction from the heading direction, and the main result of the paper is convincing. However, various results in the literature have long pointed at theta sequences being a cognitive rather than motor-related phenomenon. Theta sequences represent both past and future locations and can quickly alternate between choices (many papers from Frank, Redish, and Foster labs, among others). This alternation includes locations behind the animal's head and even around corners of a maze (e.g. Johnson and Redish 2007, Calvin 2024). Theta sweeps include not only the goal but even salient locations that the animal wants to avoid (Dvorak 2018). All these papers paint a picture of theta sweeps representing salient, behaviorally-relevant, attended locations. The authors certainly overstate the strawman hypothesis of theta sweeps being a movement-related phenomenon, as well as the conceptual novelty of their findings.

We thank the reviewer for recognising the soundness of our findings, and for their thoughtful assessment. Regarding the conceptual novelty of the current study, we thank the reviewer for this perspective and for suggesting relevant additional references. We agree that a substantial body of work already suggests a cognitive interpretation of theta sweeps, including the relationship between sweep length and goal distance which we referred to (although the majority of reports show relatively automatic forward or left/right alternation, independent of goal or choice). We

have therefore revisited the framing to more explicitly acknowledge and cite this prior literature, and to focus on the central advance of our work, which is to provide a definitive account that theta sweeps encode allocentric goal-vectorial information that cannot be explained by confounding alternatives (such as instantaneous motor variables or decoding constrained to available paths on narrow-armed mazes), which is difficult in settings where heading, movement, and goal directions are strongly inter-related. We further link this goal-directional bias to subsequent navigational success and provide a mechanistic model that reproduces the effect and generates non-trivial model-based predictions that we confirm empirically. We have revised statements of novelty throughout the manuscript to emphasise this narrower but stronger definition of our contribution in both the Introduction (page 3 line 8-14) and Discussion sections (page 14 line 15-18). Note that references 10 and 11 are Calvin et al., 2025 and Dvorek et al., 2018, respectively.

- Recent results have suggested a cognitive role for theta sweeps. They sample potential paths ahead of the animal, sometimes alternating to both sides of a choice point[6–8] or of the movement direction in open fields[9], are modulated by locations to avoid, and extend farther when running faster[8] or towards more distant[12] or better-learned[13] reward locations. However, it remains unclear whether theta sweeps simply sample potential movement trajectories, or whether they embody a true allocentric vector towards places of interest, independent of the animal's current heading or available paths, consistent with the operation of a cognitive map[1,14,15].
- While previous studies have shown that theta sweeps can over-represent salient locations on narrow-armed mazes, such as aversive zones[10,11], we have demonstrated that they represent a true allocentric goal-direction vector, decoupled from the animal's movement or heading.

A major issue with the paper itself is that the model is highly problematic – in its motivation, presentation, and results. I suspect that the main result of the model is actual a trivial consequence of parameter choices. As currently written, the model is a major part of the manuscript. Another issue is that the paper is exceptionally poorly written. It is riddled with run-on sentences, unclear and obfuscating terminology, sentence fragments, grammatical errors, and typos. For a reviewer, this manuscript was a frustrating, time-sinking struggle to dig through, and I am still pretty sure I understood only a fraction of what the authors wanted to say. This manuscript should not be submitted again without a major re-write of the text.

We thank the reviewer for their careful review of the paper. We have extensively rewritten the text in responses to each specific comment below.

MAJOR ISSUES

1. Issues with the model

a. It is unclear why the model exhibits strong goal direction tuning but not movement direction tuning. In the model, both of these signals enter through the DC network and, as far as I can tell, are parametrized in a similar way. If that's the case, then the model is goal-tuned simply because the goal input is stronger

than the movement input. That's a trivial result. If that's not the reason, then what exactly about the goal input causes it to have such a large effect on place cells?

Following suggestions from both reviewer 1 and 3, we performed systematic sensitivity analyses for testing how goal-directed bias in simulated sweeps varies as parameters change (Extended Data Figure 9j). Crucially, the emergence of goal-directed sweeps does reflect the presence of a dominant goal-direction input to the directional-cell (DC) ring attractor, with the network returning to forward and left/right alternating sweeps in its absence. Across wide one-dimensional perturbations of intrinsic parameters, the relative alignment between simulated sweep direction and goal direction remains robust (Extended Data Figure 9j), indicating that goal bias does not rely on finely tuned parameter settings (page 11 line 15-18). More importantly, despite not being explicitly built into the model design, our model made a number of non-trivial predictions of both sweep characteristics and single-cell firing properties, including sweep length variations dependent on theta modulation (Extended Data Figure 9c, Figure 2c; page 11 line 23-26) and alignment between goal and movement directions (Figure 2a, Extended Data Figure 9b; page 11 line 26 - page 12 line 5), goal-oriented anticipatory coding and egocentric directional tuning in place cells (Figure 6; page 12 line 6-21), goal-dependent phase coding (Figure 7; page 12 line 23 - page 13 line 10). All these predictions are borne out of the interplay between top-down goal-directed inputs and intrinsic dynamics of the networks.

- To ensure the robustness of our findings, we performed a systematic sensitivity analysis by independently varying key model parameters (Extended Data Figure 9j). Goal-directed modulation remained robust across a wide range of parameter values.
- In addition to the dynamical input from the grid system, the theta modulation and firing rate adaptation in the place cells contribute to the occurrence of theta sweeps, with increasing theta modulation causing longer sweeps[19,26], successfully reproducing the positive scaling of sweep lengths with theta power (Extended Data Figure 9c; cf. Figure 2c).
- The effect of firing rate adaptation interacts with movement direction in interesting ways. Place cells with firing fields behind the rat will have fired more recently than those with fields in front of it and therefore exhibit greater firing rate adaptation. This biases place cell activity in the forward direction from the rat's location, causing the initial offset of goal-directed theta sweeps in the forward movement direction, as seen in the data (Extended Data Figure 9b; cf. Figure 2a).
- The interaction between firing rate adaptation and movement direction causes greater firing during goal-directed sweeps to goals ahead of the movement direction, because they involve place cells that have not fired recently. We also found the same modulation of firing intensity given alignment between movement and goal direction in the data (Extended Data Figure 4i). This predicts that, during rotational scanning behaviour, place cells will fire more before the head direction reaches the goal direction (when the goal is ahead of the rotational movement) than after it passes the goal direction (Figure 6a). We verified the presence of this rotational goal-anticipatory firing in the data (Figure 6b-c). The modulation of firing rates by the angle between goal- and movement-directions (greater firing when they align) creates a modulation of the firing rates of individual place cells by the angle between head- and goal-directions.

This tuning will tend to be peaked when goal and head direction align, can peak at other angles according to the precise sampling of movement and head directions in the corresponding place fields (e.g., 90° if predominantly sampled during clockwise or counterclockwise rotational scanning). This provides a potential mechanism for place cells in the Honeycomb Maze to show a convergent egocentric directional bias towards specific locations (convergence sinks or ConSinks) which cluster around the goal location[17]. Indeed, without any parameter tuning, many simulated place cells exhibited ConSink-like egocentric directional tuning (Figure 6d-e), with ConSinks that cluster around the goal location (Figure 6f).

- In rodents running on linear tracks or open fields, place cell firing exhibits "theta phase precession", in which place cells fire at late phases of the theta rhythm when their firing field is ahead of the animal and at successively earlier phases as the animal moves through the field[20], consistent with a higher intrinsic firing frequency than the LFP theta[20]. Thus, population theta sweeps are thought to be consistent with single cell theta phase precession (i.e., place cells active at early phases having firing fields behind the rat and those active at later phases having firing fields ahead of the rat)[2,3], with the additional requirement of brief experience of the environment[27] and CA3 activity[28]. However, the goal-directed theta sweeps presented here predict theta phase precession when the animal is moving towards the goal, and theta phase procession when the animal moves away from the goal, as shown by simulations of the model (Figure 7a-c). We confirmed this prediction empirically, finding theta phase precession and procession with respect to distance travelled towards or away from the goal, respectively (Figure 7d-e). Thus, in this situation, in which theta sweeps do not simply propagate forwards, single cell phase coding appears to reflect the population dynamics rather than vice versa.

b. Along these lines, it is generally important that a model not only replicates features of the data, but also *explains* something about what causes the system to work. It is not at all surprising that a complex, multi-layered model with many fine-tuned parameters replicates data, or even predicts something about the data that the authors hadn't previously analyzed. The authors need to show under what conditions their model *fails* to replicate the data. Then, based on comparing successful and unsuccessful regions of the parameter space, they might conclude something about what makes the model work. It doesn't help that the authors provide very little justification, and in some cases omit information, about their parameter choices for the model (see Minor Issues).

We thank the reviewer for emphasising the importance of identifying failure regimes. To address this directly, we have added a systematic sensitivity analysis across key parameters. This analysis showed the overall robustness of our model with respect to key parameter changes, whilst explicitly characterising regions of parameter space in which goal-directed sweeps are lost (e.g., when goal-bias is removed/insufficient, and when intrinsic adaptation or theta modulation in place cells, but not directional cells, falls outside the range supporting within-cycle propagation). The aforementioned novel model-based predictions further support the validity of our model. Finally, we emphasise that the model was not fit to the goal-directional effects via optimisation or task-specific training; rather, introducing a goal-directional bias at the directional cell level within

this mechanistic framework reproduces the observed goal-directed sweeps and simultaneously yields additional non-trivial predictions that we confirmed empirically.

c. The authors claim to have two layers of grid cells, one of which is conjunctive and one that is an attractor network. But this is not what they actually simulated! From what I gathered, the "PC network" is simply a layer of place cells that receives goal-biased directional input and input from other place cells. As an excuse for not modeling grid cells, the authors invoke "GPU-memory constraints" (which raises an eyebrow in the era of cloud computing) and "surjective Hebbian plasticity" – a term that they do not explain and that appears nowhere in the literature. The authors either need to model grid cells or very clearly explain why they do not need to do so. Otherwise the text is highly misleading about what the authors actually did.

We thank the reviewer for pointing out the inconsistency between the descriptions of the conceptual model and the actual simulations. We have now provided clear description of what was conceptual and what was actually implemented, and explicitly spell out the computational equivalence of our simplified implementation with the conceptual model in the main text (page 11 line 1-8) and in the Methods subsections on model description (page 40 line 7-10, page 44 line 9-18).

- To extend this account to hippocampal theta sweeps, we proposed a hierarchical extension (Figure 5a), in which an additional goal-oriented allocentric directional input biases directional-cell activities towards the goal direction. Conceptually, this bias would propagate through the grid cell circuitry to drive goal-directed sequential activation in a downstream two-dimensional place cell (PC) attractor network. Here, we explicitly simulate the directional- and place-cell populations as CANs with location-specific inputs, theta modulation and firing rate adaptation, while implicitly including the intermediate grid-cell computations in the coupling from directional cells to place cells (see further details in Methods).

- As discussed in the main text, we only implemented the directional cells ring attractor and the place cells planar attractor in our simulations, instead of the full network. We replaced the computations in grid cell and conjunctive grid cell layers with an additional directionally modulated spatial input to the place cells with equivalent effect.

- Our goal in this work is to test how an allocentric goal-directed bias can propagate through a hierarchical attractor architecture to shape hippocampal theta sweeps, rather than to model the detailed dynamics of entorhinal grid-cell sweeps. In the coupled CAN framework, the entorhinal stage can be viewed as implementing a mapping from an internal directional state (set by the directional cells ring attractor) to a directionally structured spatial drive that steers downstream spatial-encoding activity. When the downstream place cell network receives inputs that are already spatially tuned and directionally shifted as a function of the directional drive received from upstream projections, the specific intermediate implementation (explicit grid cells attractor vs an effective mapping) is not identifiable from place-cell sweep statistics alone. Accordingly, we replace the explicit grid cell layers with the following effective input term to the place cell network, directly projected from directional cells.

2. Issues with the text

Although each of these by itself is minor, together these issues create a paper that is very difficult, or impossible, to read and understand. I'm pointing out particularly egregious issues with the text. In my opinion, almost every sentence in the manuscript should be read out loud and reworked until it makes sense to outside readers.

> We thank the reviewer for in-depth review of the manuscript. Please find below responses to each comment. We have extensively re-written the paper for the revision, hence some of the raised points no longer apply, but we endeavour to provide accurate responses.

"Recent results have suggested a cognitive…" is a sentence fragment. Who is doing the sampling?

> We have updated the statement by starting a new sentence, explicitly stating that it is the sweeps that are performing sampling (page 3 line 8-11).
>
> - Recent results have suggested a cognitive role for theta sweeps. They sample potential paths ahead of the animal, sometimes alternating to both sides of a choice point[6–8] or of the movement direction in open fields[9], are modulated by locations to avoid, and extend farther when running faster[8] or towards more distant[12] or better-learned[13] reward locations.

"Cognitive affordance of theta sweeps". What does this mean? Affordance is an unrelated psychological term from Gibson.

> Thanks for raising this point. Indeed "affordance" could lead to misleading interpretations. Our intention was to raise the question of whether theta sweeps support cognitively relevant planning functions. We have now replaced "cognitive affordance" with "cognitive utility" (page 3 line 17).
>
> - Here we investigated the cognitive role of theta sweeps by analysing place cell firing…

"Due to its cyclic nature…" Confusing/grammatically incorrect sentence

> Thanks for pointing out the ambiguous statement, we have updated the sentence (page 4 line 24-25).
>
> - Due to the cyclic nature of theta sweeps, we analysed the most forward-extending subsequence…

"differential directional-modulatory effects" What does this mean?

> We intended to describe the different modulatory effects of movement direction and goal direction on theta sweeps. We have updated the corresponding paragraph to remove ambiguity (page 6 line 3-7).
>
> - Combining the goal-directionality of the theta sweeps with the (weaker) movement-directionality showed a significantly tighter concentration around the goal direction when animals were moving towards the goal compared to moving away from or at right angles to the goal (Extended Data Figure 4j), both at the level of the individual rat (Extended Data Figure 4k) and of the individual sweep (based on LMM analyses as above; Extended Data Figure 4l).

"Residual sweep direction…" run-on sentence

We have updated the sentence to improve clarity (page 8 line 8-14).

- Accordingly, we compared the concentration of theta sweeps around the goal-direction prior to correct versus incorrect choices after controlling for the effect of distance to goal, either by parametrically regressing out the distance effect with circular-linear correlation (see Methods; Figure 3d) or by subsampling to equalise the numbers of correct and incorrect trials at each distance (Figure 3e). We found significantly stronger alignment between residual sweep direction and goal direction preceding correct compared to incorrect choices, whereas initial offsets (Figure 3d) and sweep lengths did not differ (Extended Data Figure 7j).

"To induce goal-modulation…" another run-on sentence

We have updated the sentence to improve clarity (page 11 line 1-4).

- To extend this account to hippocampal theta sweeps, we proposed a hierarchical extension (Figure 5a), in which an additional goal-oriented allocentric directional input biases directional-cell activities towards the goal direction.

"Given simulated trajectories…" long and very confusing sentence

We have separated the long sentence into shorter sentences to improve readability (page 11 line 9-13).

- Applying our model to simulated trajectories resembling those of rats in the Honeycomb maze (interleaved random walks and rotational scanning periods; Figures 5c; see Methods), the place cell activity successfully reproduced our key empirical findings. Decoded theta sweeps, and their initial offsets, exhibited strong goal-oriented directional bias, and significantly weaker modulatory effects from movement- or head-directions (Figure 5f-g).

"simulated theta sweeps revert to forward and left-right alternation" What does it mean for theta sweeps to revert?

We meant that in the absence of goal-directed inputs to directional cells, the model is equivalent to the base model, hence simulated theta sweeps reverted to left-right alternation around the forward direction. We have updated the draft to remove ambiguity (page 11 line 13-15).

- By contrast, in the absence of top-down goal-directed inputs, the resultant model is equivalent to the base model[19], yielding theta sweeps with left-right alternation about the movement directions (Figure 5b; Extended Data Figure 9d-i).

"Mechanistically, this is due to the joint effect of concurrent movement of the animal during the course of goal-directed sweeps, and reduced firing rate adaptation due to misalignment of sweep- and movement-directions (compared to goalward movement)." What does "this" refer to? What is "concurrent movement"? What is misalignment and sweep and movement directions, and what does it mean to compare misalignment with goalward movement? How does this misalignment reduce firing rate adaptation (if I understood that logical link correctly)? How does all of this explain "this"?

We thank the reviewer for pointing out the ambiguity in our mechanistic explanation of the effects of movement and goal directions. We have updated the associated description to improve clarity (page 11 line 26 - page 12 line 5, page 12 line 6-9):

- The effect of firing rate adaptation interacts with movement direction in interesting ways. Place cells with firing fields behind the rat will have fired more recently than those with fields in front of it and therefore exhibit greater firing rate adaptation. This biases place cell activity in the forward direction from the rat's location, causing the initial offset of goal-directed theta sweeps in the forward movement direction, as seen in the data (Extended Data Figure 9b; cf. Figure 2a).
- The interaction between firing rate adaptation and movement direction causes greater firing during goal-directed sweeps to goals ahead of the movement direction, because they involve place cells that have not fired recently. We also found the same modulation of firing intensity given alignment between movement and goal direction in the data (Extended Data Figure 4i).

"Thus the appearance of theta sequences" is not in a clear logical way related to what was said in the preceding sentence.
"However, the goal-directed sweeps here require a population-level description…" What does this entire sentence mean? What does it mean for sweeps to require something? What does it mean for them to cause something?
"In this case, the goal-directed sweeps…" Again, the logic of this sentence is completely unclear.
"We confirmed this prediction empirically…" Unclear sentence.

Thank you for pointing out the unclear description of the relationship between population responses (theta sweeps) and single-cell phase coding (theta phase precession) in the previous manuscript. We have updated the description to improve clarity (page 12 line 23 - page 13 line 10):

- In rodents running on linear tracks or open fields, place cell firing exhibits "theta phase precession", in which place cells fire at late phases of the theta rhythm when their firing field is ahead of the animal and at successively earlier phases as the animal moves through the field[20], consistent with a higher intrinsic firing frequency than the LFP theta[20]. Thus, population theta sweeps are thought to be consistent with single cell theta phase precession (i.e., place cells active at early phases having firing fields behind the rat and those active at later phases having firing fields ahead of the rat)[2,3], with the additional requirement of brief experience of the environment[27] and CA3 activity[28]. However, the goal-directed theta sweeps presented here predict theta phase precession when the animal is moving towards the goal, and theta phase procession when the animal moves away from the goal, as shown by simulations of the model (Figure 7a-c). We confirmed this prediction empirically, finding theta phase precession and procession with respect to distance travelled towards or away from the goal, respectively (Figure 7d-e). Thus, in this situation, in which theta sweeps do not simply propagate forwards, single cell phase coding appears to reflect the population dynamics rather than vice versa.

"replay might reflect spatial trajectories composed of theta sweeps" What does it mean for replay to reflect trajectories? What does it mean for these trajectories to be composed of sweeps?

We thank the reviewer for pointing out unclear statements describing goal-oriented replays in the previous manuscript. We have updated the associated paragraphs. We meant that replays could activate neurons in sequential orders that reflect connectivity constructed through synaptic plasticity during theta sweeps. Hence under this hypothesis, replays are effectively "compositions" of theta sweeps. The updated description is as follows (page 13 line 19-21):

- … suggests that replay trajectories might be concatenations of trajectories played out during theta sweeps and, in this situation, goal-directed, rather than trajectories of locations that have been actually visited by the rat[13,22].

"towards the goal location and moving closer from the first to second half" Who is doing the moving?

We meant the decoded posterior probabilities moved closer towards the goal from the first to the second half (page 13 line 25 - page 14 line 2).

- The decoded posterior probabilities over-represented locations between the rat and the goal, with decoded locations closer to the goal in the second half of each event than in the first half (Figure 8a, c).

"trajectories reflect the composition of multiple theta sweeps" What does that mean? Later in that sentence, what does it mean for replay to "reflect LTP". What is the logical link between the observation and LTP?

Please see the two responses above for detailed response to the question. We have also updated the description in the main text accordingly to improve clarity (page 14, line 8-12).

- Taken together, our results suggest that goal-directed "replay" sequences are not simple recapitulations of recently experienced trajectories. Instead, they represent trajectories to the goal perhaps shaped by plasticity between place cells with adjacent fields that are activated during theta-sweeps[22], effectively linking multiple theta sweeps into longer replay trajectories[13,22].

"Complementing fan-tail representations of preferred goal directions…" What does that mean?

We meant the recently discovered goal-oriented egocentric directional tuning in hippocampal place cells (the fan-tail shaped egocentric directional tuning map; Ormond & O'Keefe, 2022). Given the fact that we have additionally accounted for the egocentric directional tuning in place cells with our hierarchical continuous attractor network model, we have removed this statement in the updated manuscript (page 14 line 24 - page 15 line 2):

- These vectors may represent a pure cognitive goal vector encoding the direction and distance to the goal location irrespective of immediate sensory or movement constraints, and potentially support flexible online planning strategies.

"Building upon an existing…" Run-on sentence; unclear what "which" refers to

Thanks for pointing this out. We have updated the sentence to improve clarity (page 15 line 4-9).

- Building upon an existing continuous attractor network model that captures forward and left-right alternating theta sweeps in parasubicular-entorhinal circuitry[19], we proposed a

hierarchical extension to include place cells and an additional goal-directed top-down input to directional cells. Our model successfully reproduced key characteristics of goal-directed theta sequences without requiring task-specific training or parameter optimisation.

"potentially reflecting their affordance of plasticity across place cell assemblies" What does this mean?

We meant that our results suggest that population dynamics within theta cycles (theta sweeps) support the formation of assembly connectivity. We have updated the description to improve clarity (page 15 line 18-21).

- We found that "replay" sequences during immobility on the Honeycomb maze, in which no direct goalward trajectories physically occurred, were also goal-directed, like the theta sweeps. This supports the idea that these trajectories are formed by plasticity between place cells activated during theta sweeps[3,13,23], consistent with hypotheses that they are concatenations of theta sweep segments[22,29,34].

The tense is constantly mixed up in the paper. The authors sometimes switch between past and present several times per paragraph.
Please correct numerous typos and grammatical issues like is/are confusion. There were too many for me to mark. There is software these days that will do a good job detecting problems automatically.

We thank the reviewer for their careful scrutiny of the manuscript, we have updated the manuscript to correct all typos, grammatical issues, and tense inconsistencies in the previous manuscript.

MINOR ISSUES

Discussion. "location-independent sense of direction" What results show location independence?

We were referring to the fact that theta sweeps extend towards the goal regardless of the animal's location on the maze (e.g. the roulette plot Figure 1f). However, we agree that "location-independent sense of direction" is unclear (as sense of direction does depend on location) and have removed this phrase.

Discussion of mammalian evolution in the last paragraph is very questionable. If anything, a growing body of work suggests highly species-specific processing. See primates (very little spatial coding, if any; very little theta, if any) and bats (place cells, no theta).

Perhaps goal-directed nonlocal coding in the hippocampus is a general principle. But the details that the authors refer to here might be specific to the ecological niche of mice and rats. If the authors are referring to the general principles, then they likely did not emerge "early in mammalian evolution": see results on place cells in birds and fish.

The cited references were included to indicate the evidence for theta phase precession and theta sweeps in humans and bats, respectively, albeit that the LFP theta has a much more variable frequency and amplitude in humans and a strong relation to wing beats in bats. We thank the reviewer for their question, and we agree that "emerging early in mammalian evolution" is more

specific than we intended, given findings of place cells in birds and fish. We have updated the statement to improve accuracy (page 15, line 24-25).

- The conservation of theta rhythms and place cell-like representations across mammals suggests that goal-directed sequential processing represents a fundamental computational principle[45–47].

Why do the authors not make circular histograms for head direction tuning? These are mentioned in the text but not shown. Given the emphasis on head direction vs. goal direction, these should be presented more extensively side-by-side.

We thank the reviewer for pointing out the missing distributions of relative angles between sweep and head directions. We have now included histograms of these distributions in all relevant figures (Figure 1g-j, Figure 4c-f, Extended Data Figure 8i-l, Extended Data Figure 9e-h, Supplementary Figure S2b-e, Supplementary Figure S3b-e).

“Discovered subicular… circuitry” isn’t quite right

We agree with the reviewer on the usage of “Discovered”, and have decided to remove this statement from the abstract.

Result in Fig. 3g is critical because it’s predicted by the model. But it’s not clear where the result of the model is and how it compares. A better statistical treatment of this effect is needed.

We thank the reviewer for noting the missing of the empirical data of sweep length under varying relative movement direction to the goal. The plot is now presented in Figure 2a.

In Eq. 4, shouldn’t 1/N be 1/sqrt(N). Or is 1/N correct here?

1/N is correct since it is by definition the mean of vector lengths prescribed by the angles.

The authors should clearly state the number of units used in the network, as well as all the other parameters needed to implement the model.

All default model parameters were provided in Table S1 at the end of the previous manuscript. We implemented the hierarchical CAN model with 100 directional cells and 2500 place cells (50 $\times$ 50 with equal-spaced field centers). In the current manuscript, we further provide parameter ranges used in the sensitivity analysis (Extended Data Figure 9, Supplementary Table S1).

Eqns. 9 and 13 appear to be missing a threshold operation, otherwise one simply has a quadratic activation function, which would be a very strange choice.

We thank the reviewer for the careful reading. In our original implementation, we used an original quadratic divisive normalisation non-linearity, following the classical definition in standard CAN formulations (Wu, Hamaguchi, Amari, 2008). To avoid ambiguity about negative inputs, we evaluated models implemented by replacing the raw division normalisation by firstly transforming the pre-synaptic inputs through a ReLU non-linearity before applying divisive normalization. Incorporating the additional ReLU non-linearity yielded indistinguishable results for the main simulations (goal-directed bias, sweep length effects, phase coding). To avoid further

ambiguity about non-negative firing neurons, we adopted the ReLU non-linearity in all our current simulations. We have updated equations S9 and S13 accordingly.

Eq. 13 has a typo in the numeration. I believe it should be g h_j^2

We thank the reviewer for pointing out the notational error. The correct numerator should be $g \cdot [p_j]_+^2$, where p_j denotes the pre-synaptic input to the j-th place cell, g denotes the gain factor, and $[\cdot]_+ = \max(\cdot, 0)$ denotes the ReLU non-linearity.

Using brackets to denote Euclidean distance is unusual, since these are usually used for inner products or for expectations. It is better to use norm notation: dist(s_i,s_j) = ||s_i – s_j||

We thank the reviewer for the formatting suggestion. We have replaced all Euclidean distance operation with the norm notation.

Is modulo 2pi missing in the distance operations?

We described $|\psi_i - \theta_t|$ as the circular distance between the two angles, hence the modulo 2pi is not strictly necessary. However, we agree with the reviewer that explicitly defining the circular distance function as $|\psi_i - \theta_t| = \min(mod(\psi_i - \theta_t, 2\pi),\ 2\pi - mod(\psi_i - \theta_t, 2\pi))$ by incorporating the modulo 2pi would help with readability. We have updated the text following equation S6 to include this definition.

- where ψ_i represents the preferred internal direction of i-th neuron (in radians), $|\psi_i - \theta_t| = \min(mod(\psi_i - \theta_t, 2\pi),\ 2\pi - mod(\psi_i - \theta_t, 2\pi))$ represents circular distance between the internal direction for the i-th neuron and current head direction…

What is omega_t ?

ω_t denotes the phase of the ongoing theta rhymicity at time t, which was defined after Equation S7 in the previous manuscript. We have also included its definition after Equation S18 to improve readability.

Fig. 1h campus should be same as (g) not same as (f)

We thank the reviewer for pointing out the erroneous statement. We have updated the caption to correct it.

The claim "The DC attractor… driving PC activity bump towards the goal direction" is not true. None of these features are actually present in the model.

We thank the reviewer for pointing the inconsistency. As discussed in responses to early comments from the reviewer, we have separated the description into the conceptual model and actual implementations, and have updated the captions accordingly (page 24 line 7-14).

- Conceptually, the DC attractor projects directional input with goal-oriented bias to the conjunctive grid cells[51], and subsequently drives shift in the activity bump in the downstream grid-cell (GC) attractor network along the goal direction. The GC attractor projects linearly onto the hippocampal place cells (PC), driving population PC activity bump towards the goal direction. The presented schematic illustrates the conceptual hierarchical computations

underlying the model, and our simulations used an effectively simplified version with explicitly implemented DC and PC networks, and equivalent computations that implicitly capture the net influence of intermediate conjunctive and standard grid cell layers (see main text and Methods).

If this paper is submitted again, please include line numbers.

We thank the reviewer for pointing out this important formatting issue. We have included line numbers in the current manuscript.

Here we provide point-to-point response to reviewer 3's remaining comments, with the reviewer's comments in black, our response in blue, and referenced changed texts in green.

Response to remaining comments from Reviewer 3

The new version of the manuscript is a substantial improvement from the original and addresses some of my concerns. In particular, the manuscript is now more readable, softens some of the most egregious claims, and is more transparent about the model implementation.

- We are glad to see that reviewer 3 have recognised improvements in the manuscript during the last revision.

Unfortunately some of my central concerns about the model – currently a major component of the paper – remain, in part because they are now confirmed by the authors themselves. The model is not truly a mechanistic circuit model, since the grid cell layer is not actually modeled. The authors have truthfully removed those claims. Its explanatory power about the circuit is therefore extremely limited if not nonexistent.

- As we stated in the previous revision, the primary aim of the model is not to investigate the precise mechanistic role afforded by the entorhinal grid cells, but as a necessary information processing step in conveying goal-directed information to place cells firing. Hence, for the purpose of our model, we believe replacing the entorhinal layers with a functionally equivalent mapping will not change our main claims. We do acknowledge that grid cells potentially plays a non-trivial part in facilitating efficient navigation within the subicular-entorhinal-hippocampal circuitry, but characterising its exact mechanistic contribution is beyond the scope of the present work.

More importantly, the authors have now admitted that the central claim of the model – that there is goal tuning in neural activity – is a trivial consequence of a strong goal input into the network. Without this central claim, and without a clear mechanistic link to biological circuits, I struggle to see what the contribution of the model is. As I wrote in my original review, demonstrating that some arbitrary model replicates some features of data is a very weak scientific contribution. There could be dozens of broad classes of models that replicate the same features of the data. There needs to be some explanation of *what* causes the model to work and, conversely, what could cause other plausible models to fail.

- We argue that, despite the reviewer's objection, the fact that incorporating goal-directed inputs in the proposed hierarchical CAN not only induces goal-directed theta sweeps, but also yields a number of non-trivial predictions that were subsequently validated in real data, demonstrates the generality of the proposed model. We expand on this in the discussion section in the updated manuscript, as following (page 15, line 13-20).

  Notably, the additional goal-oriented input does not merely impose a goalward firing-rate bias; it modulates population dynamics whilst preserving constraints from sensorimotor input, theta modulation, and firing rate adaptation. This leads to a number of non-trivial predictions, including rotational goal-anticipatory firing, a reversal between theta phase

precession and procession depending on alignment between goal and movement directions, and the emergence of goal-directed egocentric directional tuning in individual place cells19. The proposed mechanism unifies goal-directed coding and theta phase coding on both population and single-cell levels, and identifies circuit-level variables whose causal contributions can be tested in future experiments.

I am not convinced by the authors' sensitivity analysis in the revision, which frankly feels like a cosmetic change to try appeasing a critical reviewer. The analysis is one-dimensional, changing one parameter at a time, and it shows that the conclusions are not horribly dependent on the exact values of the parameters. But it still fails to provide any explanatory power to the model. What exactly have we learned about what causes models to work or not work – that is a litmus test of modeling that the manuscript fails.

- We agree that the sensitivity analysis alone is not intended to provide the main explanatory value of the model. Its purpose was limited to showing that the qualitative model behaviour is not an artefact of extensive hyperparameter tuning, which was one of the main criticisms in the reviewer's initial review.

It would help if some predictions of the model were truly unexpected or demonstrated to be difficult to obtain in computational models. But unfortunately most of the predictions seem to be very expected consequences of model construction (e.g. that stronger theta modulation produces longer sweeps, given that theta modulation in the model is what produces the sweeps). The only somewhat interesting prediction is phase precession vs. procession depending on the movement direction. But, again, without a strong mechanistic explanation I'm not sure that the model is viable simply based on this one prediction."

- Our above reply on justifying the significance of additional mechanistic predictions applies here as well. Additionally, we now provide a clearer description of the mechanistic reasoning behind goal-dependent phase coding in the updated manuscript (page 13, line 5-9).

    However, the goal-directed theta sweeps presented here predict theta phase precession when the animal is moving towards the goal, and theta phase procession when the animal moves away from the goal (Figure 7a-c). This is because, during sweeps towards a goal located behind the rat, place cells active at early phases have firing fields ahead of the rat and those active at later phases have firing fields ahead of behind the rat and closer towards the goal.
